# Evidence-Based Clinical Recommendations on the Appropriateness of Prescriptions and Diagnostic Tests in Paediatric Allergology: Focus on IgE-Mediated and Non-IgE Mediated Food Allergy, Eosinophilic Gastrointestinal Disease, Urticaria, Angioedema and Atopic Dermatitis

**DOI:** 10.3390/children13091146

**Published:** 2026-08-26

**Authors:** Valentina Fainardi, Matteo Riccò, Rachele Antignani, Simona Bellodi, Mauro Calvani, Fabio Cardinale, Elena Chiappini, Maria Angiola Crivellaro, Daniela Cunico, Massimiliano Esposito, Antonella Giudice, Roberto Grandinetti, Amelia Licari, Michele Miraglia Del Giudice, Maria Marsella, Alberto Martelli, Anna Montanari, Iria Neri, Rita Nocerino, Elisabetta Palazzolo, Diego Peroni, Cristina Piersantelli, Giuseppe Pingitore, Giuseppe Squazzini, Mariangela Tosca, Carlo Caffarelli, Susanna Esposito

**Affiliations:** 1 Paediatric Clinic, Department of Medicine and Surgery, University of Parma, 43125 Parma, Italy; valentina.fainardi@unipr.it (V.F.); antonella.giudice@unipr.it (A.G.); anna.montanari@unipr.it (A.M.); elisabetta.palazzolo@unipr.it (E.P.); carlo.caffarelli@unipr.it (C.C.); 2 Servizio di Prevenzione e Sicurezza Negli Ambienti di Lavoro (SPSAL), Azienda Unità Sanitaria Locale—Istituto di Ricovero e Cura a Carattere Scientifico (AUSL-IRCCS) Reggio Emilia, 42122 Reggio Emilia, Italy; matteo.ricco@ausl.re.it; 3 Primary Care Pediatricians, Società Italiana Medici Pediatri (SIMPE), 64100 Teramo, Italy; r.antignani@virgilio.it (R.A.); simona.bellodi@gmail.com (S.B.); squazzo1957@gmail.com (G.S.); 4 Società Italiana di Allergologia e Immunologia Pediatrica (SIAIP), 20126 Milano, Italy; maurocalvani58@gmail.com (M.C.); fabiocardinale1961@gmail.com (F.C.); amelia.licari@unipv.it (A.L.); diego.peroni@unipi.it (D.P.); mariangelatosca@gaslini.org (M.T.); 5 UOC Pediatria, Ospedale S. Camillo-Forlanini, 00152 Roma, Italy; 6 UOC Pediatria, Ospedale Pediatrico “Giovanni XXIII”, 70126 Bari, Italy; 7 UOC Malattie Infettive, Ospedale Meyer, 50139 Firenze, Italy; elena.chiappini@unifi.it; 8 Società Italiana di Pediatria Preventiva e Sociale (SIPPS), 81031 Aversa, Italy; 9 UOSD Allergologia, Azienda Ospedale Università di Padova, 35122 Padova, Italy; mariaangiola.crivellaro@aopd.veneto.it; 10 Società Italiana di Medicina Legale e delle Assicurazioni (SIMLA), 00186 Roma, Italy; massimiliano.esposito@unikore.it; 11 Medicina Legale, Università degli Studi di Enna, 94100 Enna, Italy; 12 UOC Clinica Pediatrica, IRCCS Policlinico San Matteo, 27100 Pavia, Italy; 13 Department of Woman, Child and General and Specialized Surgery, Università della Campania Luigi Vanvitelli, 80131 Napoli, Italy; 14 Società Italiana Gestori del Rischio in Sanità (SIGeRiS), 00186 Roma, Italy; maria.marsella@aornmoscati.it; 15 UOC di Pediatria Azienda Ospedaliera di Rilievo Nazionale San Giuseppe Moscati di Avellino, 83100 Avellino, Italy; 16 Network delle Associazioni di Allergologia Pediatrica, Italy; albmartelli@tiscali.it; 17 Società Italiana di Dermatologia Pediatrica (SIDeRP), 25124 Brescia, Italy; iria.neri@aosp.bo.it; 18 UOC Clinica Dermatologica, IRCCS Azienda Ospedaliera Universitaria di Bologna, 40138 Bologna, Italy; 19 Società Italiana di Pediatria Infermieristica (SIPINF), 00186 Roma, Italy; rita.nocerino@unina.it; 20 Clinica Pediatrica, Università di Pisa, 56126 Pisa, Italy; 21 Società Italiana delle Cure Primarie Pediatriche (SICuPP), 10146 Torino, Italy; cristinapiersantelli@libero.it; 22 AAIITO (Associazione Allergologi Immunologi Italiani Territoriali e Ospedalieri), 50127 Firenze, Italy; giuseppe.pingitore@gmail.com; 23 Centro di Allergologia, IRCCS Giannina Gaslini, 16147 Genova, Italy

**Keywords:** allergy, children, food allergy, urticaria, recommendations, appropriateness, equity, cost, angioedema, atopic dermatitis

## Abstract

**Highlights:**

**What is the main finding?**

- This paper presents evidence-based clinical recommendations addressing the appropriate use of specialist consultations and diagnostic investigations in children with IgE-mediated and non-IgE-mediated food allergies, eosinophilic gastrointestinal diseases, urticaria, angioedema and atopic dermatitis.

**What are the implications of the main finding?**

- Diagnostic investigations in pediatric allergology should be guided by clinical history and structured pathways rather than performed routinely.
- These recommendations may reduce inappropriate prescriptions, improve access to specialist care, and promote more equitable and sustainable pediatric allergy services.

**Abstract:**

**Background:** Appropriate diagnostic testing is essential to quality pediatric allergy care, affecting diagnostic accuracy, treatment decisions, resource use, and patient outcomes. This study aimed to develop evidence-based recommendations for specialist consultations and diagnostic investigations in children with food allergies, eosinophilic gastrointestinal diseases, urticaria, angioedema, and atopic dermatitis. **Methods:** A multidisciplinary panel convened for the Italian National Institute of Health formulated and prioritized clinical questions using the PICO framework and structured expert consensus. A systematic review of the literature from 2015 to 2026 informed the recommendations, with certainty of evidence assessed using GRADE methodology. **Results:** In food allergy, sensitization tests alone are insufficient for diagnosis, while oral food challenges remain central to diagnostic confirmation. Endoscopic biopsies are essential for diagnosing eosinophilic gastrointestinal diseases. In chronic urticaria, specialist consultation is recommended, whereas routine allergy testing should be avoided without a suggestive clinical history. In angioedema, distinguishing histaminergic from bradykinin-mediated forms is critical, with targeted laboratory testing for hereditary or acquired conditions. In atopic dermatitis, standardized severity assessment should guide management and consideration of biologic therapies, while patient and family education represent key components of care. **Conclusions:** These recommendations provide a framework for standardized pediatric allergy diagnostic pathways, aiming to improve quality and equity of care while reducing unnecessary investigations and optimizing healthcare resource use.

## 1. Introduction

The appropriateness of prescribing clinical and instrumental investigations is essential for the effective management of healthcare resources, ensuring consistency, equity, and timely access to specialist services. Conversely, inappropriate prescriptions generate unnecessary costs and delays for patients and their families while placing an avoidable burden on specialists and the healthcare system. Therefore, the development of clear, evidence-informed, and consensus-based recommendations is crucial to improve the quality of care, optimize resource utilization, and ultimately support the long-term sustainability of the National Health Service [1].

In pediatric allergology, Good Clinical Practice Recommendations (GCPRs) are particularly valuable for guiding the diagnostic work-up and management of complex conditions such as food allergies, eosinophilic gastrointestinal diseases, urticaria, angioedema, and atopic dermatitis [2,3,4,5].

The development of robust and clinically relevant GCPRs requires several fundamental principles to be fulfilled. First, each recommendation should address a clearly defined clinical question using standardized terminology. Second, recommendations should be aligned with current professional standards and supported by transparent scientific rationale. Third, the supporting evidence should be critically appraised according to its methodological quality, consistency, and applicability to clinical practice. Finally, recommendations should be feasible to implement in routine healthcare settings. When these requirements are met, GCPRs are expected to provide valid, reliable, reproducible, clinically applicable, and clearly interpretable guidance. Importantly, their development should rely on a multidisciplinary approach involving all relevant clinical specialties.

The objective of the present work was therefore to develop national recommendations based on the best available scientific evidence to reduce unwarranted variability in the prescription of diagnostic investigations, harmonize clinical practice, and promote equitable access to specialist care for children with allergic diseases. The present work completes a series of previously published studies addressing GCPRs in pediatric allergology [6,7]. Together, these complementary contributions provide a comprehensive evidence-based framework for improving the appropriateness of diagnostic and clinical pathways across the major conditions encountered in pediatric allergology. More precisely, in the present document, evidence-based recommendations on the appropriateness of prescriptions and diagnostic tests for IgE-mediated and non-IgE mediated food allergy, eosinophilic gastrointestinal disease, urticaria, angi-oedema and atopic dermatitis will be gathered and addressed.

## 2. Materials and Methods

### 2.1. Working Group and Panel of Experts

The present document is the result of the joint work of a multidisciplinary panel, which includes representatives of paediatrics scientific societies (Italian Society of Paediatricians [Società Italiana Medici Pediatri, in Italian; SIMPE], Italian Society of Pediatrics [Società Italiana di Pediatria, in Italian; SIP], Italian Society of Paediatric Allergology and Immunology [Società Italiana di Allergologia e Immunologia Pediatrica, in Italian; SIAIP], Italian Society for Childhood Respiratory Diseases [Società Italiana per le Malattie Respiratorie Infantili, in Italian, SIMRI], Italian Society of Pediatric Emergency Medicine [Società Italiana di Medicina di Emergenza e Urgenza Pediatrica, in Italian; SIMEUP], Italian Society of Pediatric Primary Care [Società Italiana delle Cure Primarie Pediatriche, in Italian; SICuPP]); representatives of scientific societies of specialists involved in the care of allergic children (Italian Society of Pediatric Dermatology [Società Italiana di Dermatologia Pediatrica, in Italian; SIDerP], Italian Association of Local and Hospital Allergists and Immunologists [Associazione Allergologi Immunologi Italiani Territoriali e Ospedalieri, AAITO]), as well as experts in legal medicine from the Legal and Insurance Medicine (Società Italiana di Medicina Legale e delle Assicurazioni, in Italian; SIMLA), and in telemedicine from Italian Telemedicine Society, (Società Italiana di Telemedicina, in Italian; SIT).

In order to ensure a multidisciplinary approach to the development of GCPRs on IgE-mediated and non-IgE-mediated food allergies, eosinophilic gastrointestinal diseases, urticaria, angioedema and atopic dermatitis, a multi-professional working group was established. The panel included pediatric allergists, general pediatricians, general practitioners, clinical immunologists, otolaryngologists, dermatologists, clinical pharmacologists, psychologists, pediatric nurses, methodologists, and representatives of patient associations, thereby ensuring that the recommendations also addressed the practical needs and perspectives of affected children and their families. The present work forms part of a broader initiative coordinated by the Italian National Institute of Health (Istituto Superiore di Sanità, ISS) to develop evidence-based Good Clinical Practice Recommendations for the appropriate prescription of diagnostic investigations and healthcare services in pediatric patients with allergic diseases.

### 2.2. Identification of Clinical Questions

As a preliminary step, the working group identified and refined the research questions addressing the diagnostic management of IgE-mediated and non-IgE-mediated food allergies, eosinophilic gastrointestinal diseases, urticaria, angioedema and atopic dermatitis in children and adolescents. Clinical questions were formulated according to the Population/Patient, Intervention, Comparator, Outcome (PICO) framework [8,9,10].

Each member of the working group was invited to propose up to five PICO questions for each thematic area. All proposed PICOs were subsequently discussed and independently rated by the panel using a 10-point Likert scale (0–3 = disagreement; 4–6 = moderate agreement; 7–9 = strong agreement). Only PICO questions achieving a mean score > 7 were ultimately selected for evidence synthesis and recommendation development. The methodological protocol was defined a priori by the multidisciplinary working group within the broader initiative coordinated by the Italian National Institute of Health (Istituto Superiore di Sanità, ISS).

### 2.3. Systematic Review

To answer the clinical questions selected by the expert panel, systematic literature reviews were undertaken for each PICO question. Searches were performed in three electronic databases (MEDLINE, Embase, and the Cochrane Library) using combinations of general terms (e.g., allergy, atopy, allergic disease) and condition-specific keywords. Detailed search strategies for each PICO are reported in Table A1. The following inclusion criteria were applied:

- Study design: primary studies were limited to randomized controlled trials (RCTs), prospective or retrospective cohort studies with a comparator, and case–control studies. Secondary sources, including systematic reviews (with or without meta-analysis) and clinical practice guidelines, were initially retrieved and subsequently screened to identify additional eligible studies through backward citation tracking (snowballing). Case reports, letters to the editor, brief reports, conference abstracts, and other non-peer-reviewed publications were excluded.
- Population: only studies including children and adolescents aged 0–18 years were considered eligible.
- Language and publication period: to ensure that the recommendations reflected contemporary clinical practice, the search was limited to articles published in English between January 2015 and January 2026.

According to the Preferred Reporting Items for Systematic Reviews and Meta-Analyses (PRISMA) statement [8,9,10], suitable articles were initially pooled together. After the removal of duplicates, two researchers conducted title and abstract screening independently and blindly. Articles fulfilling inclusion criteria were then similarly blindly reviewed by two researchers. Conflicts in each state of the systematic review were resolved through discussion with a third researcher.

The risk of bias of observational studies was assessed using the Newcastle–Ottawa Scale (NOS), which provides an overall score ranging from 0 to 9 points and is widely recommended for the quality appraisal of observational studies included in systematic reviews [11]. The NOS evaluates three main methodological domains: selection of study participants, comparability of study groups, and ascertainment of either the exposure (case–control studies) or the outcome (cohort studies). Although the original NOS was developed for cohort and case–control studies, no specific version has been formally validated for cross-sectional studies. Given the methodological similarities between cohort and cross-sectional designs, differing primarily in the temporal relationship between exposure and outcome assessment [12] the cohort version of the NOS was also applied to cross-sectional studies.

Two reviewers independently assessed the methodological quality of all eligible studies. Disagreements were resolved through discussion and consensus whenever possible; when consensus could not be reached, a third reviewer acted as an arbitrator. Studies were classified according to the following predefined thresholds: 0–3 points, low methodological quality; 4–6 points, moderate methodological quality; and 7–9 points, high methodological quality. To provide the most comprehensive synthesis of the available evidence, all studies fulfilling the predefined inclusion criteria were retained in the review regardless of their methodological quality. Study quality was subsequently taken into account during the GRADE assessment and formulation of the recommendations.

The risk of bias of RCTs was assessed using the Risk of Bias (ROB) tool developed by the National Toxicology Program (NTP) Office of Health Assessment and Translation (OHAT), now the Health Assessment and Translation (HAT) Program [13,14]. The OHAT ROB tool rates the risk of bias across six methodological domains using a four-level scale (“definitely low”, “probably low”, “probably high”, and “definitely high”): participant selection (D1), confounding (D2), attrition/exclusion (D3), detection (D4), selective reporting (D5), and other potential sources of bias (D6).

The OHAT ROB tool was selected because it provides a domain-based assessment of methodological quality without relying on a single summary judgment, thereby allowing a more transparent appraisal of the strengths and limitations of individual studies. Consistent with the study protocol, risk-of-bias assessments informed the certainty of the evidence during the GRADE process but were not used as exclusion criteria for study selection [14].

The methodological quality of the included systematic reviews and clinical practice guidelines was assessed using adapted versions of the AMSTAR 2 tool and the AGREE II instrument, respectively [15,16].

### 2.4. Data Extraction

Articles meeting all inclusion criteria were then selected for data extraction, including title, authors, journal, and year of publication, target population (by disease and age), type of intervention and comparator, and results. Where available, numerical data were extracted for possible meta-analysis; if unavailable, results were reported narratively.

### 2.5. Meta-Analysis

In order to ensure a minimum degree of robustness and interpretability of pooled estimates, whenever at least five studies evaluated the same intervention in comparable populations and reported the same outcome, a quantitative synthesis (meta-analysis) was performed. For dichotomous outcomes, pooled effect estimates from randomized controlled trials were expressed as Relative Risks (RRs) with their corresponding 95% confidence intervals (95%CIs), whereas Odds Ratios (ORs) with 95%CIs were calculated for observational studies. For continuous outcomes, pooled estimates were expressed as either the Mean Difference (MD) or the Standardized Mean Difference (SMD), depending on whether the included studies used the same or different measurement scales.

Given the expected clinical and methodological heterogeneity across studies, pooled estimates were calculated using random-effects models. Between-study variance (τ^2^) was estimated using the restricted maximum-likelihood (REML) method, and statistical heterogeneity was assessed using Cochran’s Q and quantified by the I^2^ statistic. I^2^ values were interpreted as indicating low (<25%), moderate (25–50%), substantial (50–75%), or considerable (>75%) heterogeneity [17,18,19]. All statistical tests were two-sided, and *p* values < 0.05 were considered statistically significant.

Meta-analyses were undertaken only when sufficient quantitative information was available, including the sample size, mean value, and standard deviation for each comparison group. When standard deviations were not directly reported but could be derived from other summary statistics (e.g., confidence intervals), they were estimated according to standard statistical procedures [20]. When essential data required for quantitative synthesis were unavailable, the corresponding authors were contacted by e-mail to request the missing information.

The meta-analysis was conducted by means of R (version 4.3.1) [21] and Rstudio (version 2023.06.0 Build 421; Rstudio, PBC; Boston, MA, USA) software by means of the packages meta (version 7.0) and fmsb (version 0.7.5).

### 2.6. GRADE and GRADE Evidence to Decision Framework

The certainty of the evidence was assessed using the Grading of Recommendations Assessment, Development and Evaluation (GRADE) approach. For each PICO and each critical outcome, the certainty of evidence was initially determined according to study design and subsequently downgraded by one or two levels when serious or very serious concerns, respectively, were identified for risk of bias, inconsistency, indirectness, imprecision, or publication bias. Conversely, where applicable, certainty from non-randomized evidence could be upgraded in the presence of a large magnitude of effect, a dose–response gradient, or residual confounding likely to reduce rather than increase the observed effect. Final certainty was categorized as high, moderate, low, or very low.

The formulation and strength of recommendations followed the GRADE Evidence-to-Decision (EtD) framework [22] and were not determined solely by the certainty of evidence. For each intervention within each PICO question, the multidisciplinary panel systematically considered the priority of the health problem, desirable and undesirable effects, certainty and directness of the available evidence, patients’ values and preferences, resource requirements and cost-effectiveness, equity, acceptability, and feasibility. Recommendations were subsequently formulated through structured discussion and consensus among panel members. Accordingly, recommendation strength was not assigned through a direct correspondence with the GRADE certainty rating. Strong recommendations were issued only when the overall EtD assessment clearly supported or opposed an intervention. Thus, moderate-certainty or, where justified by the overall EtD assessment, low-certainty evidence could support a strong recommendation when the expected benefits clearly outweighed potential harms or burdens, particularly when the consequences of delayed or inappropriate diagnosis were considered clinically relevant. Conversely, conditional recommendations were preferred when uncertainty remained regarding the magnitude of benefit, applicability, resource requirements, or directness of the supporting evidence. Recommendations relying predominantly on indirect or limited evidence were therefore generally formulated as conditional or consensus-based.

### 2.7. Consensus Panel for the Strength of Recommendations

The strength of each recommendation was determined through a structured consensus process involving all members of the multidisciplinary panel. Once draft recommendations had been formulated, an electronic survey was distributed to all panel members. Using a 10-point Likert scale (0–3 = low strength, 4–6 = moderate strength, 7–9 = strong recommendation), participants were asked to rate the strength of each recommendation. In addition, the expected sustainability of each recommendation was independently assessed using a second 10-point Likert scale (0–3 = not sustainable, 4–6 = probably sustainable, 7–9 = highly sustainable). Mean scores were subsequently calculated and used to determine the final strength of the recommendations. When substantial disagreement emerged among panel members, the corresponding recommendations were further discussed until consensus was reached.

## 3. Results

### 3.1. IgE-Mediated and Non-IgE Mediated Food Allergy and Eosinophilic Gastrointestinal Disease

#### 3.1.1. Summary of Literature Search

**Figure 1:**
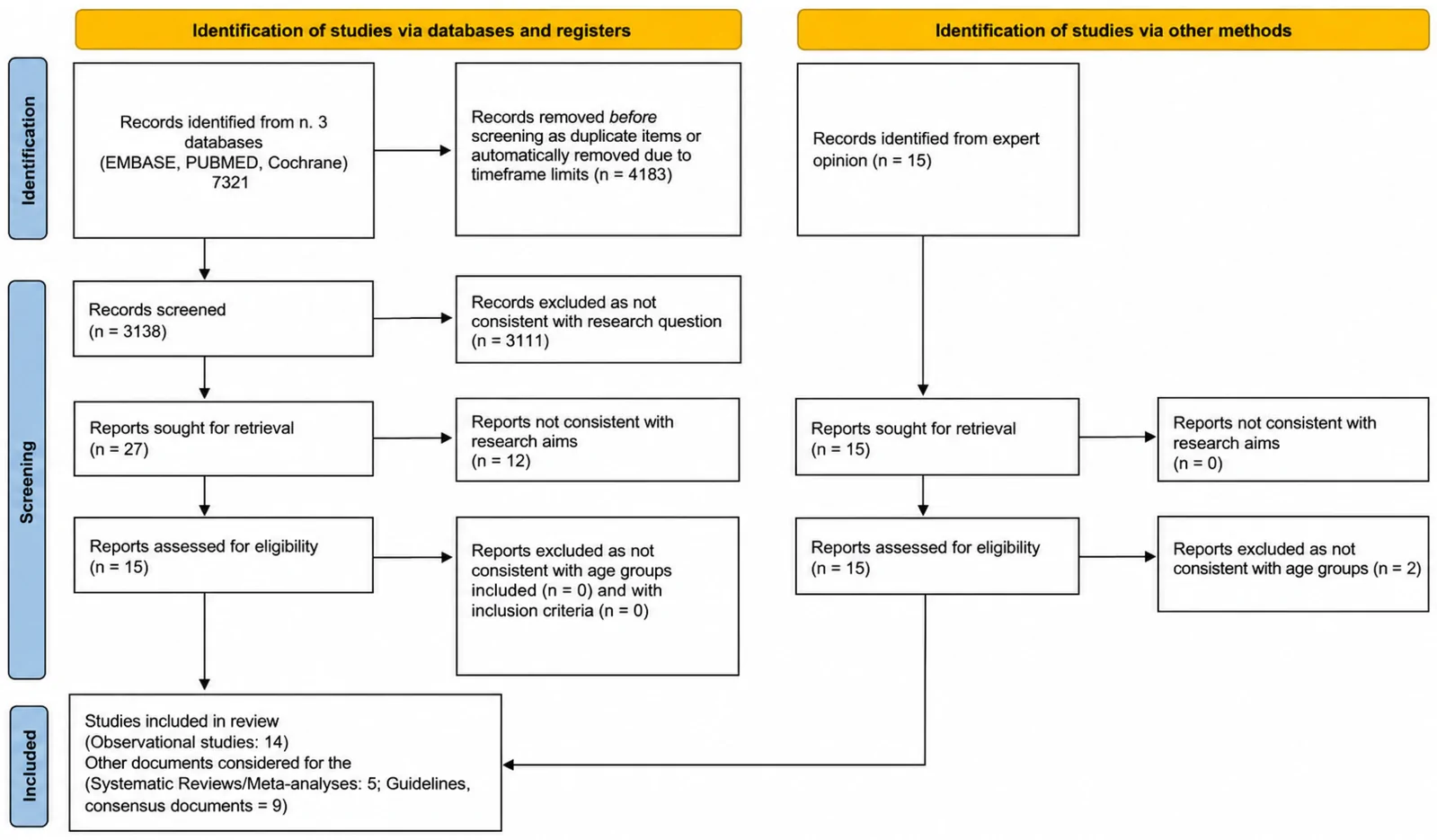
Flow-chart of included studies on the topic of IgE-mediated and non-IgE mediated food allergy and eosinophilic gastrointestinal disease. A total of 28 documents were included, comprising 15 records identified through database searches and 13 identified through expert consultation. Irrespective of their source of identification, the final evidence base comprised 14 observational studies, 5 systematic reviews/meta-analyses, and 9 guidelines or consensus documents.

The search of three electronic databases (Embase, MEDLINE, and the Cochrane Library) identified a total of 7321 records. After removing duplicates (*n* = 4183), 3138 records were screened by title and abstract, resulting in the exclusion of 3111 records. Full-text articles were retrieved for the remaining 27 studies. Of these, 15 met the predefined inclusion criteria and were included in the qualitative synthesis. An additional 13 relevant publications were identified through expert consultation and citation tracking, resulting in a total of 28 studies included in the evidence synthesis (Figure 1).

The original studies included in the search are reported and described in Table A2, while Systematic Reviews and Guidelines are reported in Table A3 and Table A4. Briefly, fifty percent of the included studies were observational studies, while the remaining were meta-analyses and guidelines/recommendations. No RCTs (Randomized Controlled Trials) were found that addressed the question. Quality appraisal of included studies is summarised in Table A5, Table A6, Table A7 and Table A8.

The outcomes considered depending on the PICO framework mainly included:

- Diagnostic accuracy through allergy tests to identify the causative agent and initiate specific therapy;
- Quality of life measured through standardized questionnaires.

#### 3.1.2. Clinical Questions

The full list of inquired PICOs is provided by Table 1.

##### PICO 1

In children and adolescents with suspected food allergy and atopic dermatitis, is an allergy consultation recommended over the regular follow-up with a primary care pediatrician to initiate specific therapy and improve quality of life?

Narrative report. Food allergy frequently coexists with atopic dermatitis in children. While the primary care pediatrician plays a key role in the early recognition and initial management of suspected food allergy, referral to a pediatric allergy specialist may improve diagnostic accuracy, optimize therapeutic decisions, and ultimately enhance the quality of life of both patients and their families.

Current EAACI guidelines recommend an individualized diagnostic approach, particularly in children at high risk of food allergy, such as those with moderate-to-severe atopic dermatitis [23,24]. Specialist evaluation allows comprehensive risk assessment and access to second-level diagnostic investigations, including skin prick tests (SPT), measurement of serum-specific IgE, and, when indicated, oral food challenge (OFC), which remains the diagnostic gold standard and should be performed by experienced personnel in an appropriate clinical setting [23].

Because SPT and serum-specific IgE evaluate different aspects of allergic sensitization, their results may not always be concordant, particularly in younger children whose immune responses are still developing. Consequently, these tests should not be interpreted in isolation but rather integrated with the clinical history and, whenever necessary, confirmed by OFC. Incorrect interpretation may lead to overdiagnosis, unnecessary elimination diets, nutritional deficiencies, and increased parental anxiety [25].

Several observational studies consistently support the added value of specialist allergy consultation. Miceli Sopo et al. emphasized that specialist assessment facilitates appropriate diagnostic testing, confirms the diagnosis through objective investigations, and enables individualized management strategies, including targeted elimination diets and emergency action plans when indicated [26]. Similarly, Caimmi et al. [27] highlighted the complementary roles of primary care pediatricians and allergists, showing that while initial management can safely begin in primary care, children with suspected IgE- and non-IgE-mediated food allergy should be referred for specialist evaluation to undergo SPT, serum-specific IgE testing, and OFC, thereby avoiding unnecessary dietary restrictions and improving clinical and psychosocial outcomes. Comparable conclusions were reported by Calvani et al. [28], who proposed a structured diagnostic pathway integrating clinical history, allergy testing, elimination diets, and OFC in selected cases to achieve an accurate diagnosis and appropriate therapeutic management.

Overall, the available evidence consistently indicates that allergy consultation provides substantial clinical benefits over follow-up by the primary care pediatrician alone in children with suspected food allergy and atopic dermatitis. Specialist assessment improves diagnostic precision, supports personalized management, minimizes unnecessary dietary restrictions, and may contribute to better health-related quality of life for both children and their families.

Recommendation (Table 2). Given the low certainty of the available evidence and the predominantly observational nature of the supporting studies, a conditional recommendation in favour of specialist referral was formulated. Briefly, children and adolescents with suspected food allergy and atopic dermatitis should be referred to an allergy specialist to ensure an accurate diagnosis, reduce false positives, and initiate specific therapy with potential improvement in quality of life.

##### PICO 2

In children with suspected food allergy, is the use of the OFC more effective than diagnosis based solely on skin prick tests and specific IgE measurements in reducing overdiagnosis and ensuring an adequate diagnosis of food allergy?

Narrative report. The diagnosis of IgE-mediated food allergy in children remains challenging and requires the integration of clinical history with appropriate diagnostic testing. According to the SIGENP–SIAIP diagnostic and therapeutic pathway, the OFC remains essential to confirm or exclude the diagnosis of food allergy. Reliance on sensitization tests alone, including SPT and serum-specific IgE measurements, may lead to overdiagnosis because these investigations demonstrate sensitization rather than clinically relevant allergy. Consequently, OFC improves diagnostic appropriateness, avoids unnecessary elimination diets, and prevents unjustified dietary restrictions [29].

Similarly, the EAACI guidelines recognize that SPT and serum-specific IgE are highly sensitive but have limited specificity. Although both tests represent valuable first-line diagnostic tools, they should not be used in isolation to establish the diagnosis of food allergy. Accordingly, the guidelines recommend oral food challenge—preferably double-blind, placebo-controlled whenever feasible—as the diagnostic gold standard, particularly in patients with discordant test results or an uncertain clinical history [2].

The available observational evidence consistently supports these recommendations. A five-year retrospective study conducted in a pediatric allergy center demonstrated that OFC frequently refuted presumed food allergy diagnoses based solely on sensitization tests, substantially reducing unnecessary exclusion diets. Among 456 oral food challenges performed, only 56 (12.3%) were positive, confirming clinically relevant food allergy [30].

Comparable findings were reported by other studies, showing that OFC often disproves diagnoses based exclusively on sensitization testing, particularly when fresh food is used for the challenge while commercial extracts are employed for SPT [25,31]. Likewise, Diaz et al. demonstrated that neither SPT nor serum-specific IgE alone provides sufficient diagnostic accuracy for cow’s milk protein allergy. Although the casein SPT showed high specificity (96.7%), its sensitivity remained limited, whereas the highest diagnostic performance was achieved by combining SPT and serum-specific IgE testing. Nevertheless, OFC remained necessary to establish or exclude the diagnosis and to optimize nutritional management [32].

Finally, a systematic review and meta-analysis confirmed that SPT and serum-specific IgE are appropriate screening tools for allergic sensitization but have limited ability to confirm clinical food allergy. Across the available evidence, oral food challenge remains the only diagnostic test with sufficient specificity to reliably confirm food allergy and is therefore regarded as the current diagnostic gold standard [33,34].

Overall, the available evidence consistently indicates that sensitization tests should be interpreted as complementary diagnostic tools, whereas oral food challenge remains indispensable whenever confirmation of clinically relevant food allergy is required.

Recommendation (Table 3). In children with suspected food allergy, OFC is strongly recommended, when clinically indicated and feasible, to confirm the diagnosis and avoid unnecessary dietary restrictions. Diagnosis based solely on SPT and specific IgE is not recommended because of the risk of overdiagnosis.

##### PICO 3

In children and adolescents with suspected eosinophilic gastrointestinal disease or non-IgE-mediated food allergy, is a specialist gastroenterology/allergy consultation recommended compared to independent management by the primary care paediatrician for achieving an accurate diagnosis and initiating specific treatment?

Narrative report. Eosinophilic gastrointestinal disorders (EGIDs) and non-IgE-mediated food allergies comprise a heterogeneous group of chronic inflammatory diseases characterized by abnormal eosinophilic infiltration of the gastrointestinal tract. Eosinophilic esophagitis (EoE) is the most extensively studied condition, whereas eosinophilic gastritis, enteritis, and colitis are less common and often present with nonspecific clinical manifestations, making diagnosis particularly challenging in children [3,35,36].

Primary care pediatricians play a key role in the early recognition of suspected cases. However, confirmation of the diagnosis and appropriate therapeutic management generally require referral to pediatric gastroenterology and allergy specialists within a multidisciplinary setting.

Current international guidelines consistently identify upper gastrointestinal endoscopy with multiple biopsies as the cornerstone of diagnosis. According to the American College of Gastroenterology (ACG), the diagnosis of EoE requires both symptoms of esophageal dysfunction (e.g., dysphagia, food impaction, regurgitation, vomiting, chest pain, or poor growth) and histological evidence of at least 15 eosinophils per high-power field in esophageal biopsy specimens [36]. Similarly, the 2024 ESPGHAN recommendations advocate systematic upper gastrointestinal endoscopy with multilevel biopsy sampling, recommending at least six biopsies obtained from different esophageal levels to maximize diagnostic yield [3,35].

For non-esophageal EGIDs, the joint ESPGHAN/NASPGHAN recommendations require three essential diagnostic criteria: (1) compatible gastrointestinal symptoms; (2) dense eosinophilic infiltration demonstrated on mucosal biopsy; and (3) exclusion of alternative causes of gastrointestinal eosinophilia, including parasitic infections, inflammatory bowel disease, drug-related disorders, neoplasms, and other allergic conditions. Histological thresholds vary according to the anatomical segment involved and should therefore be interpreted in the appropriate clinical context [35].

The available evidence consistently highlights the pivotal role of endoscopic and histological evaluation in establishing the diagnosis. In a recent systematic review, Calvani et al. reported that endoscopy with biopsy provides greater diagnostic accuracy than oral food challenge in patients with persistent gastrointestinal symptoms suggestive of non-IgE-mediated or mixed food allergy. Interestingly, although histological relapse occurred in all patients following food reintroduction after an elimination diet, only 42% experienced recurrence of clinical symptoms, emphasizing that histological activity and symptom severity do not necessarily correlate and should be interpreted together [37].

These findings support current Italian recommendations issued by SIGENP and SIAIP, which advocate a structured multidisciplinary diagnostic pathway. While the initial clinical assessment should be performed by the primary care pediatrician, children with suspected non-IgE-mediated food allergy or EGIDs should be referred to secondary or tertiary referral centers, where pediatric allergists, gastroenterologists, endoscopists, pathologists, and dietitians collaborate in diagnostic evaluation and therapeutic planning [29].

Overall, the available evidence consistently indicates that specialist multidisciplinary assessment, including upper gastrointestinal endoscopy with appropriate biopsy sampling, is essential for achieving an accurate diagnosis, excluding alternative causes of gastrointestinal eosinophilia, guiding individualized treatment, and minimizing the risk of delayed diagnosis and long-term consequences on growth and development [19].

Recommendation. Due to the moderate quality of evidence, a consensus-based rather than evidence-based recommendation was framed (Table 4). Referral to a specialist gastroenterologist/allergist is recommended for children and adolescents with suspected eosinophilic gastrointestinal disease or non-IgE-mediated food allergy, in order to undertake an appropriate diagnostic pathway, including endoscopic examination with biopsies, so that specific treatment can be initiated.

##### PICO 4

In children and adolescents with suspected eosinophilic gastrointestinal disease, is endoscopy with biopsy more strongly recommended than serological and skin tests alone for confirming the diagnosis and guiding therapy?

Narrative report. The diagnosis of eosinophilic EGIDs, including EoE and non-oesophageal EGIDs, relies on endoscopic evaluation with histological confirmation. Neither serum food-specific IgE nor SPT should be used alone to establish or exclude the diagnosis because both demonstrate sensitization rather than tissue eosinophilic inflammation and have limited negative predictive value [38].

The available evidence consistently shows that conventional allergy tests have limited diagnostic performance in children with EGIDs. In a retrospective cross-sectional study including 35 children with confirmed or suspected EoE, SPT was positive in only 75% of cases, whereas serum-specific IgE was detected in just 50% of patients, confirming the relatively low prevalence of IgE-mediated food allergy in this population [39]. Similar findings were reported in an observational study of 108 children with EoE, in which SPT and serum-specific IgE were positive in only 60.6% and 54.4% of patients, respectively [38]. Likewise, an earlier pediatric study reported positive SPT results in only 5.9% of children with EoE, although serum-specific IgE showed a somewhat higher positivity rate [40].

Further evidence indicates that neither SPT nor serum-specific IgE adequately predicts oesophageal eosinophilia. In a retrospective cohort of 144 children, the probability of EoE increased with the number of positive food-specific IgE; however, approximately 15% of patients had no detectable sensitization, limiting the predictive performance of serological testing alone [23]. Similar observations have also been reported for non-oesophageal EGIDs. A Korean pediatric study found positive serum-specific IgE in only half of the investigated patients, while an Italian retrospective study confirmed the strong association between EGIDs and atopy but also demonstrated considerable heterogeneity in allergological findings, particularly between EoE and non-oesophageal EGIDs [41,42].

The atopy patch test (APT) may provide additional diagnostic information in selected patients with suspected non-IgE-mediated food allergy. A recent systematic review including 16 studies demonstrated that APT has high specificity (up to 96%), particularly in food protein-induced motility disorders, but poor sensitivity. Consequently, a positive APT strongly supports the diagnosis, whereas a negative result cannot reliably exclude disease [43].

Overall, the available evidence consistently indicates that histological confirmation obtained through endoscopic biopsy remains indispensable for the diagnosis of EGIDs. Allergy tests should therefore be regarded as complementary investigations that contribute to characterizing the patient’s atopic profile rather than confirming the diagnosis. Histological assessment also allows exclusion of alternative causes of gastrointestinal eosinophilia, including parasitic infections, autoimmune disorders, inflammatory bowel disease, and neoplastic conditions, while providing the basis for individualized therapeutic strategies, including elimination diets, anti-inflammatory or immunomodulatory treatment, and long-term monitoring of both clinical and histological response [44].

Recommendation (Table 5). The exclusive use of serum-specific IgE, SPT, or APT is not recommended in children and adolescents with suspected EGIDs, as these tests characterize allergic sensitization but cannot reliably confirm or exclude tissue eosinophilic inflammation. Endoscopic evaluation with appropriate gastrointestinal biopsies and histological assessment remains the diagnostic gold standard. A definitive diagnosis is essential to guide individualized treatment and long-term follow-up.

### 3.2. Urticaria and Angioedema

#### 3.2.1. Summary of Literature Search

**Figure 2:**
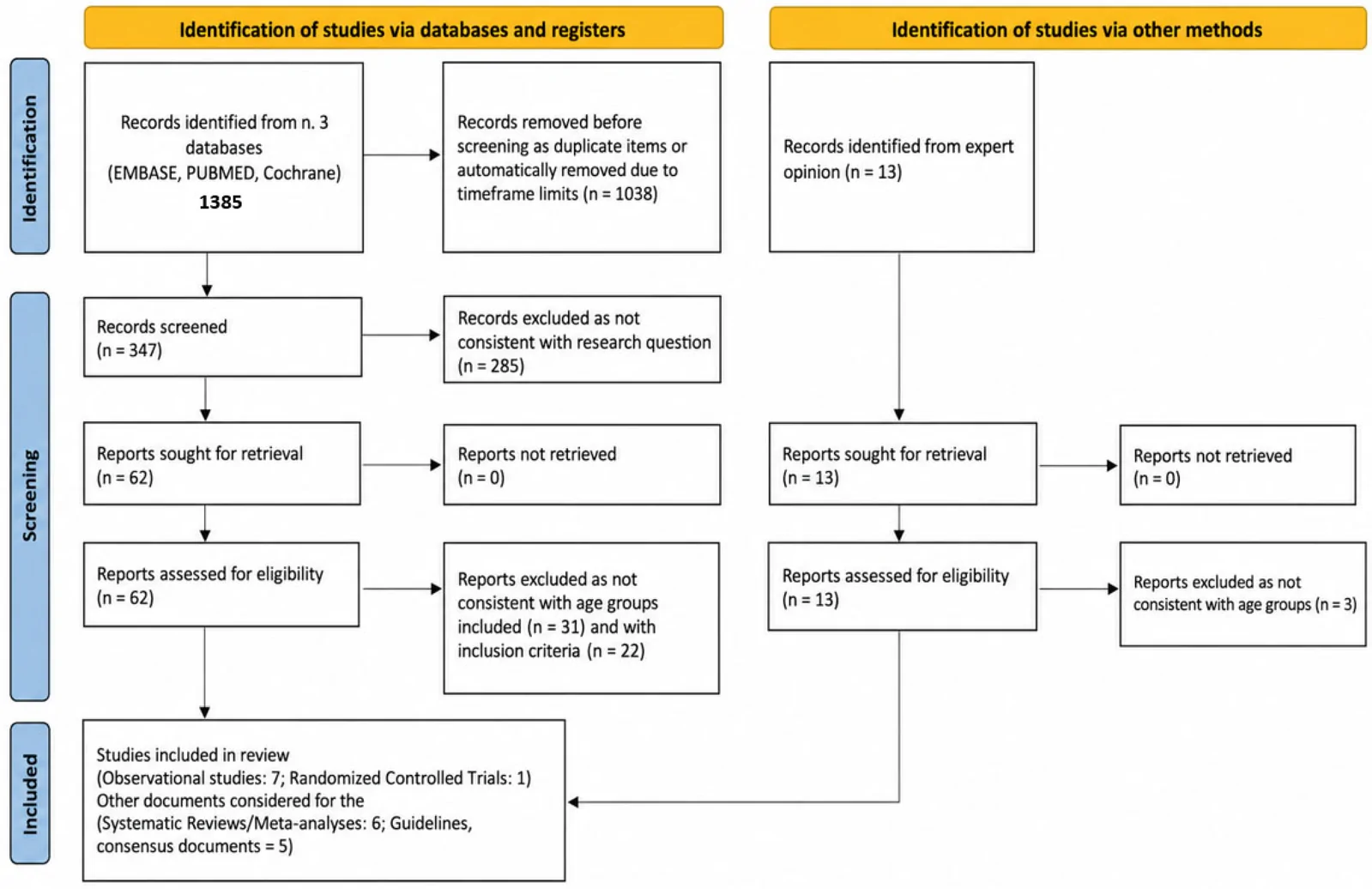
Flow-chart of included studies on urticaria and angioedema. A total of 19 documents were included, comprising 9 records identified through database searches and 10 identified through expert consultation. Irrespective of their source of identification, the final evidence base comprised 7 observational studies, 1 randomized controlled trial, 6 systematic reviews/meta-analyses, and 5 guidelines or consensus documents.

A search of three databases (Embase, Medline, Cochrane) identified 1385 articles. After removing duplicates (*n* = 347), 62 studies were screened by title and abstract (Figure 2).

Of these, 23 articles were reviewed in full and 9 were included in the final analysis. Based on expert opinion, a further 10 articles were added, for a total of 19 articles included. Seven studies were observational, 1 was a RCT; the remainder were meta-analyses and guidelines/recommendations. Study selection process is presented in the PRISMA flow diagram reported in Figure 2. The studies included in the search are reported and described in Table A2, Table A3 and Table A4. Quality appraisal of included studies is summarised in Table A5, Table A6, Table A7 and Table A8.

The outcomes considered, depending on the PICO. question, mainly concerned:

- the diagnostic accuracy of allergological tests in identifying the causal agent and initiating specific therapy
- quality of life measured using standardised questionnaires

#### 3.2.2. Clinical Questions

The full list of inquired PICOs is provided by Table 6.

##### PICO 1

In children and adolescents with chronic urticaria, is an allergological consultation recommended over standard follow-up by the general paediatrician in order to initiate specific therapy and improve quality of life?

Narrative report. Chronic spontaneous urticaria (CSU) in children is frequently underdiagnosed and under-referred, despite its substantial impact on quality of life. Although primary care pediatricians play a key role in the initial recognition and management of CSU, referral to an allergy or dermatology specialist should be considered in children with symptoms that remain uncontrolled despite standard-dose second-generation antihistamines, in patients with chronic or atypical disease, and whenever the diagnosis remains uncertain or potential triggers cannot be identified. Specialist evaluation is also required because third- and fourth-line treatment options recommended by current international guidelines, including biologic therapies, are generally available only in tertiary referral centers and are not approved for all pediatric age groups [4,45].

Specialist assessment enables confirmation of the diagnosis, exclusion of alternative causes of urticaria, optimization of pharmacological treatment, and avoidance of unnecessary diagnostic investigations. For patients with antihistamine-refractory CSU, increasing evidence supports the efficacy and safety of omalizumab, while other immunomodulatory therapies, such as ciclosporin, should be reserved for selected severe cases and prescribed only under specialist supervision [46,47].

Beyond symptom control, specialist management has consistently been associated with significant improvements in health-related quality of life. Several real-world observational studies have shown that treatment with omalizumab substantially reduces disease activity while improving validated quality-of-life measures. In a pediatric cohort, the Children’s Dermatology Life Quality Index (CDLQI) decreased from 17.5 to 2.0 after 16 weeks of treatment, indicating a marked improvement in patient well-being [48]. Similar benefits were observed in larger retrospective and observational studies, in which omalizumab significantly improved Urticaria Activity Score (UAS/UAS7), Urticaria Control Test (UCT), and quality-of-life scores after three to twelve months of treatment [49,50].

These findings are consistent with evidence from randomized clinical trials. In a post hoc analysis of the phase III ASTERIA I, ASTERIA II, and GLACIAL trials, Finlay et al. demonstrated that omalizumab 300 mg significantly improved Dermatology Life Quality Index (DLQI) scores compared with placebo after 12 weeks of treatment [51].

Overall, the available evidence consistently indicates that specialist follow-up plays a pivotal role in the management of pediatric CSU. Access to specialist care facilitates appropriate use of advanced therapies, improves disease control, minimizes unnecessary investigations, and results in clinically meaningful improvements in health-related quality of life. Given the profound psychosocial impact of chronic urticaria during childhood and adolescence, early referral to specialist services should be considered an integral component of optimal patient management [52].

Recommendation (Table 7). Children and adolescents with CSU should be referred to an allergy specialist when symptoms persist despite first-line therapy, the diagnosis is uncertain, or advanced treatment is required. Specialist assessment facilitates early diagnosis, optimization of therapy, access to targeted treatments when indicated, and contributes to improved disease control and health-related quality of life.

##### PICO 2

In children and adolescents with chronic urticaria, does the routine performance of allergological tests (skin prick test, specific IgE) compared to management based solely on clinical history improve the identification of triggering causes?

Narrative report. CSU in children is predominantly idiopathic, and an underlying allergic cause can be identified only in a minority of patients. Consequently, routine allergy testing has limited diagnostic value and should not be performed indiscriminately.

Available observational evidence consistently supports this approach. In a cohort of 222 children with CSU, elevated total IgE levels (>100 U/L) and positive SPT were observed in 37.8% and 27.9% of patients, respectively; however, sensitization proved clinically relevant in only a small proportion of cases. Despite comprehensive clinical evaluation and extensive laboratory investigations, a definite underlying cause was identified in only a minority of patients [53]. Similar findings were reported in another observational study of 37 children with chronic urticaria, in which 86.5% of cases were ultimately classified as spontaneous despite allergological investigations. The authors concluded that SPT and serum-specific IgE should be reserved for patients in whom the clinical history raises a genuine suspicion of an allergic trigger [54].

These findings are supported by systematic reviews and international guidelines. A recent review estimated that allergy accounts for fewer than 10% of pediatric CSU cases, while food allergy is confirmed in only 2.8–4.0% of patients when oral food challenge is used as the reference standard [45]. Likewise, Caffarelli et al. found no convincing evidence that foods, inhalant allergens, or drugs represent common causes of pediatric CSU, recommending allergy testing only when a clear temporal relationship exists between allergen exposure and symptom onset [55,56].

Current international guidelines consistently discourage routine allergological investigations in children with CSU. Both the EAACI/GA^2^LEN/EuroGuiDerm/APAAACI recommendations and the British Society for Allergy & Clinical Immunology (BSACI) guidelines emphasize that a careful medical history and physical examination are generally sufficient to establish the diagnosis and guide further investigations. Allergy tests, including SPT and serum-specific IgE, should therefore be performed only when supported by the clinical history, thereby reducing unnecessary investigations and minimizing the risk of false-positive results and inappropriate clinical management [4,47].

Recommendation (Table 8). Routine allergological investigations are not recommended in children and adolescents with CSU. Diagnostic evaluation should be guided by a detailed clinical history and physical examination, with skin prick tests and serum-specific IgE reserved for patients in whom the clinical history raises suspicion of an underlying allergic cause.

##### PICO 3

In children and adolescents with chronic urticaria, is a targeted diagnostic approach based on clinical history, compared to an extensive diagnostic work-up with non-targeted allergological and autoimmune tests, more effective in improving diagnostic appropriateness, including reducing costs, increasing correct diagnoses, and reducing unnecessary tests?

Evidence. Increasing evidence suggests that children with CSU have a higher prevalence of autoimmune diseases than the general pediatric population, supporting the concept that autoimmune mechanisms contribute to the pathogenesis of CSU in a substantial proportion of patients [53,54,57,58,59].

Observational studies have consistently documented an increased frequency of autoimmune disorders among children with CSU. In a cohort including 23 pediatric patients, Barzilai et al. reported autoimmune diseases in 34.8% of cases, including autoimmune liver disease, inflammatory bowel disease, and hypothyroidism [58]. Similar findings have been described in other pediatric cohorts, showing increased frequencies of autoimmune thyroid disease, celiac disease, and type 1 diabetes compared with the general population [54,57,59]. Although thyroid autoantibodies and other markers of autoimmunity are frequently detected, progression to clinically overt autoimmune disease appears considerably less common [57].

Several studies have also investigated laboratory markers potentially associated with autoimmune CSU. Elevated anti-thyroid peroxidase (anti-TPO) antibodies, positive antinuclear antibodies (ANA), and positive autologous serum skin tests (ASST) have all been reported in subsets of pediatric patients [53,57]. Nevertheless, these findings should be interpreted cautiously, as their clinical significance remains variable and routine screening for all available autoimmune markers is not currently supported by the available evidence. Likewise, skin prick tests and serum-specific IgE frequently identify sensitization without demonstrating a causal relationship with urticaria, reinforcing that allergological investigations should be guided by the clinical history rather than performed routinely [53].

Current evidence therefore supports a targeted diagnostic approach. Autoimmune investigations should be considered primarily in children with persistent or antihistamine-refractory CSU, those with systemic manifestations suggestive of autoimmune disease, or patients with a personal or family history of autoimmunity. Suggested investigations may include thyroid function tests (TSH, free T4, anti-TPO, anti-thyroglobulin antibodies), screening for celiac disease (anti-tissue transglutaminase and anti-endomysial antibodies), and, when clinically indicated, ANA testing, ASST, basophil activation test (BAT), or evaluation for type 1 diabetes [54,55,56,57,58,59,60].

This approach is consistent with current international guidelines. The EAACI/GA^2^LEN/EuroGuiDerm/APAAACI recommendations advocate a careful clinical history and physical examination as the cornerstone of diagnostic assessment, complemented by measurement of total IgE and anti-TPO antibodies. Additional laboratory investigations should be tailored to the individual clinical presentation. Similarly, the BSACI guidelines recommend screening for celiac disease and thyroid dysfunction when clinically indicated, whereas ANA testing should be reserved for patients in whom connective tissue disease is suspected [4,47].

Overall, the available evidence supports a selective, history-driven diagnostic strategy rather than extensive routine laboratory screening. Such an approach may facilitate the identification of clinically relevant autoimmune comorbidities while minimizing unnecessary investigations and improving the appropriateness of diagnostic testing.

Recommendation (Table 9). In children and adolescents with CSU, a targeted diagnostic approach is conditionally recommended. Evaluation for thyroid dysfunction and coeliac disease should be considered, while investigation for other autoimmune disorders should be guided by clinical suspicion, including suggestive symptoms, a personal or family history of autoimmunity, or refractory disease. Similarly, allergological investigations should be reserved for patients with a compatible clinical history, thereby improving diagnostic accuracy while minimizing unnecessary investigations and resource utilization.

##### PICO 4

In children and adolescents with recurrent episodes of angioedema without urticaria, is a specialist allergological and/or immunological consultation recommended compared to management by the primary care paediatrician alone, in order to identify possible hereditary or acquired forms and improve disease management?

Narrative report. Recurrent angioedema in children requires careful differential diagnosis because its underlying mechanisms, prognosis, and treatment vary substantially. Current WAO/EAACI guidelines emphasize the importance of distinguishing histamine-mediated angioedema, which is frequently associated with chronic spontaneous urticaria and generally responds to antihistamines, from bradykinin-mediated forms, particularly hereditary angioedema (HAE), which are characterized by slower onset, prolonged duration, absence of urticaria, and lack of response to antihistamines, corticosteroids, or adrenaline [5].

A thorough personal and family history, comprehensive physical examination, and targeted laboratory investigations are therefore essential to establish the correct diagnosis. In children presenting with recurrent angioedema of unknown origin, particularly when associated with recurrent abdominal pain, upper airway involvement, a positive family history, or absence of wheals, further specialist investigations should be undertaken to exclude hereditary forms of angioedema [5,61,62].

Hereditary angioedema is a rare but potentially life-threatening disease that frequently begins during childhood. Because its initial manifestations are often nonspecific, considerable diagnostic delay remains common. In addition to limited clinical awareness, barriers to appropriate diagnosis and treatment may include restricted access to specialist services and disease-specific therapies. Consequently, early referral to a specialist center plays a pivotal role in confirming the diagnosis, establishing appropriate follow-up, and initiating individualized management [5].

This approach is consistently supported by international recommendations. Patients presenting to the emergency department with angioedema should undergo specialist evaluation after resolution of the acute episode, irrespective of whether an allergic or hereditary mechanism is initially suspected [63]. Likewise, international consensus recommendations advocate lifelong follow-up of children with hereditary angioedema in specialized referral centers, with at least annual clinical assessment. Such follow-up allows monitoring of growth and development, optimization of disease-specific therapy, patient and family education, and close collaboration between specialist services and primary care [61].

Overall, the available evidence consistently indicates that specialist assessment is essential whenever hereditary angioedema is suspected. Accurate differential diagnosis between histamine- and bradykinin-mediated angioedema enables appropriate therapeutic management, reduces the frequency and severity of attacks, prevents potentially life-threatening complications, and ensures continuity of care throughout childhood and adolescence [5,61,62].

Recommendation (Table 10). In children and adolescents with angioedema, referral to an allergology specialist is recommended rather than routine paediatric follow-up alone, in order to ensure an accurate differential diagnosis, initiate appropriate monitoring, and establish a personalised treatment plan.

##### PICO 5

In children and adolescents with isolated recurrent angioedema without urticaria and without obvious causes, is the performance of specific laboratory tests (C1 inhibitor, C4, and C1q measurement) more appropriate than clinical observation alone for differentiating allergic forms from hereditary or acquired C1 inhibitor deficiency?

Narrative report. In children with recurrent episodes of angioedema of unknown aetiology, it is recommended to measure both the functional and antigenic levels of C1 inhibitor (C1-INH), together with C4, to assess the possible presence of hereditary forms. A reduced functional level of C1-INH accompanied by a low C4 is indicative of hereditary angioedema due to C1 inhibitor deficiency (C1-INH-HAE) at any age, although diagnostic confirmation is required. If this finding is associated with a reduction in the antigenic level of C1-INH, it is most likely C1-INH-HAE type I. Conversely, if C4 and functional C1-INH are low but the antigenic level of C1-INH is normal or elevated, C1-INH-HAE type II should be considered. It is essential to repeat the tests to confirm the diagnosis. Acquired angioedema due to C1-INH deficiency, mediated by antibodies, C1-INH consumption, or associated with B-cell disorders, typically manifests in adulthood and is rare before the age of 40; therefore, routine measurement of C1q is not recommended in the paediatric population [61].

The international WAO/EAACI guidelines emphasise the importance of an accurate differential diagnosis in patients with a history of recurrent oedema, including distinguishing between hereditary angioedema (HAE), HAE with normal C1-INH, acquired bradykinin-mediated angioedema, and mast cell-mediated forms such as angioedema associated with chronic spontaneous urticaria without wheals, allergic angioedema, and idiopathic acquired angioedema. A comprehensive diagnostic evaluation is therefore crucial. In suspected allergic forms, the measurement of specific IgE may be indicated. HAE type I and II (HAE-1/2) should be suspected in individuals with recurrent cutaneous swelling (for example, affecting the limbs, face, or genitals), gastrointestinal attacks (e.g., severe abdominal pain), or episodes of laryngeal oedema. The likelihood increases in the presence of at least one of the following elements: (1) positive family history (although up to 25% of patients have no family history); (2) onset of symptoms in childhood or adolescence; (3) recurrent abdominal pain; (4) involvement of the upper airways; (5) lack of response to antihistamines, corticosteroids, omalizumab, or adrenaline; (6) presence of prodromal symptoms; (7) absence of urticarial wheals. In the case of suspected HAE-1/2, laboratory assessment should include measurement of the functional activity of C1-INH, antigenic levels of C1-INH, and C4. The combined interpretation of these three parameters improves diagnostic accuracy. Abnormal results must be confirmed.

It should be noted that C4 levels are physiologically low in healthy children under 12 months of age, which limits their usefulness in this age group. If both the functional and antigenic levels of C1-INH are normal but clinical suspicion of HAE persists, genetic testing for pathogenic mutations is indicated. Newborns with a positive family history of C1-INH-HAE should be considered potentially affected and monitored appropriately. Diagnostic testing should be performed as soon as possible, ideally before the onset of symptoms, to ensure timely diagnosis and optimal management; this test should be repeated after the first year of life to confirm the diagnosis. In suspected cases of acquired angioedema due to C1-INH deficiency, C1q measurement is recommended to differentiate hereditary from acquired forms, especially if symptoms begin after 30 years of age and in the absence of a family history [5]. Brazilian guidelines similarly recommend that individuals with angioedema, particularly those with a positive family history, undergo appropriate diagnostic investigation. C4 represents a useful initial screening test, as it is usually reduced in patients with HAE. However, C4 may normalise during asymptomatic periods in 2–5% of cases, so a normal level does not exclude HAE. C3 measurement is not indicated in this context. The assessment of C1-INH should include both quantity and functionality, together with the C4 test. If clinical suspicion persists despite normal laboratory values, repeating the tests during an acute episode may improve diagnostic yield. If C4 and C1-INH (functional and antigenic) are repeatedly normal, HAE with normal C1-INH (previously type III) should be considered, for which genetic testing is indicated to identify underlying mutations [64].

The BSACI recommends that in children—typically adolescents—with angioedema without urticaria, serum measurements of C4 and quantitative C1-INH should be performed to assess for possible C1-INH deficiency [47]. The laboratory plays a crucial role in distinguishing the types of angioedema. C4 levels are usually reduced during HAE attacks and serve as an initial screening marker. Elevated tryptase levels indicate anaphylaxis, whereas normal levels are consistent with HAE. For a definitive diagnosis of HAE, it is essential to measure C4, antigenic C1-INH, and the functional activity of C1-INH, confirming any abnormalities with repeat testing. A persistent deficiency in both the quantity and function of C1-INH confirms the diagnosis of HAE. C1q levels are generally normal in hereditary forms; a reduction in C1q may indicate acquired angioedema, especially when onset occurs after the age of 40. Timely and accurate diagnosis is fundamental to guide appropriate management and prevent potentially life-threatening complications [63,65].

Recommendation (Table 11). In children and adolescents with recurrent angioedema of unknown cause, it is recommended to perform targeted laboratory investigations—such as quantitative and functional measurement of C1 inhibitor, C4, and C1q—based on the clinical history, in order to facilitate the distinction between different forms of angioedema.

### 3.3. Atopic Dermatitis

#### 3.3.1. Summary of Literature Research

**Figure 3:**
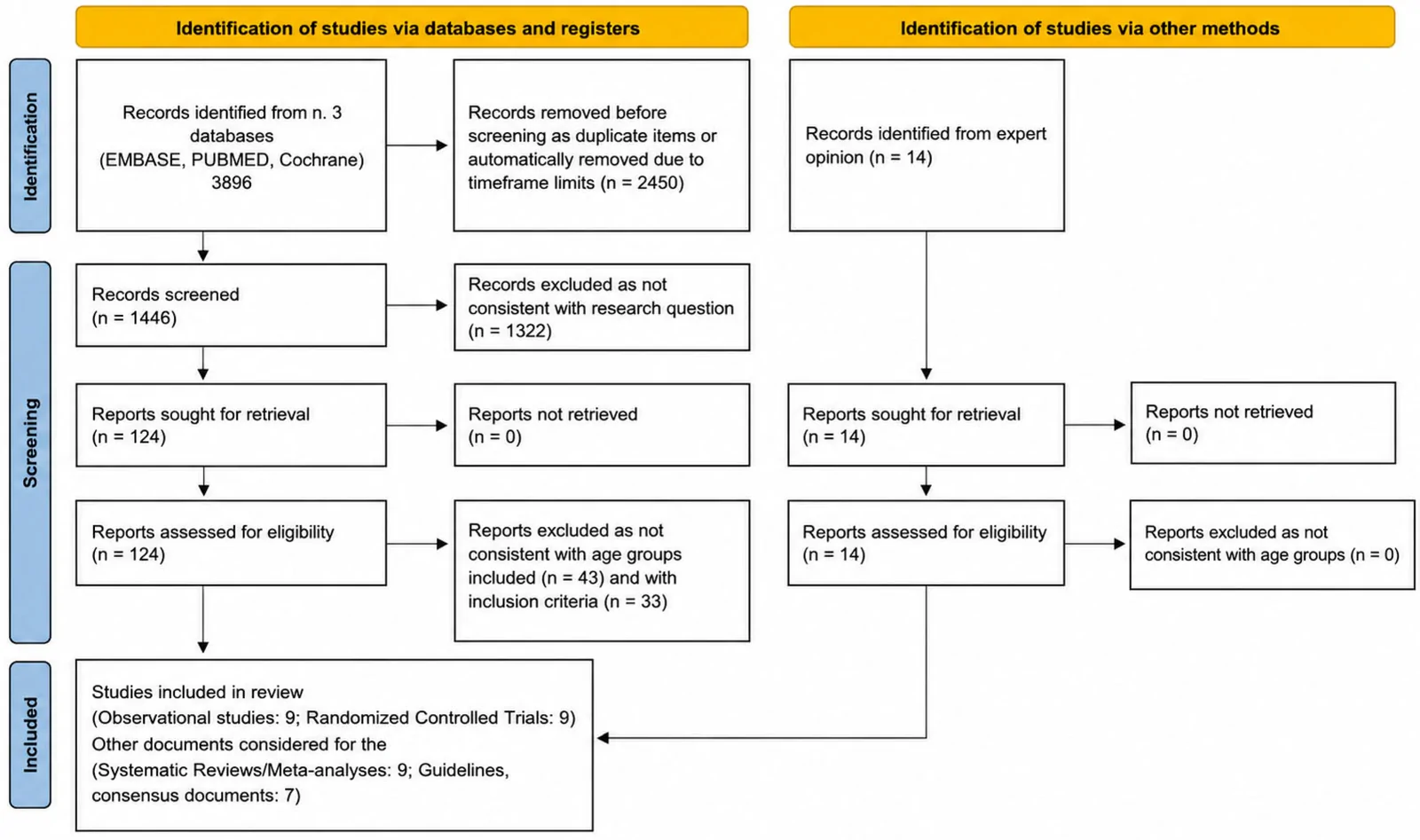
Flow chart of studies included in the analyses on atopic dermatitis. A total of 34 documents were included in the final evidence synthesis, comprising 9 observational studies, 9 randomized controlled trials, 9 systematic reviews/meta-analyses, and 7 guidelines or consensus documents. Irrespective of their source of identification, documents were classified in the final box according to study/document type.

The search on three databases (Embase, Medline, Cochrane) identified a total of 3896 articles. After removing duplicates (*n* = 1446), 124 studies were screened by title and abstract (Figure 3). Of these, 37 articles were fully reviewed and 20 were included in the final analysis. Based on expert opinion, 14 additional articles were added, for a total of 34 articles included. Nine studies were observational studies, 9 were RCT; the remaining were meta-analyses and guidelines/recommendations.

The studies included in the search are reported and described in Table A2, Table A3 and Table A4. Quality appraisal of included studies is summarised in Table A5, Table A6, Table A7 and Table A8. The outcomes considered, depending on the PICO framework, mainly included:

- Diagnostic accuracy through allergy tests to identify the causative agent and initiate specific therapy
- Quality of life of the patient and family measured through standardized questionnaires
- Risk of exacerbations

#### 3.3.2. Clinical Questions

The full list of inquired PICOs is provided by Table 12.

##### PICO 1

In children and adolescents with severe atopic dermatitis (AD), is allergy consultation recommended over routine follow-up with a general pediatrician to initiate specific therapy and improve quality of life?

Narrative report. Moderate-to-severe AD in children is associated with a substantial disease burden, frequent allergic comorbidities (including asthma, allergic rhinitis, food allergy, and eosinophilic esophagitis), and impaired quality of life (QoL) affecting both patients and their families. A systematic review and meta-analysis showed that, although AD resolves in most children by the age of 8 years, persistent, late-onset, or severe forms are associated with a significantly increased risk of chronic disease in adulthood [66]. Likewise, data from the CHILD cohort demonstrated that children with AD and allergic sensitization have a markedly increased risk of developing asthma (aRR = 7.04) and food allergy (aRR = 33.79) compared with non-sensitized children [67]. These findings identify a subgroup of patients who may particularly benefit from specialist evaluation.

Current international consensus guidelines consistently recommend specialist allergy assessment in children and adolescents with moderate-to-severe AD, particularly when disease control is suboptimal, allergic comorbidities are suspected, or advanced systemic therapy is being considered [68,69,70,71]. The rationale for referral includes: (1) the high prevalence of coexisting atopic diseases, especially in severe or early-onset AD [70]; (2) the need to distinguish clinically relevant sensitizations from asymptomatic sensitization in order to avoid unnecessary dietary or environmental restrictions [69]; (3) access to multidisciplinary management for patients with persistent symptoms or inadequate response to topical therapy [71] and (4) evaluation for advanced systemic therapies, including biologic agents [68].

Specialist assessment is particularly important when systemic treatment is indicated. Recent reviews and international guidelines identify dupilumab as the cornerstone biologic therapy for pediatric patients with moderate-to-severe AD. Currently approved from 6 months of age, dupilumab requires careful disease severity assessment, evaluation of associated allergic comorbidities, and long-term monitoring, all of which are generally provided in specialized allergy or dermatology centers [68,69,70,72]. Similarly, calcineurin inhibitors, such as tacrolimus, are recommended as steroid-sparing agents for sensitive anatomical sites, while emerging therapies—including Janus kinase (JAK) inhibitors and novel biologics—are expected to further reinforce the role of specialist referral in the management of severe AD [68,69].

Current guidelines also discourage routine allergy testing in the absence of clinical features suggestive of immediate hypersensitivity. Instead, allergological investigations should be guided by the medical history and clinical presentation, thereby improving diagnostic appropriateness while minimizing unnecessary investigations. Early referral to a specialist facilitates disease severity stratification, identification of relevant comorbidities, and timely therapeutic escalation when indicated. Despite the limited number of randomized controlled trials directly addressing referral strategies, current evidence consistently supports specialist evaluation in children with moderate-to-severe AD [73].

Structured therapeutic education represents another fundamental component of specialist management. Randomized and prospective studies have consistently demonstrated that multidisciplinary educational interventions improve disease severity, treatment adherence, and quality of life for both children and their caregivers [74,75,76]. These findings have subsequently been confirmed by two meta-analyses showing significant reductions in disease severity, commonly measured by SCORAD (SCORing Atopic Dermatitis) or POEM (Patient-Oriented Eczema Measure), together with improvements in QoL assessed using validated instruments such as the Infants’ Dermatitis Quality of Life Index (IDQOL) and the Children’s Dermatology Life Quality Index (CDLQI) [77,78].

Educational interventions evaluated in clinical studies include the German “eczema school” model, consisting of six weekly multidisciplinary sessions for children and their families [79], structured educational workshops focused on skin care and emollient use [80], post-consultation counselling, written educational materials, and more recently, digital educational platforms such as online videos and smartphone applications [74,81,82]. Collectively, these studies demonstrate that both face-to-face and remote educational programmes improve disease control, treatment adherence, and family empowerment when appropriately structured and tailored to patients’ needs.

Pustišek et al. [74] demonstrated that a structured multidisciplinary educational programme significantly reduced SCORAD scores, pruritus, sleep disturbances, and parental anxiety in children with moderate-to-severe AD. Similarly, Salava et al. [75] reported substantial improvements in QoL during long-term follow-up irrespective of the topical treatment used, whereas Gazibara et al. [76] showed that reductions in SCORAD scores were strongly associated with improvements in QoL. However, psychosocial impairment and sleep disturbances occasionally persisted despite clinical improvement, supporting the need for comprehensive multidisciplinary management.

Recent randomized clinical trials and real-world studies further demonstrate that dupilumab significantly improves QoL in children with moderate-to-severe AD. Clinically meaningful improvements have been documented using several validated outcome measures, including POEM, CDLQI, IDQOL, and DLQI, with benefits becoming evident within 2–4 weeks and persisting throughout long-term follow-up. In the LIBERTY AD PEDS trial, more than 77% of treated children achieved the minimal clinically important difference in CDLQI or POEM scores by week 16 [83]. These findings have subsequently been confirmed by extension studies and real-world cohorts, demonstrating significant reductions in pruritus, sleep disturbances, psychological burden, and overall impairment of family well-being [84,85,86,87,88].

Overall, the available evidence consistently supports early referral of children with moderate-to-severe AD to specialized allergy services. Specialist assessment facilitates identification of allergic comorbidities, appropriate interpretation of allergological investigations, implementation of structured educational programmes, timely access to advanced therapies, and long-term multidisciplinary follow-up, ultimately improving disease control and quality of life for both patients and their families.

Recommendation (Table 13). In children and adolescents with moderate-to-severe atopic dermatitis, referral to a pediatric allergy specialist is recommended, as specialist assessment facilitates identification of allergic comorbidities, optimization of individualized treatment strategies, access to advanced therapies when indicated, and overall improvement in quality of life.

##### PICO 2

For children and adolescents with suspected AD, are standardized clinical diagnostic criteria (such as Hanifin and Rajka) better than diagnosis based only on medical history and clinical observation in diagnose the disease and reduce misdiagnoses?

Narrative report. In children and adolescents with suspected AD, the use of standardized and validated diagnostic criteria is essential to improve diagnostic accuracy, ensure consistent disease severity assessment, and reduce the risk of misclassification. Several studies have demonstrated that structured diagnostic approaches outperform unstructured clinical evaluation, particularly in patients with mild or atypical disease.

Minasyan et al. [89] validated the Comprehensive Early Childhood Allergy Questionnaire (CECAQ), demonstrating that standardized assessment tools achieve high diagnostic accuracy, with a sensitivity of 0.88 and a specificity of 0.83 for AD diagnosis. Similarly, Cheng et al. [90] developed and validated diagnostic criteria for Chinese children that achieved significantly higher sensitivity than the classic Hanifin and Rajka and UK Working Party criteria while maintaining comparable specificity, thereby improving the identification of mild and atypical presentations.

Reliable assessment of disease severity is equally important. A systematic review of randomized controlled trials highlighted considerable heterogeneity in the outcome measures used to evaluate AD severity and quality of life, emphasizing the need for standardized instruments such as SCORAD and EASI to improve consistency, comparability, and reproducibility across clinical settings [91]. In agreement with these findings, Barbarot et al. [92] demonstrated that the patient-oriented SCORAD (PO-SCORAD) strongly correlates with physician-assessed disease severity and provides a more sensitive measure of disease exacerbations than the conventional SCORAD, supporting its value for longitudinal monitoring, particularly in younger children.

Patient-reported outcome measures (PROMs) also contribute substantially to the comprehensive assessment of AD. Gabes et al. [93] emphasized the importance of validated PROMs for measuring health-related quality of life, recommending instruments specifically developed for pediatric eczema, such as the CADIS-SF for infants, while highlighting the limitations of less extensively validated questionnaires such as the DLQI. Likewise, the Pediatric Allergy Disease Quality of Life Questionnaire (PADQLQ), although useful for assessing multisystem allergic disease, is less sensitive than eczema-specific instruments such as POEM and CDLQI for evaluating AD severity and treatment response [94].

The clinical relevance of standardized assessment has also been demonstrated in longitudinal studies. Gazibara et al. [76] reported that improvements in disease severity, measured by SCORAD, were closely associated with improvements in quality of life, indicating that validated diagnostic and monitoring instruments facilitate a more accurate evaluation of disease burden and treatment response over time.

Current international consensus documents consistently recommend that both the diagnosis and severity classification of AD should be based on standardized and validated criteria [68,69,70,71]. Collectively, the available evidence indicates that structured diagnostic and monitoring tools improve diagnostic reliability, facilitate recognition of mild and atypical disease, reduce diagnostic variability, and support more appropriate therapeutic decision-making and long-term follow-up.

Recommendation (Table 14). In children and adolescents with suspected atopic dermatitis, the use of standardized and validated diagnostic criteria is conditionally recommended over unstructured clinical assessment. Standardized approaches improve diagnostic accuracy, facilitate consistent severity assessment, reduce diagnostic variability, and support appropriate management and longitudinal follow-up.

##### PICO 3

In children and adolescents with AD already on local therapy (steroid cream, emollients, specific cleansing products), can allergy tests (prick tests and specific IgE) identify possible food and environmental triggers and, compared to management based only on clinical evaluation, affect disease control and frequency of exacerbations?

Narrative report. No randomized controlled trials, systematic reviews, or meta-analyses have directly evaluated whether routine allergy testing (SPT or serum-specific IgE) improves disease control or reduces exacerbations in children and adolescents with AD. Consequently, current recommendations are primarily based on international guidelines and expert consensus rather than high-certainty evidence.

Overall, available guidelines consistently discourage the routine use of allergy tests in patients with AD. Instead, allergological investigations should be reserved for children with a clinical history suggestive of IgE-mediated reactions or reproducible worsening of eczema following exposure to specific foods or environmental allergens [68,70,73,95]. In this context, test results should always be interpreted together with the clinical history, as sensitization alone does not establish causality and should not lead to dietary or environmental restrictions in the absence of compatible symptoms.

The 2012 Joint Task Force (JTF) and the 2014 American Academy of Dermatology (AAD) guidelines both recommend a selective diagnostic approach [68]. The AAD explicitly advises against relying solely on allergy test results and discourages unnecessary elimination diets, whereas the JTF recommends targeted measurement of serum-specific IgE only in selected situations, such as immediate reactions following food ingestion or reproducible eczema exacerbations associated with specific exposures. Similarly, the international consensus by Lansang et al. [70] recommends avoiding indiscriminate food allergy testing and emphasizes that elimination diets should be guided by clinical symptoms rather than sensitization alone. The consensus document by Ortíz de Frutos et al. [73] also supports structured screening for relevant comorbidities but does not provide evidence that allergy testing improves AD control. Likewise, the most recent EAACI recommendations reserve measurement of total and specific IgE for patients with clinical features suggestive of IgE-mediated disease, including immediate reactions to foods or aeroallergens and selected phenotypes such as Malassezia-associated dermatitis [95].

The clinical relevance of allergic triggers varies according to age. Food allergens, including cow’s milk, egg, wheat, and peanut, are more frequently implicated during early childhood, whereas aeroallergens—particularly house dust mites—appear to play a greater role in older children and adolescents. Nevertheless, allergy testing should remain driven by a clear and reproducible clinical history rather than performed routinely.

Recent evidence further supports this selective strategy. A systematic review and meta-analysis commissioned by the AAAAI/ACAAI evaluated 23 randomized controlled trials investigating allergen immunotherapy in patients with AD predominantly sensitized to house dust mites [96]. Both subcutaneous (SCIT) and sublingual (SLIT) immunotherapy significantly improved disease severity and quality of life with moderate-certainty evidence. Importantly, these benefits were limited to patients with clinically relevant sensitization, reinforcing the need for careful patient selection through targeted allergy testing within an appropriate diagnostic pathway.

Conversely, evidence regarding food allergy is considerably less supportive of routine testing. Randomized trials and systematic reviews have shown that empirical or test-guided elimination diets provide only modest improvements in disease severity, with limited effects on pruritus and sleep quality [97]. Likewise, nutritional interventions—including probiotics, vitamins, and hydrolyzed formulas—have demonstrated only small and indirect benefits [98]. These findings further support avoiding unnecessary food allergy investigations and restrictive diets in children without convincing clinical evidence of immediate food allergy.

Collectively, the available evidence indicates that routine allergy testing does not improve disease control in pediatric AD. Rather, SPT and serum-specific IgE should be considered components of a targeted diagnostic strategy reserved for patients with a compatible clinical history. Appropriate interpretation of positive results is essential to avoid overdiagnosis, unnecessary elimination diets, nutritional deficiencies, and increased caregiver anxiety.

Recommendation (Table 15). A conditional recommendation is made against routine allergy testing (SPT and serum-specific IgE) in children and adolescents with atopic dermatitis. Targeted allergy investigations should instead be considered in patients with a clinical history suggestive of clinically relevant IgE-mediated sensitization or reproducible exacerbations following exposure to specific foods or aeroallergens.

##### PICO 4

In children and adolescents with moderate to severe atopic dermatitis, psychological support integrated into disease management is more effective than medical management alone in improving quality of life and reducing anxiety and depression?

Narrative report. Quality of life (QoL) and mental health can be compromised in cases of moderate to severe AD. Structured health education interventions can improve QoL and contribute to psychological well-being. However, there are no available RCTs or systematic reviews that directly compare integrated psychological support with medical management alone. Current evidence mainly derives from RCTs on educational interventions, systematic reviews on PROMs, and consensus documents on the management of AD comorbidities. In a systematic review on AD, only one-third of the 135 RCTs analyzed included a QoL assessment, suggesting an underrepresentation of psychosocial outcomes in clinical research on this condition [91]. The most commonly used QoL scales (e.g., IDQoL, CDLQI, PADQLQ) are not validated to assess the psychological impact in terms of anxiety or depression in children with AD [93,94].

In two meta-analyses on pediatric AD, health education interventions—both in-person and virtual—such as structured training for parents and caregivers, written eczema management plans, group sessions conducted by dermatology nurses, and digital or telemedicine counselling, have led to significant improvements in disease severity and QoL. However, no effect was observed on family QoL (e.g., CDLQI, DFI), and anxiety and depression were not systematically evaluated [93,94].

In an RCT conducted by Pustišek et al. [74], a structured educational intervention aimed at parents not only improved the severity of eczema in children but also significantly reduced parental stress and anxiety and improved family QoL (measured by FDLQI). Although the treatment of AD is strongly correlated with the improvement of the patient’s QoL, the psychological burden persists over time even when skin symptoms improve, suggesting the need for adequate mental health screening and psychological support.

Other studies show that structured educational interventions, although not formally classified as psychological therapies, have significant benefits on QoL and emotional well-being in children with AD and their families. Some authors [81] found that a multidisciplinary educational program for caregivers, conducted in a hospital setting, significantly improved QoL scores (measured by IDQoL and DFI) and reduced parental stress, suggesting that educational support can positively influence psychological outcomes. Similarly, another group (16), reported that personalized education conducted by nurses improved disease control and family functioning, highlighting the importance of accessible and tailored support. Finally, more recent evidence from Salava et al. [75] and Gazibara et al. [76] highlights that early and active involvement through education, combined with effective topical therapy, can reduce the emotional burden and enhance the management capacity of both patients and caregivers.

Various consensus documents [68,69,70,71,73,99] strongly recommend systematic screening for psychiatric comorbidities (depression, anxiety, ADHD) in children and adolescents with moderate to severe AD, and promote a multidisciplinary approach that includes psychological support when necessary. However, further studies are needed to evaluate psychological interventions in children with AD.

Recommendation (Table 16). A conditional recommendation was formulated in favour of structured educational interventions as part of routine management in children and adolescents with moderate-to-severe atopic dermatitis. Psychological assessment and referral for counselling or psychological support should be considered in patients and families experiencing significant emotional or psychosocial burden.

## 4. Discussion

This work provides evidence-based recommendations for the appropriateness of diagnostic prescriptions in pediatric allergology, focusing on food allergy, eosinophilic gastrointestinal disorders, chronic urticaria, and angioedema. The primary objective was to support evidence-informed clinical decision-making while promoting more rational use of healthcare resources, reducing unnecessary investigations, and improving equity of access to specialist care.

Overall, the available evidence supports a selective and structured diagnostic approach across the clinical conditions considered. Rather than promoting extensive diagnostic testing, the present recommendations emphasize the central role of careful history taking and physical examination, followed by investigations selected according to the clinical presentation and their expected contribution to diagnosis or management [29,90,100,101]. At the same time, the findings identify specific clinical settings in which specialist assessment and second-level investigations remain essential and should not be delayed. Therefore, diagnostic appropriateness should not be interpreted simply as a reduction in testing, but rather as the appropriate selection and timing of investigations according to the suspected condition and individual clinical context [33,90,102].

For food allergy, the evidence confirms the pivotal role of specialist multidisciplinary assessment and indicates that sensitization tests alone are insufficient to establish a diagnosis. Skin prick tests and serum-specific IgE should be interpreted within the clinical context, while oral food challenge remains central to diagnostic confirmation when indicated [25,31,34,103]. This approach is particularly relevant because inappropriate interpretation of sensitization may result in overdiagnosis and unnecessary elimination diets, with potential nutritional and psychosocial consequences [104,105]. Specialist assessment may therefore improve diagnostic precision and support individualized management while limiting unnecessary dietary restrictions.

A similar principle applies to eosinophilic gastrointestinal disorders, although the diagnostic pathway differs substantially. In this setting, endoscopic assessment with histological evaluation has a central role in confirming the diagnosis, whereas allergological investigations should be regarded as complementary rather than diagnostic in themselves [35,36,40,44,106,107]. The recommendations therefore emphasize the need to distinguish investigations required for diagnostic confirmation from those that may subsequently contribute to the characterization and management of the individual patient [44,106].

In CSU, the available evidence supports a conservative diagnostic strategy based primarily on clinical evaluation. Routine allergological investigations provide limited diagnostic benefit in the absence of a suggestive clinical history, as allergen sensitization does not necessarily identify the underlying cause of CSU [47,54,55,56,57,59]. Accordingly, skin prick tests and serum-specific IgE should be reserved for selected patients in whom an allergic trigger is clinically suspected. Likewise, investigation of autoimmune comorbidities should follow a targeted approach, with particular consideration given to thyroid dysfunction and coeliac disease and further investigations guided by symptoms, personal or family history of autoimmunity, or refractory disease [25,103,108]. This strategy may improve diagnostic appropriateness while limiting false-positive findings, unnecessary investigations, and inappropriate management. Conversely, specialist referral assumes greater importance in patients with persistent or treatment-resistant urticaria, facilitating appropriate reassessment and access to advanced therapeutic options when indicated. Recurrent angioedema represents another condition in which a structured diagnostic pathway is particularly important. The distinction between histamine-mediated and bradykinin-mediated forms should be based on clinical characteristics and targeted investigations, particularly when hereditary angioedema is suspected. Early recognition of hereditary forms through appropriate laboratory assessment is essential for establishing disease-specific management and preventing potentially severe complications.

In the context of atopic dermatitis, the formulated recommendations similarly emphasize an integrated and individualized approach, particularly in children with moderate-to-severe disease [69,70,73,96,99,109,110,111]. Standardized assessment of disease severity is important for defining subsequent management and identifying patients who may require advanced treatment, including biological therapies. Specialist allergological assessment and targeted testing should be considered according to the individual clinical presentation rather than routinely applied to all patients. Importantly, management extends beyond diagnostic and pharmacological interventions: structured educational programmes represent an important component of care, while psychological assessment and support should be considered according to the emotional and psychosocial burden experienced by individual patients and their families.

Taken together, the findings identify a common principle across the different areas of pediatric allergology addressed by this work: diagnostic appropriateness requires a balance between avoiding indiscriminate testing and ensuring timely access to investigations that can meaningfully confirm a diagnosis, identify clinically relevant comorbidities, or modify patient management. Their integration into Diagnostic and Therapeutic Care Pathways (PDTAs) may contribute to harmonizing clinical practice across different healthcare settings and improving collaboration between primary care and specialist services. Continuous education of healthcare professionals will be essential to facilitate implementation of these recommendations in routine clinical practice.

Limitations. Several methodological limitations should be acknowledged. First, the overall certainty of evidence varied across the different PICO questions and was frequently limited by the predominance of observational studies, with relatively few randomized controlled trials directly addressing diagnostic strategies. Second, many recommendations were necessarily supported by indirect evidence derived from diagnostic accuracy studies, systematic reviews, and international clinical guidelines rather than direct comparative studies evaluating alternative diagnostic pathways. A further limitation is that the study protocol was not prospectively registered in a publicly accessible systematic review registry. Nevertheless, the methodological approach, eligibility criteria, PICO questions, and procedures for evidence appraisal were defined a priori by the multidisciplinary working group within the framework provided by the Italian National Health Institute [6,7]. The lack of prospective registration therefore does not imply that the review was conducted without a predefined methodology, but it limits the possibility for external readers to independently verify protocol adherence and to assess whether any methodological decisions were modified during the review process. Finally, the rapid evolution of pediatric allergology, particularly regarding biologic therapies and molecular diagnostics, will require periodic updating of these recommendations as new evidence becomes available.

## 5. Conclusions

The systematic application of the GCPR can improve the quality of care in pediatric allergology, optimize the use of healthcare resources, ensure equitable access to services, reduce waiting lists, and mitigate territorial disparities. However, the active involvement of all stakeholders is necessary to transform these recommendations into daily practice.

## Figures and Tables

**Table 1:** Summary of inquired PICOs on IgE-mediated and non-IgE mediated food allergy and eosinophilic gastrointestinal disease.

	PICO 1	PICO 2	PICO 3	PICO 4
Population	children and adolescents with suspected food allergy and atopic dermatitis	children and adolescents with suspected food allergy	children and adolescents with suspected eosinophilic gastrointestinal disease or non-IgE-mediated food allergy	in children and adolescents with suspected eosinophilic gastrointestinal disease
(age)	<18 years	<18 years	<18 years	<18 years
Intervention	allergy consultation	use of the oral food challenge	specialist gastroenterology/allergy consultation	endoscopy with biopsy
Comparator	the regular follow-up with a primary care pediatrician	diagnosis based solely on skin prick tests and specific IgE measurements	independent management by the primary care paediatrician	serological and skin tests
Outcome	initiate specific therapy and improve quality of life	reducing overdiagnosis and ensuring an adequate diagnosis of food allergy	achieving an accurate diagnosis and initiating specific treatment	confirming the diagnosis and guiding therapy

**Table 2:** Summary of quality of evidence on the PICO 1 (i.e., “In children and adolescents with suspected food allergy and atopic dermatitis, is an allergy consultation recommended over the regular follow-up with a primary care pediatrician to initiate specific therapy and improve quality of life?”). Note: GRADE = Grading of Recommendations Assessment, Development and Evaluation.

Quality of Evidence (GRADE)	Strength	Balance of Benefits/Risks	Values and Preferences	Impact on Resources/ Implementation
⨁⨁◯◯	Conditional recommendation in favour of specialist referral	Improved diagnostic accuracy, appropriate use of elimination diets and OFC, earlier targeted therapy, and better quality of life outweigh the minimal risks associated with specialist referral.	Families generally value specialist assessment and accurate diagnosis, although preferences may vary according to accessibility and waiting times.	Implementation requires adequate pediatric allergy services and may increase short-term resource use; however, improved diagnostic appropriateness may reduce unnecessary investigations, inappropriate dietary restrictions, and repeated healthcare utilization, potentially leading to long-term savings.

Note: ⨁⨁⨁⨁ = High certainty of evidence; ⨁⨁⨁◯ = Moderate certainty of evidence; ⨁⨁◯◯ = Low certainty of evidence; ⨁◯◯◯ = Very Low certainty of evidence; ◯◯◯◯ = absence of evidence answering the PICO.

**Table 3:** Summary of quality of evidence on the PICO 2 (i.e., “In children with suspected food allergy, is the use of the OFC more effective than diagnosis based solely on skin prick tests and specific IgE measurements in reducing overdiagnosis and ensuring an adequate diagnosis of food allergy?”). Note: GRADE = Grading of Recommendations Assessment, Development and Evaluation.

Quality of Evidence (GRADE)	Strength	Balance of Benefits/Risks	Values and Preferences	Impact on Resources/ Implementation
⨁⨁⨁⨁	Strongly in favour of intervention	OFC provides the highest diagnostic accuracy, reduces overdiagnosis and unnecessary elimination diets, and supports appropriate long-term management. When performed in experienced centers, its benefits clearly outweigh the limited procedural risks.	Families and clinicians highly value a definitive diagnosis, avoidance of unnecessary dietary restrictions, and safe dietary reintroduction whenever appropriate.	OFC requires trained personnel, appropriate facilities, and emergency support, which may limit its availability in peripheral centers. However, improved diagnostic accuracy may reduce unnecessary investigations, follow-up, and long-term dietary management costs.

Note: ⨁⨁⨁⨁ = High certainty of evidence; ⨁⨁⨁◯ = Moderate certainty of evidence; ⨁⨁◯◯ = Low certainty of evidence; ⨁◯◯◯ = Very Low certainty of evidence; ◯◯◯◯ = absence of evidence answering the PICO.

**Table 4:** Summary of quality of evidence on the PICO 3 (i.e., “In children and adolescents with suspected eosinophilic gastrointestinal disease or non-IgE-mediated food allergy, is a specialist gastroenterology/allergy consultation recommended compared to independent management by the primary care paediatrician for achieving an accurate diagnosis and initiating specific treatment?”). Note: GRADE = Grading of Recommendations Assessment, Development and Evaluation.

Quality of Evidence (GRADE)	Strength	Balance of Benefits/Risks	Values and Preferences	Impact on Resources/ Implementation
⨁⨁◯◯	Conditional recommendation in favour of specialist referral	Specialist multidisciplinary assessment improves diagnostic accuracy, facilitates appropriate endoscopic and histological evaluation, supports individualized management, and may reduce unnecessary elimination diets and delayed diagnosis. Benefits are likely to outweigh the limited disadvantages associated with specialist referral.	Families generally value specialist assessment because of its potential to provide diagnostic certainty and individualized management, although waiting times and geographical accessibility may influence preferences.	Implementation requires multidisciplinary pediatric allergy and gastroenterology services, endoscopy facilities, and pathology expertise. Although this may increase short-term resource utilization, earlier diagnosis may reduce repeated investigations, prolonged elimination diets, and delayed treatment.

Note: ⨁⨁⨁⨁ = High certainty of evidence; ⨁⨁⨁◯ = Moderate certainty of evidence; ⨁⨁◯◯ = Low certainty of evidence; ⨁◯◯◯ = Very Low certainty of evidence; ◯◯◯◯ = absence of evidence answering the PICO.

**Table 5:** Summary of quality of evidence on the PICO 4 (i.e., “In children and adolescents with suspected eosinophilic gastrointestinal disease, is endoscopy with biopsy more strongly recommended than serological and skin tests alone for confirming the diagnosis and guiding therapy?”). Note: GRADE = Grading of Recommendations Assessment, Development and Evaluation.

Quality of Evidence (GRADE)	Strength	Balance of Benefits/Risks	Values and Preferences	Impact on Resources/ Implementation
⨁⨁⨁◯	Strong recommendation in favour of endoscopic evaluation with biopsy	Histological confirmation is essential for establishing the diagnosis, excluding alternative causes of gastrointestinal eosinophilia, and guiding individualized treatment. The diagnostic benefits clearly outweigh the procedural risks when endoscopy is performed in experienced pediatric centers.	Families generally value a definitive diagnosis and appropriate treatment. Although the invasive nature of endoscopy may raise concerns, acceptance is usually high when its diagnostic role is adequately explained.	Implementation requires access to pediatric endoscopy services, anesthesia support, and experienced pathologists. Although resource-intensive, these requirements are justified by the substantial improvement in diagnostic accuracy and subsequent patient management.

Note: ⨁⨁⨁⨁ = High certainty of evidence; ⨁⨁⨁◯ = Moderate certainty of evidence; ⨁⨁◯◯ = Low certainty of evidence; ⨁◯◯◯ = Very Low certainty of evidence; ◯◯◯◯ = absence of evidence answering the PICO.

**Table 6:** Summary of inquired PICOs on urticaria and angio-edema.

	PICO 1	PICO 2	PICO 3	PICO 4	PICO 5
Population	children and adolescents with chronic urticaria	children and adolescents with chronic urticaria	children and adolescents with chronic urticaria	children and adolescents with recurrent episodes of angioedema without urticaria	children and adolescents with isolated recurrent angioedema without urticaria and without obvious
(age)	<18 years	<18 years	<18 years	<18 years	<18 years
Intervention	allergy consultation	routine performance of allergological tests (skin prick test, specific IgE)	targeted diagnostic approach based on clinical history	specialist allergological and/or immunological consultation	performance of specific laboratory tests (C1 inhibitor, C4, and C1q measurement)
Comparator	standard follow-up by the general paediatrician	management based solely on clinical history	extensive diagnostic work-up with non-targeted allergological and autoimmune tests	management by the primary care paediatrician alone	clinical observation alone
Outcome	initiate specific therapy and improve quality of life	improve the identification of triggering causes	improving diagnostic appropriateness, including reducing costs, increasing correct diagnoses, and reducing unnecessary tests	identify possible hereditary or acquired forms and improve disease management	differentiating allergic forms from hereditary or acquired C1 inhibitor deficiency

**Table 7:** Summary of quality of evidence on the PICO 1 (i.e., “In children and adolescents with chronic urticaria, is an allergological consultation recommended over standard follow-up by the general paediatrician in order to initiate specific therapy and improve quality of life?”). Note: GRADE = Grading of Recommendations Assessment, Development and Evaluation.

Quality of Evidence (GRADE)	Strength	Balance of Benefits/Risks	Values and Preferences	Impact on Resources/ Implementation
⨁⨁◯◯	Conditional recommendation in favour of specialist referral for selected patients	Specialist assessment facilitates diagnostic confirmation, optimization of therapy, and access to advanced treatments (e.g., biologics) in refractory CSU. These benefits are likely to outweigh the disadvantages associated with referral, particularly in persistent or treatment-resistant disease.	Families generally value symptom control, reassurance, and access to effective therapies. Acceptance of specialist referral is high when symptoms persist, first-line therapy fails, or quality of life is significantly impaired.	Implementation requires access to pediatric allergy services and clear referral pathways. Resource utilization is moderate and can be optimized by referring children with persistent, recurrent, or antihistamine-refractory CSU.

Note: ⨁⨁⨁⨁ = High certainty of evidence; ⨁⨁⨁◯ = Moderate certainty of evidence; ⨁⨁◯◯ = Low certainty of evidence; ⨁◯◯◯ = Very Low certainty of evidence; ◯◯◯◯ = absence of evidence answering the PICO.

**Table 8:** Summary of quality of evidence on the PICO 2 (i.e., “In children and adolescents with chronic urticaria, does the routine performance of allergological tests (skin prick test, specific IgE) compared to management based solely on clinical history improve the identification of triggering causes?”). Note: GRADE = Grading of Recommendations Assessment, Development and Evaluation.

Quality of Evidence (GRADE)	Strength	Balance of Benefits/Risks	Values and Preferences	Impact on Resources/ Implementation
⨁⨁⨁◯	Strong recommendation against routine allergological investigations. Targeted testing is recommended only when supported by the clinical history.	Routine allergy testing provides little diagnostic benefit in the absence of clinical suspicion. Avoiding unnecessary investigations reduces false-positive results, inappropriate management, healthcare costs, and parental anxiety without compromising diagnostic accuracy.	Families often expect comprehensive allergy testing. Appropriate counselling is therefore essential to explain the limited diagnostic value of routine investigations and to align expectations with current evidence.	Restricting allergy testing to patients with a suggestive clinical history reduces unnecessary investigations, improves resource utilization, and can be readily implemented through standardized history-based diagnostic pathways.

Note: ⨁⨁⨁⨁ = High certainty of evidence; ⨁⨁⨁◯ = Moderate certainty of evidence; ⨁⨁◯◯ = Low certainty of evidence; ⨁◯◯◯ = Very Low certainty of evidence; ◯◯◯◯ = absence of evidence answering the PICO.

**Table 9:** Summary of quality of evidence on the PICO 3 (i.e., “In children and adolescents with chronic urticaria, is a targeted diagnostic approach based on clinical history, compared to an extensive diagnostic work-up with non-targeted allergological and autoimmune tests, more effective in improving diagnostic appropriateness, including reducing costs, increasing correct diagnoses, and reducing unnecessary tests?”). Note: GRADE = Grading of Recommendations Assessment, Development and Evaluation.

Quality of Evidence (GRADE)	Strength	Balance of Benefits/Risks	Values and Preferences	Impact on Resources/ Implementation
⨁⨁⨁◯	Conditional recommendation in favour of a targeted diagnostic approach	A targeted diagnostic strategy is expected to improve diagnostic appropriateness, facilitate the identification of clinically relevant autoimmune comorbidities, and reduce unnecessary investigations, healthcare costs, and patient burden. These expected benefits are considered to outweigh the small risk of missing uncommon conditions, provided that careful clinical assessment and appropriate follow-up are ensured.	Families generally value diagnostic pathways that avoid unnecessary testing while identifying clinically relevant conditions. Appropriate counselling improves acceptance of a selective diagnostic strategy.	Implementation is straightforward through standardized history-taking, focused first-line investigations, and selective second-level testing. This approach may reduce unnecessary laboratory investigations and promote more appropriate use of healthcare resources.

Note: ⨁⨁⨁⨁ = High certainty of evidence; ⨁⨁⨁◯ = Moderate certainty of evidence; ⨁⨁◯◯ = Low certainty of evidence; ⨁◯◯◯ = Very Low certainty of evidence; ◯◯◯◯ = absence of evidence answering the PICO.

**Table 10:** Summary of quality of evidence on the PICO 4 (i.e., “In children and adolescents with recurrent episodes of angioedema without urticaria, is a specialist allergological and/or immunological consultation recommended compared to management by the primary care paediatrician alone, in order to identify possible hereditary or acquired forms and improve disease management?”). Note: GRADE = Grading of Recommendations Assessment, Development and Evaluation.

Quality of Evidence (GRADE)	Strength	Balance of Benefits/Risks	Values and Preferences	Impact on Resources/ Implementation
⨁⨁⨁◯	Strong recommendation in favour of specialist referral	Specialist referral facilitates accurate differentiation between hereditary and acquired forms of angioedema, enabling appropriate disease-specific treatment, long-term follow-up, and prevention of potentially life-threatening complications. The benefits clearly outweigh the limited disadvantages associated with referral.	Patients and families place high value on obtaining a definitive diagnosis, receiving appropriate counselling, and having an individualized emergency and long-term management plan. Acceptance of specialist referral is expected to be high.	Implementation requires access to specialist referral centres and well-defined referral pathways. Given the rarity of hereditary angioedema, the overall impact on healthcare resources is expected to be limited, while the potential clinical benefit is substantial.

Note: ⨁⨁⨁⨁ = High certainty of evidence; ⨁⨁⨁◯ = Moderate certainty of evidence; ⨁⨁◯◯ = Low certainty of evidence; ⨁◯◯◯ = Very Low certainty of evidence; ◯◯◯◯ = absence of evidence answering the PICO.

**Table 11:** Summary of quality of evidence on the PICO 5 (i.e., “In children and adolescents with isolated recurrent angioedema without urticaria and without obvious causes, is the performance of specific laboratory tests (C1 inhibitor, C4, and C1q measurement) more appropriate than clinical observation alone for differentiating allergic forms from hereditary or acquired C1 inhibitor deficiency?”). Note: GRADE = Grading of Recommendations Assessment, Development and Evaluation.

Quality of Evidence (GRADE)	Strength	Balance of Benefits/Risks	Values and Preferences	Impact on Resources/ Implementation
⨁⨁⨁◯	Strong recommendation in favour of targeted laboratory investigation	Targeted laboratory assessment facilitates differentiation between hereditary, acquired, and histamine-mediated angioedema, enabling timely diagnosis, appropriate disease-specific management, and prevention of potentially life-threatening complications. The benefits clearly outweigh the minimal risks associated with blood sampling.	Patients and families place high value on establishing a definitive diagnosis, preventing recurrent attacks, and receiving an individualized management plan. Acceptance of laboratory investigations is generally high.	Measurement of C4 and quantitative and functional C1-INH is widely available in most referral laboratories. Additional investigations (e.g., C1q measurement or genetic testing) should be reserved for selected patients according to clinical suspicion, making implementation feasible and economically sustainable.

Note: ⨁⨁⨁⨁ = High certainty of evidence; ⨁⨁⨁◯ = Moderate certainty of evidence; ⨁⨁◯◯ = Low certainty of evidence; ⨁◯◯◯ = Very Low certainty of evidence; ◯◯◯◯ = absence of evidence answering the PICO.

**Table 12:** Summary of inquired PICOs on atopic dermatitis.

	PICO 1	PICO 2	PICO 3	PICO 4
Population	children and adolescents with severe DA	children and adolescents with suspected AD	AD already on local therapy (steroid cream, emollients, specific cleansing products)	in children and adolescents with moderate to severe atopic dermatitis
(age)	<18 years	<18 years	<18 years	<18 years
Intervention	allergy consultation	standardized clinical diagnostic criteria (such as Hanifin and Rajka)	allergy tests (prick tests and specific IgE)	psychological support integrated into disease management
Comparator	routine follow-up with a general pediatrician	diagnosis based only on medical history and clinical observation	management based only on clinical evaluation	medical management alone
Outcome	initiate specific therapy and improve quality of life	diagnose the disease and reduce misdiagnoses	identify possible food and environmental triggers, affect disease control and frequency of exacerbations	improving quality of life and reducing anxiety and depression

**Table 13:** Summary of quality of evidence on the PICO 1 (i.e., “In children and adolescents with severe atopic dermatitis, is allergy consultation recommended over routine follow-up with a general pediatrician to initiate specific therapy and improve quality of life?”). Note: GRADE = Grading of Recommendations Assessment, Development and Evaluation.

Quality of Evidence (GRADE)	Strength	Balance of Benefits/Risks	Values and Preferences	Impact on Resources/ Implementation
⨁⨁⨁◯	Strong recommendation in favour of specialist referral	Early specialist assessment improves diagnostic accuracy, facilitates identification of allergic comorbidities, enables timely access to advanced therapies and structured educational programmes, and improves quality of life. These benefits outweigh the additional costs associated with specialist care.	Families place a high value on specialist evaluation, personalized management, and improved disease control, resulting in high acceptability.	Implementation requires access to pediatric allergy specialists and dedicated outpatient services. Although availability may vary across geographical areas, long-term resource utilization is expected to be sustainable through improved diagnostic appropriateness and reduced disease burden.

Note: ⨁⨁⨁⨁ = High certainty of evidence; ⨁⨁⨁◯ = Moderate certainty of evidence; ⨁⨁◯◯ = Low certainty of evidence; ⨁◯◯◯ = Very Low certainty of evidence; ◯◯◯◯ = absence of evidence answering the PICO.

**Table 14:** Summary of quality of evidence on the PICO 2 (i.e., “For children and adolescents with suspected atopic dermatitis, are standardized clinical diagnostic criteria (such as Hanifin and Rajka) better than diagnosis based only on medical history and clinical observation in diagnose the disease and reduce misdiagnoses?”). Note: GRADE = Grading of Recommendations Assessment, Development and Evaluation.

Quality of Evidence (GRADE)	Strength	Balance of Benefits/Risks	Values and Preferences	Impact on Resources/ Implementation
⨁⨁⨁◯	Conditional recommendation in favour of standardized diagnostic criteria	Use of validated diagnostic criteria improves diagnostic consistency, reduces misclassification, facilitates disease severity assessment, and supports more appropriate long-term management. Potential disadvantages are minimal and primarily related to implementation.	Specialists generally value standardized diagnostic tools. Broader implementation in primary care may require additional training and familiarity with validated assessment instruments.	Implementation costs are low and mainly related to dissemination of guidelines and professional training. Adoption of standardized diagnostic criteria is feasible across different healthcare settings.

Note: ⨁⨁⨁⨁ = High certainty of evidence; ⨁⨁⨁◯ = Moderate certainty of evidence; ⨁⨁◯◯ = Low certainty of evidence; ⨁◯◯◯ = Very Low certainty of evidence; ◯◯◯◯ = absence of evidence answering the PICO.

**Table 15:** Summary of quality of evidence on the PICO 3 (i.e., “In children and adolescents with atopic dermatitis already on local therapy (steroid cream, emollients, specific cleansing products), can allergy tests (prick tests and specific IgE) identify possible food and environmental triggers and, compared to management based only on clinical evaluation, affect disease control and frequency of exacerbations?”). Note: GRADE = Grading of Recommendations Assessment, Development and Evaluation.

Quality of Evidence (GRADE)	Strength	Balance of Benefits/Risks	Values and Preferences	Impact on Resources/ Implementation
⨁⨁◯◯	Conditional recommendation in favour of targeted allergy testing (against routine testing)	Routine allergy testing provides little clinical benefit and may lead to overdiagnosis, unnecessary elimination diets, nutritional risks, and caregiver anxiety. Targeted testing based on a compatible clinical history improves diagnostic appropriateness and patient selection.	Families often expect allergy testing; therefore, appropriate counselling is essential to explain the limited value of routine investigations and the rationale for a selective diagnostic approach.	Restricting allergy testing to selected patients reduces unnecessary investigations and healthcare costs. Implementation requires dissemination of guideline-based diagnostic algorithms and appropriate clinician training.

Note: ⨁⨁⨁⨁ = High certainty of evidence; ⨁⨁⨁◯ = Moderate certainty of evidence; ⨁⨁◯◯ = Low certainty of evidence; ⨁◯◯◯ = Very Low certainty of evidence; ◯◯◯◯ = absence of evidence answering the PICO.

**Table 16:** Summary of quality of evidence on the PICO 4 (i.e., “In children and adolescents with moderate to severe atopic dermatitis, psychological support integrated into disease management is more effective than medical management alone in improving quality of life and reducing anxiety and depression?”). Note: GRADE = Grading of Recommendations Assessment, Development and Evaluation.

Quality of Evidence (GRADE)	Strength	Balance of Benefits/Risks	Values and Preferences	Impact on Resources/ Implementation
⨁⨁⨁◯	Conditional recommendation; structured educational interventions are supported by the available evidence, while psychological support should be considered according to individual clinical needs.	Structured educational programmes improve disease severity, treatment adherence, and quality of life. Selected patients may derive additional benefit from psychological assessment and support. Overall, the expected benefits outweigh the additional resource requirements.	Patients, families, and clinicians place high value on interventions that improve disease control, quality of life, and psychosocial well-being.	Implementation requires multidisciplinary teams and access to educational and psychological services. Availability of pediatric psychologists may limit implementation in some healthcare settings, and resource requirements should therefore be considered according to local availability and individual clinical needs.

Note: ⨁⨁⨁⨁ = High certainty of evidence; ⨁⨁⨁◯ = Moderate certainty of evidence; ⨁⨁◯◯ = Low certainty of evidence; ⨁◯◯◯ = Very Low certainty of evidence; ◯◯◯◯ = absence of evidence answering the PICO.

## Data Availability

The original contributions presented in this study are included in the article. Further inquiries can be directed to the corresponding author.

## Appendix A

**Table A1:** Summary of search strategies.

PUBMED	EMBASE	COCHRANE LIBRARY
**IgE-Mediated and Non-IgE Mediated Food Allergy and Eosinophilic Gastrointestinal Disease**		
Research Question (PICO) 1		
((“Food Hypersensitivity”[Mesh] OR “food allergy”[tiab]) AND (“Child”[Mesh] OR “Adolescent”[Mesh] OR child*[tiab] OR adolescen*[tiab] OR pediatric*[tiab]) AND (“Patient Care Planning”[Mesh] OR “written action plan”[tiab] OR “emergency plan”[tiab] OR “written plan”[tiab]) AND (“Treatment Outcome”[Mesh] OR “symptom improvement”[tiab] OR “clinical improvement”[tiab] OR “quality of life”[tiab] OR “QoL”[tiab]))	(‘food hypersensitivity’/exp OR ‘food allergy’:ti,ab) AND (‘child’/exp OR ‘adolescent’/exp OR child*:ti,ab OR adolescen*:ti,ab OR pediatric*:ti,ab) AND (‘care plan’ OR ‘written action plan’:ti,ab OR ‘emergency plan’:ti,ab OR ‘written plan’:ti,ab) AND (‘treatment outcome’/exp OR ‘symptom improvement’:ti,ab OR ‘clinical improvement’:ti,ab OR ‘quality of life’/exp OR ‘quality of life’:ti,ab OR qol:ti,ab)	#1 MeSH descriptor: [Food Hypersensitivity] explode all trees #2 “food allergy”:ti,ab,kw #3 #1 OR #2 #4 MeSH descriptor: [Child] explode all trees #5 MeSH descriptor: [Adolescent] explode all trees #6 (child* OR adolescen* OR pediatric*):ti,ab,kw #7 #4 OR #5 OR #6 #8 MeSH descriptor: [Patient Care Planning] explode all trees #9 (“written action plan” OR “emergency plan” OR “written plan”):ti,ab,kw #10 #8 OR #9 #11 MeSH descriptor: [Treatment Outcome] explode all trees #12 (“symptom improvement” OR “clinical improvement” OR “quality of life” OR QoL):ti,ab,kw #13 #11 OR #12 #14 #3 AND #7 AND #10 AND #13
Research Question (PICO) 2		
(“Food Hypersensitivity”[Mesh] OR “food allergy”[tiab]) AND (“Child”[Mesh] OR “Adolescent”[Mesh] OR child*[tiab] OR adolescen*[tiab] OR pediatric*[tiab]) AND (“Psychological Support”[tiab] OR “psychological intervention”[tiab] OR “mental health support”[tiab] OR counseling[tiab] OR “psychotherapy”[tiab] OR “psychological therapy”[tiab]) AND (“Quality of Life”[Mesh] OR “quality of life”[tiab] OR “QoL”[tiab] OR anxiety[tiab] OR “food allergy anxiety”[tiab] OR “anxiety scale”[tiab] OR “self-management”[tiab] OR autonomy[tiab] OR “epinephrine auto-injector”[tiab] OR “adrenaline auto-injector”[tiab] OR “social avoidance”[tiab] OR “family quality of life”[tiab] OR “caregiver burden”[tiab]))	(‘food hypersensitivity’/exp OR ‘food allergy’:ti,ab) AND (‘child’/exp OR ‘adolescent’/exp OR child*:ti,ab OR adolescen*:ti,ab OR pediatric*:ti,ab) AND (‘psychological care’/exp OR ‘psychological support’:ti,ab OR ‘psychological intervention’:ti,ab OR counseling:ti,ab OR ‘mental health’:ti,ab OR ‘psychotherapy’:ti,ab) AND (‘quality of life’/exp OR ‘quality of life’:ti,ab OR qol:ti,ab OR ‘anxiety disorder’:ti,ab OR ‘food allergy anxiety’:ti,ab OR ‘anxiety scale’:ti,ab OR ‘self-management’:ti,ab OR autonomy:ti,ab OR ‘epinephrine auto injector’:ti,ab OR ‘adrenaline auto injector’:ti,ab OR ‘social avoidance’:ti,ab OR ‘family quality of life’:ti,ab OR ‘caregiver burden’:ti,ab)	#1 MeSH descriptor: [Food Hypersensitivity] explode all trees #2 “food allergy”:ti,ab,kw #3 #1 OR #2 #4 MeSH descriptor: [Child] explode all trees #5 MeSH descriptor: [Adolescent] explode all trees #6 (child* OR adolescen* OR pediatric*):ti,ab,kw #7 #4 OR #5 OR #6 #8 (“Psychological Support” OR “psychological intervention” OR “mental health support” OR counseling OR psychotherapy OR “psychological therapy”):ti,ab,kw #9 MeSH descriptor: [Quality of Life] explode all trees #10 (“quality of life” OR QoL OR anxiety OR “food allergy anxiety” OR “anxiety scale” OR “self-management” OR autonomy OR “epinephrine auto-injector” OR “adrenaline auto-injector” OR “social avoidance” OR “family quality of life” OR “caregiver burden”):ti,ab,kw #11 #9 OR #10 #12 #3 AND #7 AND #8 AND #11
Research Question (PICO) 3		
(“Food Hypersensitivity”[MeSH] OR “Food Allergy”[tiab] OR “alimentary allergy”[tiab] OR “Atopic Dermatitis”[MeSH] OR “atopic dermatitis”[tiab]) AND (“Ambulatory Care”[MeSH] OR “Outpatients”[MeSH] OR “Home Care Services”[MeSH] OR “Day Care”[MeSH] OR “ambulatory care”[tiab] OR “outpatient care”[tiab] OR “home visit*”[tiab] OR “day care”[tiab]) AND (“Medication Adherence”[MeSH] OR “Patient Compliance”[MeSH] OR “Disease Management”[MeSH] OR “medication compliance”[tiab] OR “patient compliance”[tiab] OR “disease management”[tiab]) AND (“Child”[Mesh] OR “Adolescent”[Mesh] OR child*[tiab] OR adolescen*[tiab] OR pediatric*[tiab])	((‘alimentary allergy’ OR (alimentary AND (‘allergy’/exp OR allergy))) OR ‘atopic dermatitis’ OR ‘food allergy’) AND (‘ambulatory care’ OR ‘outpatient care’ OR ‘home visit’ OR ‘day care’) AND (‘medication compliance’ OR ‘disease management’ OR ‘patient compliance’) AND (‘child’/exp OR ‘adolescent’/exp OR child*:ti,ab OR adolescen*:ti,ab OR pediatric*:ti,ab)	#1 MeSH descriptor: [Food Hypersensitivity] explode all trees #2 MeSH descriptor: [Atopic Dermatitis] explode all trees #3 (“Food Allergy” OR “alimentary allergy” OR “atopic dermatitis”):ti,ab,kw #4 #1 OR #2 OR #3 #5 MeSH descriptor: [Ambulatory Care] explode all trees #6 MeSH descriptor: [Outpatients] explode all trees #7 MeSH descriptor: [Home Care Services] explode all trees #8 MeSH descriptor: [Day Care] explode all trees #9 (“ambulatory care” OR “outpatient care” OR “home visit*” OR “day care”):ti,ab,kw #10 #5 OR #6 OR #7 OR #8 OR #9 #11 MeSH descriptor: [Medication Adherence] explode all trees #12 MeSH descriptor: [Patient Compliance] explode all trees #13 MeSH descriptor: [Disease Management] explode all trees #14 (“medication compliance” OR “patient compliance” OR “disease management”):ti,ab,kw #15 #11 OR #12 OR #13 OR #14 #16 MeSH descriptor: [Child] explode all trees #17 MeSH descriptor: [Adolescent] explode all trees #18 (child* OR adolescen* OR pediatric*):ti,ab,kw #19 #16 OR #17 OR #18 #20 #4 AND #10 AND #15 AND #19
Research Question (PICO) 4		
(“Food Hypersensitivity”[MeSH] OR “Food Allergy”[tiab] OR “alimentary allergy”[tiab] OR “Atopic Dermatitis”[MeSH] OR “atopic dermatitis”[tiab]) AND (“Endoscopy, Gastrointestinal”[MeSH] OR “digestive tract endoscopy”[tiab] OR “Skin Tests”[MeSH] OR “prick test”[tiab] OR “Immunoglobulin E”[MeSH] OR “IgE”[tiab]) AND (“Sensitivity and Specificity”[MeSH] OR “diagnostic accuracy”[tiab] OR “diagnostic error”[tiab] OR “Diagnostic Errors”[MeSH]) AND (“Child”[Mesh] OR “Adolescent”[Mesh] OR child*[tiab] OR adolescen*[tiab] OR pediatric*[tiab])	‘alimentary allergy’ OR (alimentary AND (‘allergy’/exp OR allergy))) OR ‘atopic dermatitis’ OR ‘food allergy’) AND (‘digestive tract endoscopy’ OR ‘prick test’ OR ‘immunoglobulin e’) AND (‘diagnostic accuracy’ OR ‘diagnostic error’)” AND (‘child’/exp OR ‘adolescent’/exp OR child*:ti,ab OR adolescen*:ti,ab OR pediatric*:ti,ab)	#1 MeSH descriptor: [Food Hypersensitivity] explode all trees #2 MeSH descriptor: [Atopic Dermatitis] explode all trees #3 (“Food Allergy” OR “alimentary allergy” OR “atopic dermatitis”):ti,ab,kw #4 #1 OR #2 OR #3 #5 MeSH descriptor: [Endoscopy, Gastrointestinal] explode all trees #6 MeSH descriptor: [Skin Tests] explode all trees #7 MeSH descriptor: [Immunoglobulin E] explode all trees #8 (“digestive tract endoscopy” OR “prick test” OR IgE):ti,ab,kw #9 #5 OR #6 OR #7 OR #8 #10 MeSH descriptor: [Sensitivity and Specificity] explode all trees #11 MeSH descriptor: [Diagnostic Errors] explode all trees #12 (“diagnostic accuracy” OR “diagnostic error”):ti,ab,kw #13 #10 OR #11 OR #12 #14 MeSH descriptor: [Child] explode all trees #15 MeSH descriptor: [Adolescent] explode all trees #16 (child* OR adolescen* OR pediatric*):ti,ab,kw #17 #14 OR #15 OR #16 #18 #4 AND #9 AND #13 AND #17
**Orticaria and Angioedema**		
Research Question (PICO) 1		
(“Urticaria”[Mesh] OR “Angioedema”[Mesh] OR “Chronic Urticaria”[Mesh] OR “Chronic Inducible Urticaria”[Mesh]) AND (“Ambulatory Care”[Mesh] OR “Day Care, Medical”[Mesh] OR “home visit*” OR “outpatient*”) AND (“Medication Adherence”[Mesh] OR “Disease Management”[Mesh] OR “medication compliance” OR “drug compliance” OR adherence OR “appropriate use” OR “patient compliance” OR “quality of life”) AND (“Child”[MeSH] OR “Adolescent”[MeSH] OR child[tiab] OR adolescent[tiab] OR pediatric[tiab] OR paediatric[tiab])	(“Urticaria”[Mesh] OR “Angioedema”[Mesh] OR “Chronic Urticaria”[Mesh] OR “Chronic Inducible Urticaria”[Mesh]) AND (“Ambulatory Care”[Mesh] OR “Day Care, Medical”[Mesh] OR “home visit*” OR “outpatient*”) AND (“Medication Adherence”[Mesh] OR “Disease Management”[Mesh] OR “medication compliance” OR “drug compliance” OR adherence OR “appropriate use” OR “patient compliance” OR “quality of life”) AND (“Child”[MeSH] OR “Adolescent”[MeSH] OR child[tiab] OR adolescent[tiab] OR pediatric[tiab] OR paediatric[tiab])	(“Urticaria”[Mesh] OR “Angioedema”[Mesh] OR “Chronic Urticaria”[Mesh] OR “Chronic Inducible Urticaria”[Mesh]) AND (“Ambulatory Care”[Mesh] OR “Day Care, Medical”[Mesh] OR “home visit*” OR “outpatient*”) AND (“Medication Adherence”[Mesh] OR “Disease Management”[Mesh] OR “medication compliance” OR “drug compliance” OR adherence OR “appropriate use” OR “patient compliance” OR “quality of life”) AND (“Child”[MeSH] OR “Adolescent”[MeSH] OR child[tiab] OR adolescent[tiab] OR pediatric[tiab] OR paediatric[tiab])
Research Question (PICO) 2		
(“Urticaria”[Mesh] OR “Angioedema”[Mesh]) AND (“Skin Tests”[Mesh] OR “Medical History Taking”[Mesh] OR “Immunoglobulin E”[Mesh]) AND “Risk Factors”[Mesh]) AND (“Child”[MeSH] OR “Adolescent”[MeSH] OR child[tiab] OR adolescent[tiab] OR pediatric[tiab] OR paediatric[tiab])	((‘urticaria’ OR ‘acute urticaria’ OR ‘angioneurotic edema’) AND (‘prick test’ OR ‘immunoglobulin e’ OR ‘anamnesis’ OR ‘risk factor’)) AND ‘diagnostic accuracy’ AND ([adolescent]/lim OR [child]/lim)	#1 MeSH descriptor: [Urticaria] explode all trees #2 MeSH descriptor: [Angioedema] explode all trees #3 #1 OR #2 #4 MeSH descriptor: [Skin Tests] explode all trees #5 MeSH descriptor: [Medical History Taking] explode all trees #6 MeSH descriptor: [Immunoglobulin E] explode all trees #7 #4 OR #5 OR #6 #8 MeSH descriptor: [Risk Factors] explode all trees #9 MeSH descriptor: [Child] explode all trees #10 MeSH descriptor: [Adolescent] explode all trees #11 (child OR adolescent OR pediatric OR paediatric):ti,ab,kw #12 #9 OR #10 OR #11 #13 #3 AND #7 AND #8 AND #12
Research Question (PICO) 3		
(“Urticaria”[MeSH] OR “Chronic Urticaria”[MeSH] OR “chronic urticaria”[tiab]) AND (“Medical History Taking”[MeSH] OR anamnes*[tiab] OR “history-based”[tiab] OR “targeted diagnosis”[tiab] OR “focused diagnostic approach”[tiab] OR “clinical evaluation”[tiab]) AND (“Diagnostic Tests, Routine”[MeSH] OR “Allergy Tests”[MeSH] OR “Autoimmune Diseases/diagnosis”[MeSH] OR “diagnostic workup”[tiab] OR “extensive testing”[tiab] OR “unnecessary test*”[tiab] OR “non-targeted test*”[tiab]) AND (“Health Care Costs”[MeSH] OR “Cost-Benefit Analysis”[MeSH] OR “Health Care Utilization”[MeSH] OR “Diagnostic Errors”[MeSH] OR “Appropriateness of Care”[MeSH] OR cost*[tiab] OR “diagnostic appropriateness”[tiab] OR “unnecessary testing”[tiab] OR “overdiagnosis”[tiab]) AND (“Child”[MeSH] OR “Adolescent”[MeSH] OR child[tiab] OR adolescent[tiab] OR pediatric[tiab] OR paediatric[tiab])	(‘urticaria’/exp OR ‘chronic urticaria’/exp OR ‘chronic urticaria’:ti,ab) AND (‘child’/exp OR ‘adolescent’/exp OR child*:ti,ab OR adolescent*:ti,ab OR pediatric*:ti,ab OR paediatric*:ti,ab) AND (‘medical history’/exp OR anamnes*:ti,ab OR ‘history based’:ti,ab OR ‘targeted diagnosis’:ti,ab OR ‘focused diagnostic approach’:ti,ab OR ‘clinical evaluation’:ti,ab) AND (‘diagnostic test’/exp OR ‘allergy test’/exp OR ‘autoimmune disease diagnosis’/exp OR ‘diagnostic workup’:ti,ab OR ‘extensive testing’:ti,ab OR ‘non targeted test*’:ti,ab OR ‘unnecessary test*’:ti,ab) AND (‘health care cost’/exp OR ‘cost effectiveness analysis’/exp OR ‘health care utilization’/exp OR ‘diagnostic error’/exp OR ‘health care quality’/exp OR cost*:ti,ab OR ‘diagnostic appropriateness’:ti,ab OR ‘unnecessary testing’:ti,abOR overdiagnosis:ti,ab)	#1 MeSH descriptor: [Urticaria] explode all trees #2 MeSH descriptor: [Chronic Urticaria] explode all trees #3 “chronic urticaria”:ti,ab,kw #4 #1 OR #2 OR #3 #5 MeSH descriptor: [Medical History Taking] explode all trees #6 (anamnes* OR “history-based” OR “targeted diagnosis” OR “focused diagnostic approach” OR “clinical evaluation”):ti,ab,kw #7 #5 OR #6 #8 MeSH descriptor: [Diagnostic Tests, Routine] explode all trees #9 MeSH descriptor: [Allergy Tests] explode all trees #10 MeSH descriptor: [Autoimmune Diseases] explode all trees #11 (“diagnostic workup” OR “extensive testing” OR “unnecessary test*” OR “non-targeted test*”):ti,ab,kw #12 #8 OR #9 OR #10 OR #11 #13 MeSH descriptor: [Health Care Costs] explode all trees #14 MeSH descriptor: [Cost-Benefit Analysis] explode all trees #15 MeSH descriptor: [Health Care Utilization] explode all trees #16 MeSH descriptor: [Diagnostic Errors] explode all trees #17 MeSH descriptor: [Appropriateness of Care] explode all trees #18 (cost* OR “diagnostic appropriateness” OR “unnecessary testing” OR overdiagnosis):ti,ab,kw #19 #13 OR #14 OR #15 OR #16 OR #17 OR #18 #20 MeSH descriptor: [Child] explode all trees #21 MeSH descriptor: [Adolescent] explode all trees #22 (child OR adolescent OR pediatric OR paediatric):ti,ab,kw #23 #20 OR #21 OR #22 #24 #4 AND #7 AND #12 AND #19 AND #23
Research Question (PICO) 4		
“Urticaria”[Mesh] NOT “Angioedema”[Mesh]) AND AND (“Ambulatory Care”[Mesh] OR “Day Care, Medical”[Mesh] OR “home visit*” OR “outpatient*”) AND (“Medication Adherence”[Mesh] OR “Disease Management”[Mesh] OR “medication compliance” OR “drug compliance” OR adherence OR “appropriate use” OR “patient compliance” AND (“Child”[MeSH] OR “Adolescent”[MeSH] OR child[tiab] OR adolescent[tiab] OR pediatric[tiab] OR paediatric[tiab])	(‘angioneurotic edema’ NOT ‘urticaria’) AND (‘outpatient’ OR ‘medical assessment’ OR ‘immunologist’ OR ((‘ambulatory care’ OR ‘outpatient’ OR ‘home visit’ OR ‘day care’) AND (‘medication compliance’ OR ‘disease management’ OR ‘patient compliance’))) AND ‘disease management’ AND (‘child’/exp OR ‘adolescent’/exp OR child*:ti,ab OR adolescent*:ti,ab OR pediatric*:ti,ab OR paediatric*:ti,ab)	#1 MeSH descriptor: [Urticaria] explode all trees #2 MeSH descriptor: [Angioedema] explode all trees #3 #1 NOT #2 #4 MeSH descriptor: [Ambulatory Care] explode all trees #5 MeSH descriptor: [Day Care, Medical] explode all trees #6 (“home visit*” OR outpatient*):ti,ab,kw #7 #4 OR #5 OR #6 #8 MeSH descriptor: [Medication Adherence] explode all trees #9 MeSH descriptor: [Disease Management] explode all trees #10 (“medication compliance” OR “drug compliance” OR adherence OR “appropriate use” OR “patient compliance”):ti,ab,kw #11 #8 OR #9 OR #10 #12 MeSH descriptor: [Child] explode all trees #13 MeSH descriptor: [Adolescent] explode all trees #14 (child OR adolescent OR pediatric OR paediatric):ti,ab,kw #15 #12 OR #13 OR #14 #16 #3 AND #7 AND #11 AND #15
Research Question (PICO) 5		
(“Angioedema”[MeSH] OR angioedema[tiab]) AND (recurrent*[tiab] OR “recurrent angioedema”[tiab]) AND “Child”[MeSH] OR “Adolescent”[MeSH] OR child*[tiab] OR adolescent*[tiab] OR pediatric*[tiab] OR paediatric*[tiab]) AND (“C1 Esterase Inhibitor”[MeSH] OR “C1 Inhibitor”[tiab] OR “C1-INH”[tiab] OR “Complement C4”[MeSH] OR C4[tiab] OR “Complement C1q”[MeSH] OR C1q[tiab]) AND (“Hereditary Angioedema”[MeSH] OR “hereditary angioedema”[tiab] OR “acquired angioedema”[tiab] OR “bradykinin-mediated angioedema”[tiab] OR “allergic angioedema”[tiab]) AND (“Diagnosis”[MeSH] OR diagnos*[tiab] OR “differential diagnosis”[MeSH] OR “clinical observation”[tiab] OR “watchful waiting”[tiab])	(‘angioedema’/exp OR angioedema:ti,ab) AND (recurrent*:ti,ab OR ‘recurrent angioedema’:ti,ab) AND (‘child’/exp OR ‘adolescent’/exp OR child*:ti,ab OR adolescent*:ti,ab OR pediatric*:ti,ab OR paediatric*:ti,ab) AND (‘c1 esterase inhibitor’/exp OR ‘c1 inhibitor’:ti,ab OR ‘c1-inh’:ti,ab OR ‘complement c4’/exp OR c4:ti,ab OR ‘complement c1q’/exp OR c1q:ti,ab) AND (‘hereditary angioedema’/exp OR ‘hereditary angioedema’:ti,ab OR ‘acquired angioedema’:ti,ab OR ‘bradykinin mediated angioedema’:ti,ab OR ‘allergic angioedema’:ti,ab) AND (‘diagnosis’/exp OR ‘differential diagnosis’/exp OR diagnos*:ti,ab OR ‘clinical observation’:ti,ab OR ‘watchful waiting’:ti,ab)	#1 MeSH descriptor: [Angioedema] explode all trees #2 angioedema:ti,ab,kw #3 #1 OR #2 #4 (recurrent* OR “recurrent angioedema”):ti,ab,kw #5 MeSH descriptor: [Child] explode all trees #6 MeSH descriptor: [Adolescent] explode all trees #7 (child* OR adolescent* OR pediatric* OR paediatric*):ti,ab,kw #8 #5 OR #6 OR #7 #9 MeSH descriptor: [C1 Esterase Inhibitor] explode all trees #10 MeSH descriptor: [Complement C4] explode all trees #11 MeSH descriptor: [Complement C1q] explode all trees #12 (“C1 Inhibitor” OR “C1-INH” OR C4 OR C1q):ti,ab,kw #13 #9 OR #10 OR #11 OR #12 #14 MeSH descriptor: [Hereditary Angioedema] explode all trees #15 (“hereditary angioedema” OR “acquired angioedema” OR “bradykinin-mediated angioedema” OR “allergic angioedema”):ti,ab,kw #16 #14 OR #15 #17 MeSH descriptor: [Diagnosis] explode all trees #18 MeSH descriptor: [Differential Diagnosis] explode all trees #19 (diagnos* OR “clinical observation” OR “watchful waiting”):ti,ab,kw #20 #17 OR #18 OR #19 #21 #3 AND #4 AND #8 AND #13 AND #16 AND #20
**Atopic Dermatitis**		
Research Question (PICO) 1, 2, 3		
“Dermatitis, Atopic”[Mesh] AND (“Allergy and Immunology”[Mesh] OR “Immunologic Tests”[Mesh] OR “Ambulatory Care”[Mesh] OR “Day Care, Medical”[Mesh] OR “home visit*” OR “outpatient*”) AND (“Diagnosis”[Mesh] OR “Diagnosis, Differential”[Mesh] OR “Recurrence”[Mesh] OR “Missed Diagnosis”[Mesh] OR “Medication Adherence”[Mesh] OR “Disease Management”[Mesh] OR “medication compliance” OR “drug compliance” OR adherence OR “appropriate use” OR “patient compliance”) AND (“Child”[MeSH] OR “Adolescent”[MeSH] OR child[tiab] OR adolescent[tiab] OR pediatric[tiab] OR paediatric[tiab])	‘atopic dermatitis’ AND (‘immunology’ OR ‘immunological procedures’) AND (‘ambulatory care’ OR ‘outpatient’ OR ‘day care’ OR ‘home visit’ OR ‘outpatient care’) AND (‘diagnosis’ OR ‘diagnostic procedure’ OR ‘differential diagnosis’ OR ‘missed diagnosis’ OR ‘disease management’ OR ‘medication compliance’ OR ‘quality of life’ OR ‘patient compliance’) AND ([adolescent]/lim OR [child]/lim)	#1 MeSH descriptor: [Dermatitis, Atopic] explode all trees #2 MeSH descriptor: [Allergy and Immunology] explode all trees #3 MeSH descriptor: [Immunologic Tests] explode all trees #4 MeSH descriptor: [Ambulatory Care] explode all trees #5 MeSH descriptor: [Day Care, Medical] explode all trees #6 (“home visit*” OR outpatient*):ti,ab,kw #7 #2 OR #3 OR #4 OR #5 OR #6 #8 MeSH descriptor: [Diagnosis] explode all trees #9 MeSH descriptor: [Differential Diagnosis] explode all trees #10 MeSH descriptor: [Recurrence] explode all trees #11 MeSH descriptor: [Diagnostic Errors] explode all trees #12 MeSH descriptor: [Medication Adherence] explode all trees #13 MeSH descriptor: [Disease Management] explode all trees #14 (“medication compliance” OR “drug compliance” OR adherence OR “appropriate use” OR “patient compliance”):ti,ab,kw #15 #8 OR #9 OR #10 OR #11 OR #12 OR #13 OR #14 #16 MeSH descriptor: [Child] explode all trees #17 MeSH descriptor: [Adolescent] explode all trees #18 (child OR adolescent OR pediatric OR paediatric):ti,ab,kw #19 #16 OR #17 OR #18 #20 #1 AND #7 AND #15 AND #19
Research Question (PICO) 4		
(“Dermatitis, Atopic”[Mesh] OR “atopic dermatitis”[tiab] OR eczema[tiab]) AND (“Child”[Mesh] OR “Adolescent”[Mesh] OR child*[tiab] OR adolescen*[tiab] OR pediatric*[tiab]) AND (“Health Education”[Mesh] OR “education program”[tiab] OR “parent education”[tiab] OR “educational intervention”[tiab]) AND (“Treatment Adherence and Compliance”[Mesh] OR adherence[tiab] OR compliance[tiab]) AND (“Disease Management”[Mesh] OR “disease control”[tiab] OR “symptom control”[tiab]) (“Child”[MeSH] OR “Adolescent”[MeSH] OR child[tiab] OR adolescent[tiab] OR pediatric[tiab] OR paediatric[tiab])	(‘atopic dermatitis’/exp OR ‘atopic dermatitis’:ti,ab OR eczema:ti,ab) AND (‘child’/exp OR ‘adolescent’/exp OR child*:ti,ab OR adolescen*:ti,ab OR pediatric*:ti,ab) AND (‘health education’/exp OR ‘educational program’:ti,ab OR ‘parent education’:ti,ab OR ‘educational intervention’:ti,ab) AND (‘patient compliance’/exp OR adherence:ti,ab OR compliance:ti,ab) AND (‘disease control’/exp OR ‘disease management’/exp OR ‘symptom control’:ti,ab)AND ([adolescent]/lim OR [child]/lim)	#1 MeSH descriptor: [Dermatitis, Atopic] explode all trees #2 (“atopic dermatitis” OR eczema):ti,ab,kw #3 #1 OR #2 #4 MeSH descriptor: [Child] explode all trees #5 MeSH descriptor: [Adolescent] explode all trees #6 (child* OR adolescen* OR pediatric*):ti,ab,kw #7 #4 OR #5 OR #6 #8 MeSH descriptor: [Health Education] explode all trees #9 (“education program” OR “parent education” OR “educational intervention”):ti,ab,kw #10 #8 OR #9 #11 MeSH descriptor: [Treatment Adherence and Compliance] explode all trees #12 (adherence OR compliance):ti,ab,kw #13 #11 OR #12 #14 MeSH descriptor: [Disease Management] explode all trees #15 (“disease control” OR “symptom control”):ti,ab,kw #16 #14 OR #15 #17 (child OR adolescent OR pediatric OR paediatric):ti,ab,kw #18 #4 OR #5 OR #17 #19 #3 AND #7 AND #10 AND #13 AND #16 AND #18

**Table A2:** Summary of original studies included in the analyses.

Author	Objectives	Study Population and Design (General Characteristics)	Sample Size (No.)	Outcome	Key Results (Quantitative Findings)	Main Findings	Limitations	PICO
**IgE-Mediated and Non-IgE Mediated Food Allergy and Eosinophilic Gastrointestinal Disease**								
Miceli Sopo et al. 2019 [26]	To assess whether an increasing number of adverse reaction episodes (AREs) to a specific food is associated with a positive oral food challenge (OFC) result.	Children (mean age, 1.8 ± 2.7 years; range, 0.04–12.8 years) with a history of non-anaphylactic adverse reactions to food, a positive skin prick test to the suspected food allergen, and an oral food challenge (OFC) performed within 12 months of their most recent reaction.	180	Number of adverse reaction episodes per child (1, 2, 3, or ≥4); oral food challenge (OFC) outcome (positive or negative, corresponding to the presence or absence of IgE-mediated food allergy).	Adverse reactions: 93 of 180 children (52%) experienced one adverse reaction episode, 49 (27%) experienced two episodes, 24 (13%) experienced three episodes, and 14 (8%) experienced ≥ 4 episodes. OFC outcome: 94 of 180 oral food challenges (52%) were positive. A significant positive association was observed between the number of adverse reaction episodes (AREs) and a positive OFC outcome (OR, 1.56 per additional episode; 95% CI, 1.16–2.09; *p* = 0.003). A positive predictive value (PPV) of 100% was observed among children with ≥5 adverse reaction episodes (AREs).	The number of adverse reaction episodes (AREs) appears to be an important predictor of food allergy diagnosis and may enhance the predictive performance of currently available diagnostic tests. Furthermore, it may support the development of simple, clinically applicable prediction rules as an alternative to oral food challenge (OFC).	Retrospective study design. Limited sample size, particularly among children with a high number of adverse reaction episodes (especially ≥ 5 AREs). Only non-anaphylactic reactions were included; therefore, the findings may not be generalizable to children with severe food allergy. The single-country setting (Italy) and the specific inclusion criteria (positive skin prick test and OFC performed within 12 months of the most recent reaction) may limit the generalizability of the findings.	1, 2
Jacob et al. 2023 [30]	To identify predictors of positive and negative oral food challenge (OFC) outcomes in Australian children and adolescents.	Pediatric patients aged 3 months to 17 years who underwent an oral food challenge (OFC) at a tertiary allergy service over a 5-year period.	465	Incidence of allergic reactions during oral food challenges (OFCs). Identification of clinical predictors of positive and negative OFC outcomes.	Overall, 12.3% (56/456) of oral food challenges (OFCs) resulted in an allergic reaction. Atopic dermatitis was significantly associated with an increased likelihood of an allergic reaction during OFCs (odds ratio [OR], 1.99).	Larger skin prick test (SPT) wheal size and a history of anaphylaxis to the challenge food were strong predictors of a positive OFC outcome. No significant association was observed between atopic dermatitis and reactions to soy or shrimp.	Single-center study with a limited sample size. The findings may not be generalizable to broader populations. Further large-scale, multicenter studies are needed to validate these findings.	1, 2
Parker et al. 2024 [109]	To evaluate the association between longitudinal levels of peanut- and Ara h 2-specific IgE and IgG_4_, as well as the IgG_4_/IgE ratio, and the outcome of peanut oral food challenges (OFCs) in children and adolescents followed over time.	One-year-old children with confirmed peanut allergy (*n* = 156) from the HealthNuts cohort (*n* = 5276) were prospectively followed at 4, 6, and 10 years of age using questionnaires, skin prick tests, oral food challenges (OFCs), and measurements of total plasma IgE, peanut- and Ara h 2-specific IgE, and peanut- and Ara h 2-specific IgG_4_.	156	Resolution of peanut allergy by 10 years of age, confirmed by oral food challenge (OFC). Longitudinal measurements of peanut- and Ara h 2-specific IgE and IgG_4_. Longitudinal assessment of the IgG_4_/IgE ratio.	By 10 years of age, 33.9% of children (95% CI, 25.3–43.3%) had achieved resolution of peanut allergy, with most cases (97.4%) resolving by 6 years of age. Decreasing Ara h 2-specific IgE levels (*p* = 0.011), increasing Ara h 2- and peanut-specific IgG_4_ levels (*p* < 0.001 and *p* = 0.011, respectively), and an increasing IgG_4_/IgE ratio (*p* < 0.001) were associated with allergy resolution. Peanut-specific IgE levels were highest at 1 year of age.	Approximately one-third of childhood peanut allergies resolved by 10 years of age. However, biomarker levels at diagnosis were not strongly associated with the natural history of peanut allergy.	Biomarker levels at diagnosis were not strong predictors of allergy resolution. The findings may not be generalizable beyond the specific cohort studied. Potential confounding factors during the 10-year follow-up period were not fully accounted for.	2
Goldberg et al. 2024 [110]	To validate the NUT CRACKER diagnostic algorithm for cashew and pistachio allergy and to identify biomarkers associated with allergy severity.	A total of 125 children (age range, 5.9–11.2 years; mean age, 7.8 years) with suspected tree nut allergy were prospectively evaluated. An additional cohort increased the total study population to 187 participants for the severity analysis.	187	Validation of allergy status by oral food challenge (OFC). Assessment of the diagnostic performance of skin prick testing (SPT), the basophil activation test (BAT), and Ana o 3-specific IgE. Evaluation of the algorithm’s effectiveness in reducing the number of diagnostic OFCs required.	The NUT CRACKER algorithm reduced the overall number of OFCs by 72%, with a positive predictive value (PPV) of 93% and a negative predictive value (NPV) of 99%. SPT reactivity, BAT responses, Ana o 3-specific IgE levels, and the incidence of abdominal pain during OFCs were significantly higher in children allergic to both cashew and pistachio than in those allergic only to cashew. All three diagnostic tests showed a significant inverse correlation with the eliciting dose during positive cashew OFCs. Ana o 3-specific IgE demonstrated a sensitivity > 90% and a specificity > 95% for the diagnosis of cashew allergy.	The NUT CRACKER diagnostic algorithm was successfully validated and substantially reduced the number of diagnostic OFCs required. Biomarkers associated with severe allergic phenotypes may help guide oral immunotherapy protocols and improve the risk–benefit balance for patients.	Potential risk of false-positive results, particularly among patients with hazelnut allergy. The study population may not be representative of all demographic groups, limiting the generalizability of the findings. Further studies are needed to evaluate the applicability of the algorithm in broader clinical settings.	2
Diaz et al. 2022 [32]	To evaluate the diagnostic utility of skin prick tests (SPTs) and serum allergen-specific IgE (sIgE) measurements for the diagnosis of cow’s milk protein allergy (CMPA) in a pediatric population.	Pediatric patients (mean age, 9.1 months; median age, 5 months) evaluated at the Allergy Unit of a tertiary pediatric hospital between 2015 and 2018.	239	Sensitivity and specificity of SPTs and sIgE for cow’s milk, α-lactalbumin, β-lactoglobulin, and casein. Diagnostic accuracy of individual tests and their combinations, using the oral food challenge (OFC) as the reference standard.	Casein SPT showed the highest specificity (96.7%; 95% CI, 90.8–99.3%). The combination of SPT and sIgE for all four allergens yielded the highest sensitivity (55.3%; 95% CI, 45.7–64.6%). OFCs were performed in hospital in 54.8% of cases, as home-based rechallenges in 35.5%, and through maternal dietary reintroduction in 9.6%.	Although SPTs and sIgE demonstrated limited sensitivity and negative predictive value (NPV), they may still be useful in supporting clinical decision-making in the study population.	The retrospective study design may have introduced selection bias. The single-center setting may limit the generalizability of the findings. The moderate sensitivity of SPTs and sIgE indicates that these tests should be interpreted in conjunction with the clinical history and OFC results	2
Chauveau et al. 2016 [25]	To assess the agreement between skin prick tests (SPTs) and allergen-specific IgE (sIgE) measurements for the diagnosis of sensitization to aeroallergens and food allergens during early childhood.	French children (0–6 years of age) from the PASTURE study, assessed at 1, 4.5, and 6 years of age.	204	Agreement between SPTs and sIgE for aeroallergens and food allergens. Association between SPT positivity and the development of atopic dermatitis.	Poor agreement was observed between SPTs and sIgE, except for perennial aeroallergens at 6 years of age using an sIgE cutoff > 0.7 IU/mL (κ = 0.69). The prevalence of positive SPTs increased with age. Positive SPTs at 1 year of age were predictive of the subsequent development of atopic dermatitis during follow-up.	SPTs showed poor agreement with serum allergen-specific IgE measurements during early childhood, suggesting that both tests should be used in combination. Cutaneous allergic reactivity increased with age and was transient at 1 year of age, where it was associated with the subsequent development of atopic dermatitis.	The study was conducted in a specific cohort, which may limit the generalizability of the findings. Only a limited panel of allergens was evaluated; broader allergen panels were not assessed.	1, 2
Zivanovic et al. 2017 [31]	To evaluate the diagnostic performance of skin prick tests (SPTs) using commercial extracts and fresh foods, allergen-specific IgE (sIgE), and open oral food challenges (OFCs) in children with suspected food allergy.	Children aged 2 months to 6 years with suspected IgE-mediated food allergy.	570	Diagnostic accuracy of skin prick tests (SPTs) and allergen-specific IgE (sIgE), and their correlation with oral food challenge (OFC) outcomes.	The sensitivity of SPTs performed with commercial extracts was low for all foods tested (3–35%). In contrast, fresh food skin prick tests (FFSPTs) showed excellent sensitivity (50–100%) and were significantly correlated with open OFC outcomes (*p* < 0.001).	Fresh food skin prick testing was more effective than commercial extracts in detecting sensitization. In combination with sIgE levels above Class 3, FFSPTs may help predict clinical reactivity and reduce the need for potentially hazardous oral food challenges.	Retrospective study design. Lack of double-blind, placebo-controlled food challenges (DBPCFCs). Single-center study.	2
Choi B et al. 2015 [41]	To evaluate the clinical, endoscopic, laboratory, and histopathological characteristics of eosinophilic gastroenteritis (EGE) in Korean infants (<1 year) and children (>1 year).	Infants (<1 year) and children (>1 year), including 9 males and 13 females; 13 patients had histologically confirmed EGE (hEGE), and 9 had probable EGE (pEGE).	22	Endoscopic and histopathological findings. Allergen sensitization profile.	The most common presenting symptoms were hematemesis (53.8%), abdominal pain (23.1%), vomiting (15.4%), melena (15.4%), and lower-extremity edema (15.4%). Among patients with histologically confirmed EGE (hEGE), allergen-specific IgE was positive in 2 of 5 patients (40%). Among patients with probable EGE (pEGE), allergen-specific IgE was positive in 3 of 6 patients (60%). Three patients (21%) had normal endoscopic findings. Infants had significantly higher gastric eosinophil counts than older children (32.6 vs. 3.8 eosinophils/high-power field [HPF]; *p* = 0.008).	Because the clinical presentation is nonspecific and no specific laboratory test is available, the diagnosis of eosinophilic gastroenteritis relies on histopathological examination.	Small sample size.	3, 4
Votto et al. 2023 [100]	To analyze and identify clinical factors and complications associated with a longer diagnostic delay in pediatric patients with eosinophilic gastrointestinal disorders (EGIDs).	Sixty pediatric patients (6–11 years of age) with EGIDs, including 39 (65%) with eosinophilic esophagitis (EoE) and 21 (35%) with non-esophageal EGIDs.	60	Diagnostic delay and its association with clinical characteristics and complications.	The median diagnostic delay was 12 months (IQR, 12–69 months) among patients with non-esophageal EGIDs and 12 months (IQR, 4–24 months) among those with EoE. Among patients with EoE, failure to thrive (FTT) and feeding difficulties were associated with a significantly longer diagnostic delay compared with children without growth impairment or feeding problems (*p* = 0.02 and *p* = 0.05, respectively).	Delayed diagnosis was strongly associated with impaired growth in children. A multidisciplinary pediatric assessment is essential for the early identification of suspected cases.	Small sample size.	3
Del Mar Vasquez et al. 2023 [39]	To describe the clinical and demographic characteristics of a pediatric population with eosinophilic esophagitis (EoE).	Thirty-five children (5–12 years of age), including 21 females (60%), diagnosed with EoE.	35	Clinical presentation. Skin prick test (SPT) and allergen-specific IgE (sIgE) results. Endoscopic and histopathological findings	The most common clinical manifestations included abdominal pain, gastroesophageal reflux disease (GERD), choking episodes, vomiting, nausea, dysphagia, and food bolus impaction. SPTs were performed in 32 patients (91.4%): 11 (34.4%) were sensitized to aeroallergens, 3 (9.4%) to food allergens, 10 (31.2%) showed mixed sensitization, and 8 (25.0%) had negative results. Allergen-specific IgE measurements were available for 8 patients (25.0%), of whom 4 (50.0%) tested positive. The median peak eosinophil count was 42 eosinophils/high-power field (HPF).	Children with EoE showed a high prevalence of concomitant allergic diseases, particularly allergic rhinitis, as well as allergic sensitization, especially to house dust mites.	Small sample size.	4
Azzano et al. 2020 [38]	To evaluate the long-term clinical and histopathological outcomes of eosinophilic esophagitis (EoE) in children, with particular emphasis on allergy management.	A total of 108 patients aged 0–18 years with a diagnosis of EoE, including 86 males (79.6%) and 22 females (20.4%).	108	Endoscopic and histopathological findings. Allergen sensitization profile.	Peak eosinophil density ranged from 15 to >100 eosinophils/high-power field (HPF). Skin prick tests (SPTs) were positive in 60.6% of patients, most commonly to lentils, tree nuts, fish, wheat, egg, soy, chicken, peanuts, and cow’s milk (9.3%). Allergen-specific IgE was positive in 54.4% of patients, most frequently to egg, lentils, wheat, cow’s milk, soy, tree nuts, and chicken.	An allergic background was common (70.3%), and 80% of patients benefited from allergy testing. Allergy evaluation proved particularly valuable in guiding treatment, as elimination diets were effective in approximately 60% of patients.	Single-center study.	4
Erwin et al. 2015 [40]	To further characterize food allergen-specific IgE sensitization, with particular emphasis on foods that may play a role in eosinophilic esophagitis (EoE).	Fifty-one pediatric patients with EoE.	51	Skin prick testing (SPT) and serum allergen-specific IgE measurements for aeroallergens, food extracts, and molecular allergen components.	Food SPTs were positive in 5.9% of children. Serum allergen-specific IgE testing identified a higher prevalence of food sensitization than SPT. Cow’s milk sensitization was primarily directed against the minor allergens Bos d 4 and Bos d 5.	Food sensitization identified by serum allergen-specific IgE measurements is consistent with existing evidence indicating that foods such as cow’s milk and wheat play a significant role in the inflammatory process underlying EoE.	Patients were not classified according to their responsiveness to proton pump inhibitor (PPI) therapy.	4
Erwin et al. 2017 [106]	To evaluate the ability of food allergen-specific IgE antibodies to predict the presence of esophageal eosinophilia.	A total of 144 children who underwent esophageal biopsy and serum allergen-specific IgE testing for cow’s milk, egg, wheat, soy, and peanut.	144	Development of a predictive model for eosinophilic esophagitis (EoE).	The likelihood of esophageal eosinophilia increased with the number of positive food allergen-specific IgE tests and was higher in male patients. The predicted probability of esophageal eosinophilia ranged from 12% in females with no positive food-specific IgE tests to 86% in males with four or five positive food-specific IgE tests.	Assessment of food allergen-specific IgE in patients with nonspecific gastrointestinal symptoms may facilitate earlier diagnosis and treatment of esophageal eosinophilia.	No differences were observed between patients receiving proton pump inhibitor (PPI) therapy and those not receiving PPI therapy.	4
Votto et al. 2022 [42]	To evaluate the clinical characteristics of pediatric patients with eosinophilic gastrointestinal disorders (EGIDs) managed at a tertiary pediatric center.	A total of 112 pediatric patients (mean age, 9.3 ± 4.8 years), of whom 75.8% were male.	112	Assessment of the diagnostic trends of EGIDs. Assessment of allergic comorbidities in pediatric patients with EGIDs	An increasing number of EGID diagnoses was observed during the final two years of the study period (annual increase, 30.5%), with a slightly higher prevalence of non-esophageal EGIDs than eosinophilic esophagitis (EoE) (5.1% vs. 4.4%). A comprehensive allergy evaluation, including skin prick testing (SPT) and serum allergen-specific IgE measurements, was performed in 62 patients (55.4%), including 31 children with EoE and 31 with non-esophageal EGIDs. Although the difference was not statistically significant, peripheral eosinophilia and serum total IgE levels ≥ 100 kU/L were more common in children with EoE than in those with non-esophageal EGIDs.	Approximately 30% of patients had allergic comorbidities, which were more common among children with EoE.	Retrospective study design. Lack of standardized diagnostic guidelines and validated pathological cut-off values for intestinal eosinophil counts.	4
**Urticaria and Angioedema**								
Kosmeri C et al. 2019 [57]	To evaluate the diagnostic utility of serum tryptase levels as a biomarker of pediatric anaphylaxis.	Children with anaphylaxis who underwent serum tryptase measurements during and after the allergic reaction.	68	Diagnostic performance of serum tryptase levels as a biomarker of pediatric anaphylaxis.	During the allergic reaction, 19.2% of children had serum tryptase levels ≥ 11.4 μg/L. Tryptase levels exceeding the diagnostic threshold (baseline tryptase × 1.2 + 2 ng/mL) were observed in 85.7% of severe reactions, 54.2% of moderate reactions, and 69.2% of mild reactions. Among the 68 children with both acute and post-reaction measurements, the mean serum tryptase concentration was 9.9 μg/L during the reaction and 3.6 μg/L after the reaction, corresponding to a mean difference of 6.3 μg/L.	Severe reactions were more frequently associated with elevated serum tryptase levels. However, many children with clinically evident anaphylaxis had normal tryptase concentrations, indicating the limited sensitivity of this biomarker. Serum tryptase levels were generally higher during the reaction than at baseline, supporting their role as a marker of mast cell activation during anaphylaxis.	Only 68 patients had both acute and post-reaction tryptase measurements. Single-center study.	
Yilmaz E. et al. 2017 [53]	To evaluate the clinical characteristics of chronic spontaneous urticaria (CSU) in children and identify risk factors for disease persistence, with particular focus on the association between disease activity, assessed by UAS7, and symptom duration.	Children aged 1–17 years with chronic spontaneous urticaria, diagnosed between 1992 and 2015 and followed at the Department of Pediatric Allergy, Hacettepe University, Ankara, Turkey.	222	Prevalence of autoimmune diseases and autoimmune markers in children with chronic spontaneous urticaria.	Celiac disease was diagnosed in 1 child (2.0%). Elevated anti-thyroid peroxidase antibodies (anti-TPO) were detected in 4 children (8.1%). Antinuclear antibodies (ANA; titer 1:160) were positive in 3 children, with negative anti-dsDNA and extractable nuclear antigen (ENA) antibodies. Elevated IgE levels were observed in 8 children. The autologous serum skin test (ASST) was negative in all tested patients.	Autoimmune diseases were uncommon among children with CSU. However, the detection of subclinical autoimmune markers, including anti-TPO antibodies and ANA, in some children suggests that CSU may be associated with autoimmune predisposition, even in the absence of systemic symptoms. The case of celiac disease supports the possibility that urticaria may represent an isolated manifestation of latent autoimmune disease and that a gluten-free diet may improve urticaria symptoms. The authors therefore suggest screening for autoimmune diseases in pediatric patients with CSU, even in the absence of overt symptoms.	Retrospective study design. Small sample size. Lack of a control group. Non-standardized follow-up duration. Limited autoimmune testing. Family history of autoimmune disease was not analyzed. Self-reported UAS7 assessment. Limited testing for functional autoimmunity, restricted to ASST.	
Song et al. 2021 [48]	To evaluate the clinical effectiveness of omalizumab in children and adolescents with refractory chronic spontaneous urticaria (CSU) using the Urticaria Control Test; to assess quality-of-life improvement using the Children’s Dermatology Life Quality Index (CDLQI); and to evaluate safety and tolerability.	Pediatric patients aged 3–16 years with antihistamine-refractory chronic urticaria, predominantly CSU, treated with omalizumab for 16 weeks.	12	Persistence of CSU at follow-up.	Median disease duration was 23 months (IQR, 7–48), and median UAS7 was 28 (IQR, 21–42). Angioedema was present in 48.2% of patients, positive autoantibodies in 27.1%, positive ASST in 34.1%, and atopy, defined by positive SPT, in 27.9%. Remission rates were 10.6% at 1 year, 29.3% at 3 years, and 44.5% at 5 years. UAS7 > 28 was identified as a prognostic factor for disease persistence (OR, 6.22; 95% CI, 1.54–25.15; *p* = 0.010).	Remission of pediatric CSU is slow, with only 44.5% of patients achieving remission at 5 years. The only clinical factor significantly associated with disease persistence was a baseline UAS7 score > 28. Other clinical factors, including positive ASST, angioedema, and atopy, were not significantly associated with disease duration.	Retrospective study design. Single-center cohort. Lack of standardized regular follow-up assessments. Limited detailed data on pharmacological treatments. Relatively small sample size for multivariable analysis.	
Kasap et al. 2024 [49]	To evaluate the effect of omalizumab on quality of life and disease activity in patients with antihistamine-refractory chronic spontaneous urticaria (CSU), using the Urticaria Activity Score (UAS), Urticaria Control Test (UCT), and Chronic Urticaria Quality of Life Questionnaire (CU-Q2oL).	Patients with antihistamine-refractory CSU (median age, 18 years; IQR, 17–18), including both adolescents and adults.	15 pediatric patients (of 50 total participants).	Clinical effectiveness of omalizumab, assessed using the Urticaria Control Test (UCT) and the Children’s Dermatology Life Quality Index (CDLQI).	UCT scores improved significantly from a median of 2.5 (IQR, 0.0–5.8) at baseline to 12.0 (IQR, 1.3–13.8) after 4 weeks (*p* = 0.002) and 15.0 (IQR, 13.5–16.0) after 16 weeks (*p* = 0.002). After the first omalizumab dose, 67% of pediatric patients (8/12) achieved disease control (UCT ≥ 12). CDLQI scores improved from a median of 17.5 (IQR, 14.5–20.5) at baseline to 9.0 (IQR, 3.0–13.8) after 4 weeks (*p* = 0.003) and 2.0 (IQR, 0.0–6.8) after 16 weeks (*p* = 0.002). No adverse events were reported.	Omalizumab produced rapid and significant improvements in disease control and quality of life in pediatric patients with antihistamine-refractory CSU, with no reported adverse events during the study period.	Small sample size. Retrospective study design. Short follow-up duration. Limited laboratory assessment. The Urticaria Control Test (UCT) has not been validated for use in children.	
Galletta et al. 2025 [50]	To evaluate the clinical efficacy of omalizumab in children with antihistamine-refractory chronic spontaneous urticaria (CSU) and dupilumab in children with moderate-to-severe atopic dermatitis (AD) resistant to topical therapies. Efficacy was assessed using UAS7 for CSU and EASI for AD, together with improvements in pruritus NRS, sleep NRS, and quality of life (CDLQI). Safety, tolerability, adverse events, and treatment adherence over 12 months were also evaluated.	Pediatric patients aged 6–18 years (median age, 12 years) with antihistamine-refractory CSU or moderate-to-severe atopic dermatitis resistant to topical therapy.	42 children	Clinical efficacy and quality-of-life improvement associated with omalizumab treatment.	UAS decreased from a median of 35 (IQR, 28–35) at baseline to 7 (IQR, 0–7) after 3 months (*p* < 0.001). UCT increased from a median of 2 (IQR, 1.25–3) at baseline to 16 (IQR, 13–16) after 3 months (*p* < 0.001). CU-Q2oL decreased from a median of 70.5 (IQR, 66–74) at baseline to 23 (IQR, 23–28) after 3 months (*p* < 0.001).	Omalizumab was effective in improving quality of life and reducing CSU symptoms in antihistamine-refractory patients, including both adolescents and adults.	Limited sample size. Short follow-up duration. Observational study design, with no randomization or control group.	
Barzilai et al. 2023 [58]	To describe the epidemiological, clinical, and therapeutic characteristics of adult and pediatric patients with chronic spontaneous urticaria (CSU).	Patients (mean age, 12.9 ± 5.03 years) with clinically confirmed severe CSU (urticarial lesions persisting for >6 weeks) and no identifiable triggering factors.	23	Clinical effectiveness, assessed using disease-specific outcome measures, and the safety and tolerability of treatment.	Among patients treated with omalizumab for CSU, the UAS7 score decreased by 71.9% from baseline after 16 weeks of treatment. At 52 weeks, the reduction was maintained, reaching 75.3% below baseline. Children’s Dermatology Life Quality Index (CDLQI) scores improved significantly, reflecting substantial improvements in patients’ quality of life in parallel with symptom reduction.	Omalizumab was effective and well tolerated in pediatric patients with CSU, leading to marked reductions in pruritus and wheal activity, together with significant improvements in quality of life. The favorable safety profile was associated with high treatment adherence and no serious adverse events.	Retrospective study design. Limited sample size. Single-center study. Lack of a control group. Limited duration of follow-up. Variability in outcome assessment.	
Le M et al. 2022 [59]	To investigate the association between autoimmune diseases and chronic spontaneous urticaria (CSU) in pediatric patients.	Pediatric patients (median age, 9.4 years; interquartile range [IQR], 4.85–13.65 years) with a clinical diagnosis of CSU who were evaluated for the presence of autoantibodies associated with autoimmune diseases.	170 adults and 23 pediatric patients	Epidemiological, clinical, laboratory, and therapeutic characterization of adult and pediatric patients with CSU, with particular focus on autoimmune comorbidities and their therapeutic implications.	The prevalence of autoimmune comorbidities was similar in adults (59/170, 34.7%) and children (8/23, 34.8%; *p* = 1.00). Among pediatric patients, autoimmune liver disease (8.7%; *p* = 0.005 vs. adults) and inflammatory bowel disease (13.0%; *p* = 0.007 vs. adults) were significantly more frequent, whereas hypothyroidism (4.3%; *p* = 0.811) and Hashimoto thyroiditis (4.3%; *p* = 0.971) occurred at rates comparable to adults. Non-autoimmune/atopic comorbidities were less common in children than in adults (8.7% vs. 48.8%; *p* = 0.001), whereas atopy was more prevalent among pediatric patients (30.4% vs. 20.0%). Elevated C-reactive protein (>5 mg/L) was observed in 47.6% of adults and 17.4% of pediatric patients (*p* = 0.575). Second-generation H1-antihistamines were prescribed in 90.7% of patients overall. Omalizumab was used in 24.7% of adults and 17.4% of children (*p* = 0.609). Intravenous corticosteroids were administered more frequently to adults than children (35.3% vs. 0%; *p* = 0.001), as were oral corticosteroids (70.0% vs. 39.1%; *p* = 0.007)	Among 193 patients with CSU (170 adults and 23 children), the overall prevalence of autoimmune comorbidities was similar in adults and children (approximately 35%), although the spectrum differed. Inflammatory bowel disease and autoimmune liver disease predominated in pediatric patients, whereas autoimmune thyroid disorders were more common in adults. Atopy was more prevalent in children. Adults required systemic therapies more frequently, whereas pediatric patients responded more favorably to second-generation antihistamines.	Small pediatric sample size (23 patients). Retrospective study design. Single-center study. Lack of standardized measures of CSU disease severity.	
Buono et al. 2024 [54]	To analyze the clinical characteristics, disease duration, underlying causes, comorbidities, and treatment options in children with chronic urticaria (CU).	Children aged 0–18 years with a diagnosis of chronic spontaneous urticaria (CSU), followed at the Pediatric Allergy and Clinical Immunology Center of the University Hospital of Parma.	191 patients	Prevalence of autoimmune diseases among pediatric patients with chronic spontaneous urticaria.	Autoimmune comorbidities included hypothyroidism in 4/191 patients (2.10%), lupus in 1/191 (0.52%), juvenile idiopathic arthritis in 2/191 (1.05%), and type 1 diabetes mellitus in 3/191 (1.57%). Atopic comorbidities included asthma in 26/191 patients (13.6%), allergic rhinitis in 12/191 (6.3%), and atopic dermatitis in 23/191 (12.0%).	The prevalence of hypothyroidism, lupus, juvenile idiopathic arthritis, and type 1 diabetes mellitus was higher in the study cohort than in the general pediatric population.	Limited sample size. Possible underestimation of disease prevalence. Single-center study	
Finlay et al. 2017 [51]	To characterize the etiology, clinical features, laboratory findings, and treatment patterns of pediatric patients with chronic urticaria (CU).	Children with chronic urticaria evaluated at a tertiary pediatric allergy center.	37 patients	Etiological, clinical, laboratory, and therapeutic characterization of pediatric chronic urticaria.	Atopic comorbidities included allergic rhinitis in 9 patients (24.3%), allergic conjunctivitis in 8 (21.6%), and asthma in 7 (18.9%). Autoimmune diseases included celiac disease, Graves disease, and type 1 diabetes mellitus, each diagnosed in 1 patient (2.7%). The most common diagnosis was chronic spontaneous urticaria (86.5%). Other diagnoses included positive autologous serum skin test (ASST) (8.7%), urticarial vasculitis (5.4%), chronic inducible urticaria (5.4%), and chronic urticaria associated with Dientamoeba fragilis infection (5.6%). Second-generation H1-antihistamines were prescribed in 78.4% of patients, with a partial response in 62.2%. Systemic corticosteroids were used in 35.1% of patients, resulting in complete remission in 61.5%. Omalizumab was administered to 16.2% of patients, with a good clinical response in 83.3%. Montelukast was prescribed in 5.4% of patients, with no clinical responders. Conclusions	Chronic spontaneous urticaria was the predominant subtype of pediatric chronic urticaria. In the absence of clinical suspicion, routine laboratory investigations to identify an underlying trigger are generally unnecessary. Autoimmune diseases, particularly celiac disease and Graves disease, appeared to be more frequent than in the general pediatric population. Second-generation H1-antihistamines remain the first-line treatment and may be complemented by a short course of systemic corticosteroids during exacerbations. Omalizumab demonstrated excellent effectiveness in patients who did not respond adequately to first-line therapy.	Limited sample size. Retrospective study design. Single-center study. Lack of a control group	
**Atopic Dermatitis**								
Grillo et al. 2006 [80]	To evaluate the impact of an intensive educational program for children with atopic eczema and their parents on disease severity, quality of life, and family burden.	Pediatric patients (0–16 years) with atopic eczema and their parents from the Adelaide metropolitan area.	61 patients randomized to the intervention (*n* = 32) or control (*n* = 29) group.	SCORAD (disease severity); Children’s Dermatology Life Quality Index (CDLQI); Infants’ Dermatitis Quality of Life Index (IDQOL); Dermatitis Family Impact (DFI) questionnaire.	A significant improvement in SCORAD was observed in the intervention group (−44.9% at week 4 and −53.9% at week 12). CDLQI scores improved significantly in the intervention group at week 12 (−78.4% vs. −26.9% in the control group; *p* = 0.004). No significant between-group differences were observed for IDQOL or DFI despite the improvement in SCORAD. Children aged 5–12 years demonstrated particularly strong engagement in self-management.	Educational interventions can substantially reduce eczema severity and improve quality of life in school-aged children, even without changes in medical treatment. However, the benefits for family burden and infant quality of life remain less clear, highlighting the need for more comprehensive and long-term educational strategies.	Small sample size and single-center design. Short study duration (12 weeks) with no long-term follow-up. No significant improvement in family impact (DFI) or infant quality of life (IDQOL) despite the reduction in SCORAD. Parent-reported outcomes may have introduced reporting bias in quality-of-life assessments.	1, 4
Staab et al. 2006 [79]	To evaluate the long-term effectiveness of structured, age-specific educational programs on disease control and quality of life in children and adolescents with moderate-to-severe atopic dermatitis.	Children and adolescents (3 months–18 years) with moderate-to-severe atopic dermatitis.	992 participants, stratified as follows: 3 months–7 years: *n* = 518 (intervention: 274; control: 244); 8–12 years: *n* = 185 (intervention: 102; control: 83); 13–18 years: *n* = 120 (intervention: 70; control: 50).	Eczema severity (SCORAD); subjective disease severity and itch-related behavior; parental quality of life (for children < 13 years).	Participants in all age groups assigned to the intervention showed significantly greater reductions in eczema severity scores than controls (e.g., −19.7 vs. −5.2 among adolescents; *p* < 0.0001). Significant improvements were also observed in subjective disease severity and itch-related behavior, particularly in the catastrophizing domain. Parental quality of life improved significantly across several domains, with the greatest benefit observed in the 3-month to 7-year age group, in which all five subscales improved (*p* < 0.0001).	Structured, age-tailored educational programs are effective in improving both disease severity and quality of life in pediatric patients with atopic dermatitis over a 12-month period. These interventions should be considered a valuable adjunct to conventional therapy and incorporated into routine clinical care.	Blinding was not feasible because of the nature of the intervention. The control group had a higher dropout rate than the intervention group (24% vs. 10%). The observed treatment effect may have been influenced by greater motivation among participants in the intervention group.	1
Shaw et al. 2008 [81]	To evaluate whether individualized education delivered by an “atopic dermatitis educator” improves disease severity and quality of life in children with atopic dermatitis compared with standard care.	Pediatric patients (0–18 years) with atopic dermatitis recruited from a tertiary pediatric dermatology clinic in North Carolina (USA).	151 participants were enrolled; 106 completed the study (intervention group: *n* = 51; control group: *n* = 55).	SCORAD; Infants’ Dermatitis Quality of Life Index (IDQOL); Children’s Dermatology Life Quality Index (CDLQI).	No statistically significant differences were observed between the intervention and control groups in improvements in SCORAD (31% vs. 21%, *p* = 0.27), IDQOL (31% vs. 27%, *p* = 0.77), or CDLQI (−3% vs. +15%, *p* = 0.51). Caregiver satisfaction was reported anecdotally but was not formally assessed. Both groups experienced moderate improvement over time, regardless of the additional educational intervention.	Individualized education provided by an atopic dermatitis educator did not result in significant improvements in disease severity or quality of life beyond those achieved with standard care, suggesting that comprehensive routine education may already provide substantial clinical benefit.	High dropout rate (30%). The study may have been underpowered because of participant attrition and baseline variability in quality-of-life scores. The control group had already received extensive education as part of standard care. Caregiver knowledge and satisfaction were not formally evaluated.	1, 4
Pustisek et al. 2016 [74]	To evaluate the effect of a structured short-term educational program for parents on: (i) atopic dermatitis severity in children (3 months–7 years); (ii) pruritus and sleep disturbance; (iii) parental stress and anxiety; and (iv) family quality of life.	Parents of children aged 3 months–7 years with moderate-to-severe atopic dermatitis.	134 participants were enrolled; 128 completed the study (intervention group: *n* = 64; control group: *n* = 64).	Changes in SCORAD and PO-SCORAD (eczema severity); pruritus and sleep disturbance; parental stress (Perceived Stress Scale, PSS); anxiety (State–Trait Anxiety Inventory, STAI); and family quality of life (Family Dermatology Life Quality Index, FDLQI).	The intervention group showed significantly lower SCORAD and PO-SCORAD scores (*p* < 0.001), reduced pruritus (*p* < 0.001), fewer sleep disturbances (*p* = 0.001), lower perceived stress (*p* = 0.024), reduced anxiety (*p* = 0.042), and improved family quality of life (overall FDLQI *p* = 0.006), particularly in the emotional and physical well-being domains and in the time devoted to caregiving.	A single 2 h structured educational session for parents significantly improved disease severity in children with atopic dermatitis, enhanced parental psychological well-being, and improved family quality of life.	Short follow-up period (2 months), preventing assessment of long-term effectiveness. Acquisition of disease-related knowledge and corticosteroid phobia were not evaluated after the intervention. Participant satisfaction with the educational program was not formally assessed. Baseline psychological measures (stress and anxiety) were higher in the intervention group, potentially introducing imbalance between groups.	1, 4
Barbarot et al. 2024 [92]	To evaluate the correlation between PO-SCORAD (Patient-Oriented SCORAD), SCORAD, the Patient-Oriented Eczema Measure (POEM), and the Investigator’s Global Assessment (IGA) in young children with atopic dermatitis (AD); to assess the interpretability of SCORAD and PO-SCORAD scores; and to determine the clinical utility of PO-SCORAD, particularly its ability to monitor disease severity between clinic visits and predict disease flares.	Children aged 2–6 years with mild-to-moderate atopic dermatitis.	335 participants.	Correlation coefficients between SCORAD, PO-SCORAD, POEM, and IGA; agreement between SCORAD score ranges and IGA severity categories; comparison of the area under the curve (AUC) over time for PO-SCORAD versus SCORAD; predictive value of PO-SCORAD scores for disease flares.	PO-SCORAD showed a strong correlation with SCORAD (r = 0.874) and POEM (r = 0.734). The best agreement between SCORAD and IGA was observed using the following SCORAD cut-offs: <12 (clear/almost clear), 12–25 (mild), and ≥25 (moderate/severe) (κ = 0.68). Over the 8-week follow-up, the AUC was significantly greater for PO-SCORAD than for SCORAD (*p* = 0.0002), indicating greater sensitivity to disease fluctuations. PO-SCORAD scores were significantly higher seven days before a disease flare than during stable periods (mean 13.85 vs. 7.13; *p* < 0.0001), with erythema representing the strongest individual predictor of impending exacerbation.	PO-SCORAD is a valid, reliable, and user-friendly instrument for assessing atopic dermatitis severity in children. Frequent use (e.g., twice weekly) may allow closer disease monitoring and earlier identification of disease flares than less frequent physician-based assessments. PO-SCORAD may therefore represent a valuable tool for therapeutic education and patient self-management in pediatric atopic dermatitis.	Post hoc analysis. Short follow-up period. Patients with severe atopic dermatitis and adults were not included, limiting the generalizability of the findings. The clinical interpretation of the AUC measurements was not fully established.	2
Flohr et al. 2023 [83]	To evaluate the effects of dupilumab plus topical corticosteroids (TCS) on patient-reported symptoms, assessed using POEM, and health-related quality of life, assessed using CDLQI, in children aged 6–11 years with severe atopic dermatitis.	Pediatric patients aged 6–11 years with severe atopic dermatitis.	367 randomized patients. Two treatment arms were analyzed in this post hoc study: dupilumab 300 mg every 4 weeks plus TCS (*n* = 122) and dupilumab 200 mg every 2 weeks for patients weighing ≥ 30 kg plus TCS (*n* = 59), with matched placebo groups.	Change in total POEM score; change in total CDLQI score; proportion of patients achieving the minimum clinically important difference (MCID, ≥6-point reduction); proportion achieving POEM/CDLQI scores of 0–1.	POEM scores decreased significantly from baseline by week 2, with improvements maintained through week 16 (*p* < 0.0001). At week 16, 81.7% (q4w) and 79.3% (q2w) of dupilumab-treated children achieved the MCID, compared with 31.1% and 32.0% in the matched placebo groups. CDLQI scores also improved significantly: 77.3% (q4w) and 80.8% (q2w) achieved the MCID, compared with 42.3% and 35.8% in the placebo groups (*p* < 0.0001). A significantly greater proportion of children achieved CDLQI scores of 0–1, indicating no effect on quality of life: 31.3% and 35.1% versus 9.5% and 10.2% in the placebo groups. Improvements were observed across all individual POEM and CDLQI items.	Dupilumab combined with topical corticosteroids produced rapid, significant, and clinically meaningful improvements in both patient-reported symptoms and quality of life in children aged 6–11 years with severe atopic dermatitis. Patient-reported outcomes, including POEM and CDLQI, support the added value of systemic therapy in this age group and highlight the importance of routine quality-of-life monitoring in pediatric atopic dermatitis.	Post hoc nature of the analysis. Missing data were handled as non-response, which may have underestimated some treatment benefits. The short study duration (16 weeks) limited assessment of long-term patient-reported outcomes, although longer-term reference data are available.	1
Boguniewicz et al. 2024 [87]	To evaluate whether the presence of type 2 inflammatory comorbidities (asthma, allergic rhinitis, and food allergy) influences the efficacy and safety of dupilumab in children aged 6 months to 5 years with moderate-to-severe atopic dermatitis.	Children aged 6 months to 5 years with moderate-to-severe atopic dermatitis.	162 participants: 83 received dupilumab plus topical corticosteroids (TCS), and 79 received placebo plus TCS.	Proportion of patients achieving an Investigator’s Global Assessment (IGA) score of 0/1 (clear or almost clear skin); proportion achieving ≥ 75% improvement in the Eczema Area and Severity Index (EASI-75); proportion achieving a ≥4-point reduction in the Worst Scratch/Itch Numeric Rating Scale (WSI-NRS); safety profile, including adverse events and infections.	Dupilumab demonstrated significantly greater efficacy than placebo across all efficacy endpoints, regardless of the presence of type 2 inflammatory comorbidities. Patients with or without asthma, allergic rhinitis, or food allergy were significantly more likely to achieve IGA 0/1 and EASI-75 when treated with dupilumab. A greater proportion of dupilumab-treated patients achieved a ≥4-point improvement in WSI-NRS, particularly among children without asthma (53% vs. 7%; *p* < 0.0001). Dupilumab was generally well tolerated, with adverse events occurring more frequently and being more severe in the placebo group, especially among patients with comorbidities.	Dupilumab improves the signs and symptoms of moderate-to-severe atopic dermatitis in young children regardless of coexisting type 2 inflammatory comorbidities. Its efficacy and safety are consistent with previous studies and suggest a potential role in modifying disease progression associated with the atopic march. These findings support the use of dupilumab in pediatric atopic dermatitis, including in children with concomitant allergic diseases.	The limited number of participants younger than 2 years restricts the generalizability of the findings to this age group. Comorbidities were identified based on caregiver reports, which may have introduced recall or misclassification bias. As this was a post hoc analysis, not all subgroup comparisons were powered or formally tested for statistical significance.	1
Paller et al. 2024 [88]	To evaluate the effect of dupilumab treatment on caregiver-reported skin pain in children aged 6 months to 5 years with moderate-to-severe atopic dermatitis, including those who did not meet standard clinical improvement endpoints (IGA 0/1 or EASI-75).	Children aged 6 months to 5 years with moderate-to-severe atopic dermatitis.	162 participants: 83 received dupilumab plus topical corticosteroids (TCS), and 79 received placebo plus TCS. Subgroup analyses were conducted in 136 patients with IGA > 1 at week 16 and in 110 patients who did not achieve EASI-75.	Change in caregiver-reported skin pain NRS score (0–10 scale) from baseline to week 16; proportion of patients achieving a ≥4-point improvement in skin pain NRS; subgroup analyses among patients who did not achieve standard clinical endpoints (IGA > 1 or no EASI-75 response).	At week 16, mean skin pain NRS decreased significantly more with dupilumab than with placebo (−3.93 vs. −0.62; *p* < 0.0001). A ≥4-point improvement in skin pain NRS was achieved by 47.2% of dupilumab-treated patients compared with 10.8% of placebo-treated patients (*p* < 0.0001). In the IGA > 1 and non-EASI-75 subgroups, dupilumab still produced significantly greater reductions in skin pain, despite patients not meeting standard clinical response endpoints. Significant improvement was observed as early as week 1 and was maintained through week 16.	Dupilumab plus low-potency TCS provides rapid and clinically meaningful reductions in skin pain in young children with moderate-to-severe atopic dermatitis, including those who do not achieve traditional clinical response endpoints. These findings highlight the importance of incorporating patient- or caregiver-reported outcomes, such as skin pain, into therapeutic efficacy assessments in pediatric atopic dermatitis.	Post hoc nature of the analysis, with nominal *p* values. Short study duration (16 weeks). Limited number of very young children (<2 years). Caregivers may have had difficulty reliably distinguishing skin pain from pruritus in young children.	1
Paller et al. 2025 [86]	To evaluate the effects of dupilumab on caregiver-reported symptoms and health-related quality of life (QoL) in young children with moderate-to-severe atopic dermatitis.	Children aged 6 months to 5 years with moderate-to-severe atopic dermatitis.	162 participants: 83 received dupilumab plus topical corticosteroids (TCS), and 79 received placebo plus TCS.	Change from baseline to week 16 in caregiver-reported symptoms (e.g., pruritus and sleep quality); improvements in patient and caregiver quality of life.	Dupilumab resulted in significant improvements in caregiver-reported symptoms and health-related quality of life compared with placebo. Improvements were evident as early as week 4 and were maintained through week 16. Caregiver and family quality of life also improved rapidly and significantly in the dupilumab group.	Dupilumab combined with topical corticosteroids significantly improved caregiver-reported symptoms and both patient and family quality of life in young children with moderate-to-severe atopic dermatitis. These findings underscore the importance of incorporating caregiver-reported outcomes and quality-of-life measures into the assessment of treatment response in pediatric atopic dermatitis.	Few participants were younger than 2 years, limiting the generalizability of the findings to this age group. Statistical significance was reported only for prespecified endpoints. The Infant’s Dermatitis Quality of Life Index (IDQoL) was adapted from the Children’s Dermatology Life Quality Index (CDLQI), which may affect comparability across studies.	1
Minasyan et al. 2015 [89]	To validate the Comprehensive Early Childhood Allergy Questionnaire (CECAQ) as a parent-reported instrument for identifying atopic dermatitis, asthma, and IgE-mediated food allergy in children aged 1–5 years.	Children aged 1–5 years with at least one allergic disease.	150 participants: 77 with a diagnosis of at least one allergic disease and 73 healthy controls.	Sensitivity, specificity, and reproducibility of the CECAQ compared with the clinical diagnosis of: (i) atopic dermatitis (AD); (ii) asthma; and (iii) IgE-mediated food allergy (FA).	The questionnaire demonstrated an overall sensitivity of 0.93 and specificity of 0.79. Disease-specific sensitivity/specificity values were 0.94/0.88 for food allergy, 0.88/0.83 for atopic dermatitis, and 0.85/0.91 for asthma. Reproducibility was excellent, with Cohen’s κ values of up to 0.90.	The CECAQ is the first validated questionnaire designed to simultaneously identify atopic dermatitis, asthma, and IgE-mediated food allergy in children aged 1–5 years. It demonstrated good validity and reproducibility and represents a valuable tool for clinical and epidemiological studies of pediatric allergic diseases. Further validation in different populations and languages is warranted to confirm its broader applicability.	Clinical diagnoses were established by three different pediatricians, introducing potential interobserver variability. Oral food challenges were not performed to confirm food allergy diagnoses. Validation was conducted at a single center and only in the English language.	2
Tran et al. 2018 [67]	To investigate whether allergic sensitization modifies the risk of developing allergic diseases, including asthma, allergic rhinitis, food allergy, and persistent atopic dermatitis, in children with atopic dermatitis during infancy.	Infants aged 0–3 years enrolled in the CHILD study.	2311 participants	Incidence at 3 years of age of asthma, allergic rhinitis, food allergy, and atopic dermatitis. Main exposure variables at 1 year of age were atopic dermatitis, defined according to the UK Working Party criteria, and allergic sensitization, defined as a positive skin prick test with a wheal ≥ 2 mm.	Atopic dermatitis without sensitization was not associated with an increased risk of asthma (adjusted risk ratio [aRR], 0.46; 95% CI, 0.11–1.93). In contrast, atopic dermatitis with sensitization was associated with a more than sevenfold increased risk of asthma (aRR, 7.04; 95% CI, 4.13–11.99), with significant additive and multiplicative interaction between dermatitis and sensitization. For food allergy, atopic dermatitis combined with sensitization was associated with a 33-fold increased risk (aRR, 33.79), with a positive additive interaction (RERI, 15.11; 95% CI, 4.19–35.36). For allergic rhinitis, atopic dermatitis with sensitization was associated with an 11-fold increased risk (aRR, 11.75), although no significant interaction was detected. The risk of atopic dermatitis at 3 years was increased both in children with early dermatitis (aRR, 3.79) and in sensitized children (aRR, 2.43), with no significant interaction between dermatitis and sensitization.	Isolated atopic dermatitis during infancy does not increase the risk of asthma unless accompanied by allergic sensitization. The combination of atopic dermatitis and allergic sensitization identifies a high-risk group for asthma and food allergy. Screening for both conditions in early childhood may help target preventive strategies more effectively.	Filaggrin (FLG) mutations, a key marker of impaired skin barrier function in atopic dermatitis, were not genotyped. Asthma diagnosis at 3 years of age may be unreliable because no gold-standard test was used. Food allergy was not confirmed by oral food challenge. Mild cases of atopic dermatitis may have been underdiagnosed. Findings may not be generalizable to populations with different genetic backgrounds, environmental exposures, or healthcare systems.	1
Hon et al. 2019 [94]	To evaluate the usefulness of the Pediatric Allergic Disease Quality of Life Questionnaire (PADQLQ) for assessing quality of life in children with atopic dermatitis (AD) and to determine its correlation with established AD severity and quality-of-life measures, including the Patient-Oriented Eczema Measure (POEM), Nottingham Eczema Severity Score (NESS), and Children’s Dermatology Life Quality Index (CDLQI).	Children aged 6–16 years with physician-diagnosed atopic dermatitis.	132 participants	Correlations between PADQLQ and AD-specific instruments (POEM, NESS, and CDLQI); ability of PADQLQ to capture symptoms of coexisting allergic conditions, including asthma, allergic rhinitis, and allergic conjunctivitis; agreement between PADQLQ and CDLQI using Bland–Altman analysis; comparison of PADQLQ scores according to AD severity.	PADQLQ was significantly correlated with POEM (rho = 0.48) and NESS (rho = 0.51). CDLQI showed slightly stronger correlations with disease severity (POEM rho = 0.70; NESS rho = 0.68). Patients with severe AD had significantly higher PADQLQ scores across all domains, including practical, emotional, and symptom domains. Bland–Altman analysis showed good agreement between PADQLQ and CDLQI. PADQLQ also identified asthma symptoms in 20–30% of children, allergic conjunctivitis symptoms in 45–71%, and allergic rhinitis symptoms in 58–67%. Although eczema severity was not strongly correlated with coexisting allergic diseases, PADQLQ was able to detect their presence.	PADQLQ is a useful holistic quality-of-life instrument for children with atopic dermatitis, particularly those with concomitant allergic diseases reflecting the atopic march. It correlates with established AD severity and quality-of-life measures and adds value by capturing multiorgan allergic symptoms. It may be useful as a composite instrument in clinical and research settings to assess the overall disease burden of pediatric AD.	Cross-sectional design, with no longitudinal follow-up to assess responsiveness to change. Single-center population of Chinese ethnicity, limiting generalizability. PADQLQ was not originally validated in eczema-specific cohorts. It should not replace AD-specific instruments such as CDLQI, but may complement them, particularly in patients with multiple allergic comorbidities.	1, 2, 4
Andrade et al. 2023 [82]	To evaluate the effectiveness of a Spanish-language educational intervention delivered via WhatsApp in improving atopic dermatitis (AD) health literacy among Hispanic patients and healthcare providers.	Adults with atopic dermatitis and/or parents of children with atopic dermatitis, enrolled in three WhatsApp groups.	55 participants	Change in AD health literacy, assessed using pre-intervention, post-intervention, and 1-month follow-up surveys; knowledge acquisition; qualitative themes reported by participants (e.g., stigma and mental health).	AD health literacy improved by 14% immediately after the intervention (*p* < 0.001), with no significant decline one month later (*p* = 0.29). Participants with lower educational attainment demonstrated greater knowledge gains. Qualitative analyses revealed limited awareness of non-pharmacological treatments (e.g., wet-wrap therapy and bleach baths), as well as emotional distress and disease-related stigma. WhatsApp was well accepted and proved to be an effective platform for delivering educational content.	Culturally tailored digital educational interventions delivered through WhatsApp can effectively improve and sustain health literacy among underserved Spanish-speaking Hispanic individuals with atopic dermatitis. Future studies should assess long-term behavioral changes and explore the applicability of this approach to other dermatological conditions.	No control group or randomization. Small sample size with a predominantly female population. Short follow-up period (1 month). Potential selection bias due to recruitment through social media. Limited generalizability beyond Spanish-speaking Hispanic populations or users of the WhatsApp platform.	1
Liming et al. 2025 [85]	To evaluate the effectiveness and safety of dupilumab in pediatric patients with moderate-to-severe atopic dermatitis in routine clinical practice.	Pediatric patients aged 6–17 years with moderate-to-severe atopic dermatitis treated with dupilumab for at least 16 weeks at a single pediatric dermatology center in China between June 2021 and December 2022.	66 participants	EASI-50, EASI-75, and EASI-90 response rates at weeks 4, 8, 12, and 16; changes in pruritus NRS and sleep NRS scores; safety and adverse events.	At week 16, EASI-50, EASI-75, and EASI-90 were achieved by 98.48%, 89.39%, and 56.06% of patients, respectively. Pruritus NRS decreased from 7.17 ± 1.56 to 2.29 ± 1.26 (*p* < 0.001), and sleep NRS decreased from 6.41 ± 2.04 to 1.55 ± 1.12 (*p* < 0.001). Clinical improvement was observed as early as week 4 and continued through week 16. The most common adverse event was conjunctivitis (13.64%), followed by mild injection-site reactions (4.55%). No serious systemic adverse events were reported.	This real-world study confirms the rapid and sustained effectiveness of dupilumab in improving disease severity and pruritus in children with moderate-to-severe atopic dermatitis, with a favorable short-term safety profile. Despite its limitations, the findings support dupilumab as a valuable systemic treatment option for pediatric atopic dermatitis when conventional therapies are insufficient or not tolerated.	Retrospective, single-center design. Lack of a control group and short follow-up duration (16 weeks). Quality-of-life outcomes and long-term maintenance of response were not assessed.	1
Ren et al. 2025 [84]	To evaluate the clinical effectiveness and quality-of-life impact of dupilumab in children with atopic dermatitis, as well as its effects on inflammatory biomarkers, including IgE, eosinophils, IL-4, IL-13, and TARC.	Pediatric patients aged 2–15 years with moderate-to-severe atopic dermatitis.	54 participants.	Clinical scores: EASI, SCORAD, IGA, and pruritus NRS. Patient-reported outcomes: POEM and DLQI. Laboratory biomarkers: total IgE, eosinophil count, IL-4, IL-13, and TARC.	EASI decreased from 25.4 ± 6.2 to 8.8 ± 4.5, SCORAD from 38.6 ± 10.3 to 15.1 ± 8.4, and pruritus NRS from 6.9 ± 2.2 to 1.1 ± 0.7. The number of patients with IGA ≥ 3 decreased from 47 to 3. POEM improved from 13.5 ± 4.2 to 4.8 ± 1.6, and DLQI from 12.7 ± 5.4 to 3.3 ± 1.8. Total IgE decreased from 1200 ± 500 to 800 ± 300 kU/L, eosinophil count from 0.8 ± 0.3 to 0.4 ± 0.2 × 10^9^/L, and both IL-4 and IL-13 levels decreased significantly. TARC decreased from 50 ± 20 to 30 ± 15 pg/mL. Mild conjunctivitis occurred in 5 patients, and no major adverse events were reported.	This study supports the clinical utility of dupilumab in children with moderate-to-severe atopic dermatitis, demonstrating improvements in disease severity and quality of life together with reductions in Th2-related inflammatory biomarkers. Dupilumab was well tolerated, with limited adverse effects. Larger controlled studies are needed to confirm its long-term safety and comparative effectiveness.	No control group or randomization. Small sample size and single-center design. Short study duration (16 weeks). No comparison with standard therapies. Subjective assessment tools may have introduced reporting bias.	1

**Table A3:** Summary of non-original studies included in the analyses.

Study	Meta-Analysis	Aims	Population	Outcome	Results	Main Findings	PICO
**IgE-Mediated and Non-IgE Mediated Food Allergy and Eosinophilic Gastrointestinal Disease**							
Kelleher et al. 2022 [112]	Yes	To assess whether skin care interventions in infants, such as emollient application, can prevent the development of eczema and food allergy.	Healthy infants younger than 12 months, without pre-existing eczema or food allergy at randomization.	Incidence of eczema and food allergy at 1–3 years of age. Secondary outcomes included eczema severity, time to eczema onset, parent-reported immediate reactions to food allergens, allergic sensitization, and adverse events.	Skin care interventions probably do not alter the risk of eczema at 1–3 years of age (risk ratio [RR], 1.03; 95% confidence interval [CI], 0.81–1.31; moderate-certainty evidence). The effect on food allergy remains uncertain because of limited data	Skin care interventions, such as emollient use during the first year of life in healthy infants, are probably not effective in preventing eczema and may increase the risk of skin infections. The effects of skin care interventions on food allergy risk remain uncertain. Further research is needed to determine whether different approaches to infant skin care may promote or prevent eczema and to assess their effects on food allergy using robust outcome measures.	1
Riggioni et al. 2024 [33]	Yes	To evaluate the diagnostic accuracy of tests for IgE-mediated food allergy.	Not applicable (systematic review of diagnostic accuracy studies).	Sensitivity and specificity of diagnostic tests for IgE-mediated food allergy. Comparison of the diagnostic performance of different testing modalities.	Skin prick testing (SPT) using fresh cow’s milk and raw egg demonstrated high sensitivity (90% and 94%, respectively) for the diagnosis of cow’s milk allergy and cooked egg allergy. Component-resolved diagnostics showed high specificity: Ara h 2-specific IgE (92%), Cor a 14-specific IgE (95%), Ana o 3-specific IgE (94%), casein-specific IgE (93%), and ovomucoid-specific IgE (92% for raw egg allergy and 91% for cooked egg allergy). The basophil activation test (BAT) demonstrated high specificity for peanut allergy (90%) and sesame allergy (93%).	Skin prick tests and extract-specific IgE assays provide high sensitivity, whereas component-specific IgE assays and the basophil activation test (BAT) offer high specificity, supporting the diagnosis of individual IgE-mediated food allergies.	2
Cuomo et al. 2017 [34]	No	To analyze positive predictive values for allergen-specific IgE and skin prick testing in the diagnosis of fresh and baked cow’s milk allergy according to age.	Children with suspected cow’s milk allergy.	Assessment of potential sIgE and/or SPT cut-off values for the diagnosis of cow’s milk allergy in pediatric patients.	In children younger than 2 years, cow’s milk allergy was highly likely when cow’s milk-specific IgE levels were ≥5 kUA/L, commercial extract SPT wheal size was >6 mm, or prick-by-prick testing with fresh milk showed a wheal size > 8 mm. In children older than 2 years, clear cut-off values for diagnosing allergy to fresh or baked cow’s milk were difficult to establish because of substantial variability across thresholds.	None of the cut-off values proposed in the literature can reliably confirm the diagnosis of cow’s milk allergy, either to fresh pasteurized milk or baked milk. However, in children younger than 2 years, sIgE and SPT cut-off values appear more consistent and may be proposed for clinical use.	2
Calvani et al. 2021 [37]	No	To provide an updated overview of the clinical presentation and both traditional and emerging diagnostic tools for non-IgE-mediated and mixed IgE/non-IgE-mediated gastrointestinal food allergies in infants and young children.	Infants and young children in the first years of life with suspected non-IgE-mediated or mixed IgE/non-IgE-mediated gastrointestinal food allergy.	Description of clinical features and diagnostic accuracy of both traditional and emerging diagnostic tools.	Non-IgE-mediated gastrointestinal food allergies, including FPIAP, FPIES, FPE, and EGIDs, have specific and well-characterized clinical presentations	In many cases, such as FPIAP, FPIES, and FPE, an oral food challenge (OFC) may be attempted, whereas in other conditions, such as EGIDs, endoscopy with biopsy of the affected gastrointestinal tract is essential for diagnosis, particularly in eosinophilic esophagitis (EoE).	3
Cuomo et al. 2023 [43]	No	To determine the sensitivity, specificity, positive predictive value (PPV), and negative predictive value (NPV) of the atopy patch test (APT).	Infants and young children in the first years of life with suspected non-IgE-mediated or mixed IgE/non-IgE-mediated gastrointestinal food allergy.	Diagnostic utility of the atopy patch test (APT) for non-IgE-mediated food allergies	The atopy patch test (APT) demonstrated high specificity (94%; 95% CI, 0.88–0.97) in patients with food protein-induced motility disorders (FPIMDs). Among patients with cow’s milk allergy, APT showed an even higher pooled specificity (96%; 95% CI, 0.88–0.97) and the highest overall diagnostic accuracy (area under the receiver operating characteristic curve [AUC], 0.93).	The atopy patch test (APT) appears to be useful in identifying the causative foods in children with food protein-induced gastrointestinal motility disorders.	4
**Urticaria and Angioedema**							
Williams 2020 [45]	No	To examine the causes of chronic urticaria in children, assess its impact on quality of life, disease duration, healthcare costs, and comorbidities; review international and US guidelines; compare guideline recommendations with real-world clinical practice; and propose strategies to improve adherence to guideline-based care in daily practice.	Children younger than 18 years with chronic urticaria.	To highlight the discrepancy between guideline recommendations for the management of pediatric chronic urticaria and real-world clinical practice, emphasizing clinical implications and strategies to improve guideline adherence.	The prevalence of chronic urticaria is estimated at 0.1–0.3% in children and approximately 3% in adults. Chronic inducible urticaria accounts for 15–20% of cases, while autoimmune urticaria accounts for 30–40%. Chronic urticaria has a substantial impact on quality of life: 7.4% of children missed at least one week of school because of urticaria. Oral corticosteroids were used in 17–35% of children, whereas non-sedating antihistamines were prescribed to only 11.5% of patients in one study. Anxiolytics were prescribed in 10–19% of children, despite not being indicated. Remission rates ranged from 10% to 32% at 1 year, 31% to 54% at 3 years, and 38% to 72% at 5 years. In a study of 98 Asian children, disease control was achieved with standard-dose antihistamines in 50% of patients, double-dose antihistamines in 35.7%, triple-dose antihistamines in 6.1%, and quadruple-dose antihistamines in 5.3%.	The study highlights a gap between guideline recommendations and clinical practice. Although guidelines recommend a stepwise approach based on non-sedating H1-antihistamines, real-world practice shows excessive use of oral corticosteroids, sedating medications, anxiolytics, and ineffective topical therapies. Physicians often switch treatments rather than escalating them sequentially. Inadequate management may worsen the overall disease burden. Primary care physicians play a key role in pediatric chronic urticaria management but often lack sufficient support. Greater adherence to guideline-based care is needed.	1, 2, 3
Caffarelli et al. 2019 [56]	No	To develop clinical guidelines for the diagnosis and management of chronic urticaria in children; provide evidence-based recommendations on disease causes, diagnosis, treatment, and natural history; identify triggering factors; improve clinical practice through decision algorithms and personalized therapeutic strategies; and address the lack of pediatric-specific guidelines.	Children younger than 18 years with chronic urticaria.	Development of structured clinical guidelines, based on a systematic review of the literature, to improve the diagnostic and therapeutic management of pediatric chronic urticaria.	The prevalence of chronic urticaria in children is <1%, with an annual incidence of 0.6–2.1 per 1000 children in Italy. Remission rates are 10–32% at 1 year, 31–54% at 3 years, and 38–72% at 5 years, with an annual remission incidence of 10.3%. Chronic inducible urticaria accounts for 6.2–52.9% of cases, and an identifiable physical trigger is reported in 22–40.1% of inducible cases. Parasitic infections have been reported in up to 37.8% of children with chronic urticaria, although no definite causal relationship has been established. Positive ASST occurs in 22–53.5% of children with chronic urticaria. Thyroid autoimmunity is 10–30 times more prevalent in children with CSU than in the general population, and celiac disease prevalence may be increased up to 8–10-fold.	Allergy testing is not recommended in the absence of clinical suspicion. Bacterial and parasitic infections are not documented causes of chronic urticaria in most cases. Autoimmune factors are present in approximately 30–50% of children with chronic urticaria. Second-generation H1-antihistamines are recommended as first-line treatment; if symptoms remain uncontrolled, the dose may be increased up to fourfold. Omalizumab is recommended for severe refractory cases.	2, 3
Poddighe et al. 2016 [60]	No	To collect and analyze all available evidence on the diagnosis and treatment of autoimmune chronic spontaneous urticaria (CSU) in children, including recent developments and patents.	Children with chronic spontaneous urticaria.	Diagnosis and treatment of autoimmune CSU in pediatric patients.	In children with CSU, a substantial proportion of cases may have an autoimmune basis, mediated by autoantibodies against the high-affinity IgE receptor or against IgE itself. However, the true prevalence is difficult to establish because diagnostic approaches are incomplete and rely mainly on ASST, with limited use of confirmatory in vitro tests. In children with CSU, idiopathic cases account for more than 50% of cases. In adults, autoimmune mechanisms are estimated to account for 30–40% of idiopathic cases; in children, reported rates vary, although ASST positivity is often observed in approximately 30–50% of studied patients. The prevalence of other autoimmune diseases in children with CSU is generally low, with only isolated cases of autoimmune thyroiditis or celiac disease reported in the cited studies.	The diagnosis of autoimmune CSU is mainly based on ASST, and many cases remain classified as idiopathic. A more comprehensive diagnostic approach capable of demonstrating an antibody-mediated mechanism would allow better patient stratification and more effective treatment. Identifying an autoimmune mechanism has therapeutic implications: in antihistamine-refractory cases, it may support the use of targeted anti-IgE therapy, such as omalizumab, while avoiding or delaying systemic immunosuppressive treatments.	3
Pines et al. 2021 [63]	No	To describe the pathophysiology and clinical features of angioedema, with particular emphasis on hereditary angioedema (HAE); distinguish histamine-mediated angioedema from bradykinin-mediated forms (including HAE, acquired angioedema, and angioedema associated with angiotensin-converting enzyme inhibitors [ACEIs]); provide practical guidance for the recognition, differential diagnosis, and management of HAE in the emergency department; and increase awareness among emergency physicians to reduce diagnostic delays and inappropriate treatment.	Patients presenting to the emergency department with angioedema, particularly those with hereditary angioedema (HAE) or angioedema not explained by allergic causes.	To improve the recognition and management of angioedema in the emergency setting by providing criteria to distinguish histamine-mediated from bradykinin-mediated forms, together with rapid diagnostic algorithms and targeted treatment recommendations aimed at reducing mortality, diagnostic delays, and inappropriate therapy.	The estimated prevalence of hereditary angioedema (HAE) is approximately 1 in 50,000 individuals. Approximately 30% of angioedema presentations to emergency departments in the United States are attributed to ACE inhibitor-associated angioedema. The incidence of angioedema among patients receiving ACE inhibitors is <1%. The risk of ACE inhibitor-induced angioedema is 3–4 times higher in individuals of African or Caribbean ancestry and approximately 50% higher in women than in men. Approximately 25% of HAE cases arise from de novo mutations without a family history. Laryngeal involvement occurs in up to 50% of patients with HAE during their lifetime, while abdominal attacks are reported in up to 93% of cases. Typical laboratory findings include low C4 levels; in type I HAE, both C1 inhibitor (C1-INH) concentration and function are reduced, whereas in type II HAE, C1-INH concentration is normal or elevated but functional activity is reduced. Serum tryptase levels remain normal, unlike in anaphylaxis. Histamine-mediated angioedema typically develops within minutes and resolves within minutes to hours, whereas bradykinin-mediated angioedema (HAE or ACE inhibitor-induced) develops over several hours and may persist for days.	Bradykinin-mediated angioedema differs fundamentally from histamine-mediated angioedema and does not respond to antihistamines, corticosteroids, or epinephrine. The absence of urticaria is an important clinical clue to a bradykinin-mediated mechanism. HAE is characterized by recurrent, non-pruritic episodes involving the gastrointestinal tract, upper airway, and extremities, with approximately one-quarter of cases occurring de novo. Prompt recognition is essential because laryngeal edema may progress rapidly and become life-threatening. Effective treatments include ecallantide, icatibant, C1-INH concentrates, and, when specific therapies are unavailable, fresh frozen plasma. Many patients with HAE carry on-demand medication for self-administration.	4, 5
Jacobs et al. 2021 [65]	No	To improve the early recognition of hereditary angioedema (HAE), which is frequently diagnosed late; highlight the differences between HAE and other forms of angioedema (e.g., histamine-mediated or ACE inhibitor-induced); provide an update on current therapeutic options for both acute treatment and long-term prophylaxis; and emphasize the importance of a multidisciplinary, individualized approach to HAE management.	Patients with hereditary angioedema (HAE), particularly type I and type II HAE due to C1 inhibitor (C1-INH) deficiency.	To increase clinical awareness of HAE due to C1-INH deficiency. To facilitate early and accurate diagnosis by distinguishing HAE from other forms of angioedema, including allergic and ACE inhibitor-induced angioedema. To optimize therapeutic management, including acute attack treatment, long-term prophylaxis, patient education, and self-management. To reduce diagnostic delays, inappropriate treatment, and life-threatening complications such as laryngeal edema.	The estimated prevalence of HAE is approximately 1 in 50,000 individuals. Approximately 25% of patients have de novo disease without a family history. The average diagnostic delay exceeds 10 years after symptom onset. Gastrointestinal attacks occur in approximately 90% of patients with HAE. More than 50% of patients experience at least one laryngeal attack during their lifetime. The disease typically begins during adolescence or early adulthood	HAE is characterized by recurrent, non-pruritic angioedema that typically occurs without urticaria and commonly affects the face, extremities, upper airway, and gastrointestinal tract. Abdominal pain is a frequent manifestation and is often misdiagnosed as an acute abdominal condition. Diagnosis is frequently delayed and should be based on measurement of serum C4 levels together with quantitative and functional C1 inhibitor (C1-INH) testing. Effective on-demand therapies include icatibant, ecallantide, and plasma-derived or recombinant C1-INH concentrates, while long-term prophylaxis may include lanadelumab, C1-INH replacement therapy, or attenuated androgens. Early recognition is essential because laryngeal edema may be fatal. Patients should receive an individualized management plan and have immediate access to on-demand medication.	5
Liotti et al. 2023 [62]	No	To provide clinical guidance for the diagnosis and management of angioedema without wheals (AEwW) in children, with particular emphasis on distinguishing histamine-, bradykinin-, and leukotriene-mediated forms; identifying hereditary angioedema (HAE) and its variants; managing idiopathic pediatric AE; and proposing a diagnostic algorithm and targeted therapeutic strategies.	Children and adolescents with isolated angioedema without wheals (AEwW), including acquired, hereditary, and idiopathic forms. The reported age range in the included studies extended from infancy (<1 year) to approximately 16 years.	Development of a diagnostic algorithm for pediatric AEwW based on the absence of wheals, response to medications (antihistamines, corticosteroids, etc.), identifiable triggers, family history, and clinical characteristics. Promotion of early recognition of hereditary angioedema (HAE), particularly in children with recurrent attacks, a positive family history, or lack of response to antihistamines. Classification of idiopathic AEwW into histaminergic and non-histaminergic subtypes. Recommendations for appropriate diagnostic investigations, including serum C4, C1 inhibitor (C1-INH) functional testing, and genetic testing.	The estimated prevalence of pediatric AEwW is approximately 1.6%. The mean age at symptom onset ranges from 7.0 to 7.8 years. The mean duration of histaminergic AEwW episodes is approximately 24 h. Up to 87% of patients with idiopathic histaminergic AEwW respond to antihistamine therapy. Among patients with idiopathic non-histaminergic AEwW, tranexamic acid was associated with clinical improvement in approximately 73% of cases, while omalizumab showed a response rate of approximately 63%.	AEwW in children can be classified into histamine-mediated, bradykinin-mediated (including HAE and ACE inhibitor-induced angioedema), and leukotriene-mediated forms (e.g., NSAID-associated angioedema). Histaminergic idiopathic AEwW typically responds to antihistamines, whereas non-histaminergic idiopathic AEwW may respond to omalizumab or tranexamic acid. Clinical features that support a diagnosis of bradykinin-mediated angioedema include slow symptom onset, duration exceeding 72 h, absence of pruritus, positive family history, and involvement of the gastrointestinal tract or upper airway. In contrast, histaminergic angioedema is usually pruritic, responds to antihistamines, and resolves within 48 h. The proposed diagnostic algorithm provides a practical framework for the diagnosis and follow-up of pediatric AEwW.	4
Di Agosta et al. 2021 [52]	No	To review and summarize current evidence on the impact of allergic and immunologic skin diseases on quality of life (QoL); describe the main QoL assessment tools, including generic, organ-specific, and disease-specific instruments; evaluate QoL impairment in patients with atopic dermatitis, allergic contact dermatitis, hereditary angioedema, cutaneous mastocytosis, and urticaria; assess the impact of therapies—particularly biologic agents—on QoL; and provide clinical recommendations to support multidisciplinary management and the routine integration of QoL assessment into clinical practice.	Adults and children with allergic and immunologic skin diseases.	Overall qualitative assessment of quality of life in patients with allergic and immunologic skin diseases, in relation to disease severity and treatment effectiveness, particularly with novel biologic therapies.	n chronic urticaria, treatment with omalizumab 300 mg every 4 weeks resulted in a mean reduction of 13.9 points in the Dermatology Life Quality Index (DLQI) across six studies. The Chronic Urticaria Quality of Life Questionnaire (CU-Q2oL) score improved by a mean of 42.3 points across three studies. In a real-world study, DLQI decreased by 75% in patients treated with omalizumab, compared with a 41% reduction in those receiving cyclosporine.	Quality of life is substantially impaired across allergic and immunologic skin diseases because of persistent pruritus, sleep disturbance, psychological distress, altered body image, impaired social functioning, and reduced work productivity. Quality-of-life assessment instruments are valuable but remain underused in routine clinical practice. Biologic therapies, particularly omalizumab, produce substantial improvements in quality of life, especially in patients with severe or treatment-refractory disease. Importantly, quality-of-life impairment does not always correlate with clinical disease severity, supporting the routine incorporation of patient-reported outcome measures into multidisciplinary care.	1
Caffarelli et al. 2020 [55]	No	To provide an updated overview of urticaria in children, distinguishing acute from chronic forms; classify urticaria according to clinical features and triggering factors; review the pathophysiological mechanisms underlying urticaria–angioedema syndrome, particularly mast cell activation and the release of histamine and other mediators; describe the diagnostic work-up and appropriate use of available tests; examine effective treatment strategies, including antihistamines, systemic corticosteroids, omalizumab, and other drugs; highlight recent advances in pediatric urticaria management; and identify unresolved issues, particularly in differential diagnosis and treatment-resistant disease.	Children with urticaria.	Synthesis of current evidence on pediatric urticaria, including classification, practical diagnostic and therapeutic guidance, and identification of areas where evidence or standardized approaches remain limited.	The overall prevalence of urticaria in children is estimated at 2–6%. The prevalence of chronic urticaria ranges from 0.1% to 0.3% in children. Chronic urticaria accounts for less than one-third of all pediatric urticaria cases. In a real-world Indian cohort of 296 children with chronic urticaria, 57.1% had spontaneous forms. A specific trigger was identified in only approximately 17–25% of cases. In the Indian study, 72–90% of children were controlled with non-sedating antihistamines alone; a sedating antihistamine was added in 20.6% for nocturnal pruritus, and only approximately 3–4% required short-course corticosteroids.	Acute urticaria in children is most commonly triggered by viral infections, which may account for up to 80% of cases, followed by food allergy, medications, vaccines, and physical stimuli such as cold or pressure. Chronic forms are usually spontaneous, and an identifiable cause is found only in a minority of cases. Autoimmune mechanisms may be involved, with IgG autoantibodies against IgE or FcεRIα reported in approximately half of patients with chronic urticaria and ASST positivity ranging from 10% to 50%. Chronic urticaria can substantially impair quality of life in children, affecting sleep, school performance, and social relationships, with psychological effects such as anxiety and depression also reported. A detailed clinical history is essential, while laboratory and allergy testing should be reserved for cases with a specific clinical suspicion. Second-generation H1-antihistamines are the first-line treatment; in refractory cases, dose escalation, omalizumab, or cyclosporine may be considered in selected patients according to international guidelines.	2, 3
Giavina Bianchi et al. 2018 [64]	No	To update the Brazilian guidelines for the diagnosis and management of hereditary angioedema (HAE) based on scientific and clinical evidence published since the 2011 guidelines.	Patients with hereditary angioedema, including all subtypes with and without C1 inhibitor (C1-INH) deficiency, as well as family members who may undergo clinical or genetic screening.	Improvement of the early and accurate diagnosis of hereditary angioedema (HAE). Reduction in HAE-related morbidity and mortality. Increased awareness of HAE among healthcare professionals. Standardization of diagnostic and classification criteria for HAE in Brazil.	Patients consulted a mean of 4.4 physicians before receiving the correct diagnosis. Approximately 65% of patients were initially misdiagnosed. The estimated prevalence of HAE ranges from 1:10,000 to 1:150,000 individuals. Although laryngeal attacks account for <1% of all HAE attacks, more than 50% of patients experience at least one laryngeal episode during their lifetime. Without treatment, the estimated mortality ranges from 25% to 40%, primarily because of fatal laryngeal edema. Disease-related disability ranges from 20 to 100 days per year, depending on disease severity.	These updated Brazilian guidelines emphasize that hereditary angioedema remains substantially underdiagnosed and may be life-threatening if not recognized and treated promptly. All forms of HAE share excessive bradykinin production as the central pathogenic mechanism, and attacks may be precipitated by medications, hormonal factors, and other triggers. The guidelines recommend a structured diagnostic approach beginning with clinical suspicion and recognition of characteristic warning signs, followed by laboratory assessment of C1 inhibitor (C1-INH) levels and function together with complement testing, and genetic testing when appropriate. Accurate differentiation of HAE from other forms of angioedema and the adoption of standardized diagnostic criteria are considered essential to ensure timely diagnosis and appropriate management.	1, 2, 3
**Atopic Dermatitis**							
Kim et al. 2016 [66]	Yes	To determine the persistence rates of atopic dermatitis (AD) and identify clinical factors associated with prolonged disease persistence.	Children with persistent atopic dermatitis (72.7% of participants had persistent AD at one or more follow-up assessments); mean age at diagnosis: 1.6 ± 1.3 years. Mean follow-up: 3.9 ± 2.8 years, with approximately 21% of studies reporting follow-up ≥10 years.	(1) To quantify the average duration and persistence rates of pediatric AD; (2) to evaluate the association between AD persistence and clinical variables, including age at onset, prior disease duration, method of assessment (self-reported vs. physician-assessed), sex, allergen sensitization, and baseline disease severity.	The mean duration of AD persistence was 6.1 ± 0.02 years. Approximately 80% of cases resolved within 8 years of diagnosis, whereas fewer than 5% persisted beyond 20 years. Factors associated with longer disease persistence included: previous long-standing disease (>5 or >10 years); later age at onset (onset at 2–5 years: HR 2.65; 6–11 years: HR 4.22; 12–17 years: HR 2.04, compared with onset < 2 years); self-reported disease assessment (mean duration 9.6 years vs. 5.8 years for physician-assessed disease); female sex (mean duration 12.7 vs. 11.7 years in males; HR 1.15, *p* = 0.006); and greater baseline disease severity. Allergen sensitization was not significantly associated with disease persistence (*p* = 0.90).	The findings help identify children at increased risk of persistent atopic dermatitis who may benefit from closer long-term follow-up and specialist allergy care. Later disease onset, greater baseline severity, female sex, and a history of prolonged disease were associated with a higher likelihood of persistent AD, whereas allergen sensitization was not. Considerable heterogeneity among the included studies with respect to study design, follow-up duration, and methods used to assess disease persistence. Differences between self-reported and physician-assessed outcomes may have influenced persistence estimates. Residual confounding cannot be excluded because of the observational nature of the included studies.	1
Hill et al. 2016 [91]	Yes	To evaluate trends in the use of disease severity and quality-of-life (QoL) assessment instruments in randomized controlled trials (RCTs) of atopic dermatitis over a 5-year period.	Randomized controlled trials evaluating patients with atopic dermatitis.	Proportion of studies using disease severity and quality-of-life (QoL) assessment instruments; identification and frequency of specific severity and QoL measures; temporal trends in the use of these instruments.	Disease severity was assessed in all 135 RCTs (100%), whereas only 33% included quality-of-life outcomes. The most frequently used severity instruments were SCORAD (*n* = 79), pruritus visual analogue scale (VAS) (*n* = 30), Investigator’s Global Assessment (IGA) (*n* = 29), and Eczema Area and Severity Index (EASI) (*n* = 28). The most commonly used QoL instruments were the Dermatology Life Quality Index (DLQI; *n* = 20), Infants’ Dermatitis Quality of Life Index (IDQOL; *n* = 8), Children’s Dermatology Life Quality Index (CDLQI; *n* = 6), and Dermatitis Family Impact (DFI) questionnaire (*n* = 5). Overall, 62 different disease severity scales and 28 QoL instruments were identified. Approximately 75% of QoL instruments were used in only a single study, indicating substantial heterogeneity. Compared with a previous review, the proportion of RCTs assessing QoL increased from 18% to 33%, although QoL outcomes remained underrepresented.	Although the assessment of quality of life in atopic dermatitis RCTs has increased over time, QoL outcomes remain considerably underutilized compared with clinical severity measures. The large number of different assessment instruments highlights the need for greater standardization of outcome measures in future clinical trials. The review included only randomized controlled trials published within a predefined 5-year period, which may not reflect current practice. Considerable heterogeneity in the outcome measures used limited comparisons across studies. The review was descriptive and did not assess the psychometric performance or comparative validity of the identified instruments.	2, 4
Li et al. 2020 [77]	Yes	To evaluate the effectiveness of health education interventions on clinical severity and quality of life (QoL) in children with atopic dermatitis (AD) and their families.	Pooled analysis of 8 randomized controlled trials (RCTs) including children aged 0–18 years with atopic dermatitis. The studies were conducted in several countries, including the United Kingdom, Japan, Australia, Germany, China, and the United States.	SCORAD and objective SCORAD as eczema severity indices; Infants’ Dermatitis Quality of Life Index (IDQOL); Children’s Dermatology Life Quality Index (CDLQI); and Dermatitis Family Impact (DFI) questionnaire.	Health education significantly reduced SCORAD scores at multiple time points: 3 months (weighted mean difference [WMD], −7.57; *p* = 0.002), 6 months (WMD, −6.88; *p* = 0.001), and 12 months (WMD, −8.67; *p* = 0.0007). Objective SCORAD scores were also significantly lower at 6 and 12 months. IDQOL scores improved in the health education group at 3 months (WMD, −0.96; *p* = 0.04) and 6 months (WMD, −1.50; *p* = 0.01). No significant between-group differences were observed for CDLQI or DFI.	Health education is effective in reducing disease severity and improving quality of life in children with atopic dermatitis, particularly younger children. However, its impact on family quality of life and self-reported quality of life in older children appears less consistent. Standardized, age-specific, multicenter studies are needed to define the optimal structure and delivery of educational interventions.	1, 4
Gabes et al. 2023 [93]	Yes	To evaluate the effectiveness of health education interventions on clinical severity and quality of life (QoL) in children with atopic dermatitis (AD) and their families.	Pooled analysis of 8 randomized controlled trials (RCTs), including children aged 0–18 years with a diagnosis of atopic dermatitis. Studies were conducted in several countries, including the United Kingdom, Japan, Australia, Germany, China, and the United States.	SCORAD and objective SCORAD as measures of eczema severity; Infants’ Dermatitis Quality of Life Index (IDQOL); Children’s Dermatology Life Quality Index (CDLQI); and Dermatitis Family Impact (DFI) questionnaire.	Health education significantly reduced SCORAD scores at multiple time points: 3 months (weighted mean difference [WMD], −7.57; *p* = 0.002), 6 months (WMD, −6.88; *p* = 0.001), and 12 months (WMD, −8.67; *p* = 0.0007). Objective SCORAD scores were also significantly lower at 6 and 12 months. IDQOL scores improved in the health education group at 3 months (WMD, −0.96; *p* = 0.04) and 6 months (WMD, −1.50; *p* = 0.01). No significant between-group differences were observed for CDLQI or DFI.	Health education is effective in reducing disease severity and improving quality of life in children with atopic dermatitis, particularly in younger children. However, its impact on family quality of life and self-reported quality of life among older children appears less consistent. Standardized, age-specific, multicenter studies are needed to define the optimal structure and delivery of educational interventions.	2, 4
Oykhman et al. 2022 [97]	Yes	To determine the benefits and risks associated with elimination diets in the treatment of atopic dermatitis (AD).	Patients with atopic dermatitis undergoing elimination diets.	Effectiveness of elimination diets in improving AD severity and symptoms; assessment of potential risks, including nutritional deficiencies, loss of oral tolerance, and psychological burden.	Elimination diets may lead to a slight improvement in eczema severity: 50% of patients following an elimination diet versus 41% of those not following an elimination diet achieved a minimally clinically important improvement in SCORAD of 8.7 points (risk difference, 9%; 95% CI, 0–17). Modest effects were also observed for daytime pruritus (score range, 0–3; mean difference, −0.21; 95% CI, −0.57 to 0.15) and insomnia (score range, 0–3; mean difference, −0.47; 95% CI, −0.80 to −0.13). No credible subgroup differences were identified according to elimination strategy (empiric vs. test-guided) or food-specific sensitization.	Food allergen elimination provides inconsistent or modest improvements in atopic dermatitis severity. Potential risks include nutritional deficiencies, increased risk of developing IgE-mediated food allergy through loss of oral tolerance, and adverse effects on quality of life.	3
Yepes-Nunez et al. 2023 [96]	Yes	To evaluate the benefits and risks of allergen immunotherapy (AIT), including both subcutaneous immunotherapy (SCIT) and sublingual immunotherapy (SLIT), in patients with atopic dermatitis, with particular emphasis on clinical severity, quality of life, and safety outcomes.	Children and adults with atopic dermatitis, the majority of whom were sensitized to house dust mite (HDM).	Improvement in atopic dermatitis severity (e.g., ≥50% reduction in SCORAD); improvement in quality of life (e.g., ≥4-point improvement in DLQI); changes in pruritus, disease flares, and sleep disturbances; frequency and type of adverse events; treatment discontinuation due to adverse events.	AIT increased the proportion of patients achieving a ≥50% reduction in SCORAD (risk ratio [RR], 1.53; moderate-certainty evidence). Quality of life also improved significantly, with a greater proportion of patients achieving a clinically meaningful improvement in DLQI (RR, 1.44; moderate-certainty evidence). No statistically significant effects were observed on disease flares, pruritus, or sleep disturbances. Adverse events were more frequent with SCIT than with placebo (66% vs. 36%), whereas SLIT was associated with a lower incidence of adverse events (13% vs. 9%). Subgroup analyses suggested greater efficacy among patients sensitized to house dust mite (HDM).	Allergen immunotherapy may improve clinical severity and quality of life in selected patients with atopic dermatitis, particularly those sensitized to house dust mite. While SCIT appears to be associated with a higher frequency of adverse events than SLIT, both modalities demonstrate an acceptable safety profile when appropriately selected and monitored.	3
Andrade et al. 2024 [78]	Yes	To evaluate the effectiveness of educational interventions—particularly virtual versus face-to-face formats—on atopic dermatitis severity and quality-of-life outcomes across all age groups.	Pediatric and adult patients with atopic dermatitis, as well as caregivers of children with atopic dermatitis. A total of 18 studies were included.	Changes in atopic dermatitis severity assessed using validated instruments, including SCORAD (Scoring Atopic Dermatitis), POEM (Patient-Oriented Eczema Measure), and EASI (Eczema Area and Severity Index); quality of life assessed using DLQI and related instruments (e.g., CDLQI and IDQOL).	SCORAD (12 studies): significant reduction in disease severity (standardized mean difference [SMD], −0.65; 95% CI, −0.86 to −0.45; *p* < 0.00001), although with substantial heterogeneity (I^2^ = 81%). POEM (4 studies): significant improvement (SMD, −0.28; 95% CI, −0.49 to −0.07; *p* = 0.009; I^2^ = 47%). EASI (3 studies): significant improvement (mean difference, −0.80; 95% CI, −1.06 to −0.54; *p* < 0.00001; I^2^ = 0%). DLQI and related quality-of-life measures (7 studies): significant improvement (SMD, −0.36; 95% CI, −0.54 to −0.18; *p* = 0.0002; I^2^ = 54%). Both virtual and face-to-face educational interventions were effective, with no statistically significant differences in POEM outcomes between delivery formats. Benefits were observed in both pediatric and adult populations, although adult-specific evidence was limited.	Educational interventions for patients with atopic dermatitis and their caregivers significantly reduce disease severity and improve quality of life. Virtual educational programs appear to be as effective as face-to-face interventions and may represent a practical alternative. However, additional studies in adult populations and the development of more structured and interactive educational models are warranted.	1, 2, 4
Vassilopoulou et al. 2024 [98]	Yes	To evaluate the efficacy and safety of different nutritional and dietary strategies, excluding elimination diets for IgE-mediated food allergy, in improving clinical outcomes in children and adults with atopic dermatitis.	Children and adults with atopic dermatitis. Interventions included probiotics, prebiotics, synbiotics, vitamins, minerals, fatty acids, hydrolyzed formulas, and mixed dietary interventions.	Improvement in atopic dermatitis severity, mainly assessed using SCORAD; impact on quality of life; safety and adverse events; response rate, defined as the proportion of participants achieving clinically meaningful improvement.	Probiotics showed a modest but statistically significant effect on atopic dermatitis severity (mean difference in SCORAD, −5.74; 95% CI, −10.37 to −1.12). Prebiotics and hydrolyzed formulas may have beneficial effects, although the evidence was limited. Supplementation with vitamins, minerals, and fatty acids, including omega-3, did not consistently improve clinical outcomes. Subgroup analyses suggested some benefit of probiotics in children, but not in adults. The overall certainty of the evidence was low to very low because of risk of bias and study heterogeneity.	Nutritional and dietary interventions, particularly probiotics, may provide modest improvements in atopic dermatitis severity, especially in children. However, the evidence remains limited and heterogeneous, and no consistent benefit was demonstrated for vitamins, minerals, or fatty acid supplementation. Further high-quality, adequately powered trials are needed to clarify the role of dietary strategies in atopic dermatitis management.	3
Kawamoto et al. 2025 [72]	Yes	To evaluate the efficacy and safety of targeted systemic therapies, including biologics and small molecules, for moderate-to-severe atopic dermatitis in children.	Children aged ≤ 18 years with atopic dermatitis.	Primary efficacy outcome: EASI-75 response. Primary safety outcome: treatment-emergent adverse events (TEAEs). Secondary outcomes included IGA 0/1, POEM, SCORAD-50, CDLQI, DFI, and other validated measures.	Targeted systemic therapies significantly improved EASI-75 response (risk ratio [RR], 2.99; 95% CI, 2.66–3.37). Favorable effects were also observed for IGA 0/1 (RR, 3.77), POEM-4 (RR, 2.25), SCORAD-50 (RR, 2.70), and NRS-4 (RR, 3.64). Treatment-emergent adverse events were slightly more frequent overall (risk difference [RD], 0.05), particularly with small molecules (RD, 0.14), but not with biologics (RD, −0.06). No significant increase was observed in serious adverse events, treatment discontinuation, or mortality.	Targeted systemic therapies, particularly biologics such as dupilumab, are effective and relatively safe in children with moderate-to-severe atopic dermatitis. Small molecules show comparable efficacy but may be associated with a slightly higher risk of adverse events. These therapies represent a valid option for refractory cases, although long-term and comparative studies are needed to guide treatment selection.	1

**Table A4:** Summary of guidelines included in the analyses.

Study	Objectives	Key Messages	PICO
**IgE-Mediated and Non-IgE Mediated Food Allergy and Eosinophilic Gastrointestinal Disease**			
Halken et al. 2021 [23]	To review current strategies for the prevention of food allergy in infants and children.	Evaluation of preventive interventions aimed at reducing the risk of food allergy and formulation of practical, evidence-based clinical recommendations for food allergy prevention.	1, 2
Santos et al. 2023 [2]	To provide recommendations for the early diagnosis of IgE-mediated food allergies.	Recommendations on the diagnostic use of oral food challenge (OFC), particularly in equivocal cases, and its integration with standard diagnostic tests.	2
Stiefel et al. 2017 [113]	To provide recommendations for the early diagnosis of peanut and tree nut allergy.	Clinical significance and diagnostic value of allergen-specific IgE testing for peanut and tree nuts, with or without a positive skin prick test (SPT), together with practical recommendations for patient management.	2
Matthai et al. 2020 [114]	To provide evidence-based recommendations for the prevention and management of cow’s milk protein allergy (CMPA).	Clinical and diagnostic value of allergen-specific IgE measurements and skin prick testing (SPT). Clinical significance of the response to an elimination diet in the diagnostic work-up of CMPA. Dietary management of CMPA through elimination diets, including the appropriate use of soy-based formulas when suitable to ensure adequate growth and nutritional status.	2
Martelli et al. 2021 [115]	To evaluate the role of component-resolved diagnostics (CRD) in the risk assessment and diagnosis of food allergy.	Assessment of the added diagnostic value of component-resolved diagnostics (CRD) when used in combination with conventional sensitization tests. Evaluation of the potential of CRD to reduce the need for oral food challenges (OFCs). Assessment of the usefulness of molecular allergen diagnostics for identifying children at increased risk of severe allergic reactions, predicting the development of the allergic march, and evaluating the clinical relevance of cross-reactivity. When combined with conventional sensitization tests, component-resolved diagnostics (CRD) improve diagnostic performance and may reduce the need for oral food challenges (OFCs). Molecular allergen profiling can help identify children at increased risk of severe food-allergic reactions, particularly those sensitized to lipid transfer proteins (LTPs) and seed storage proteins. In addition, CRD may provide valuable information for predicting the progression of the allergic march and assessing the clinical significance of allergen cross-reactivity.	2
Berni Canani et al. 2022 [29]	To develop an appropriate diagnostic, therapeutic, and care pathway (DTCP) for the management of pediatric food allergies.	To provide a structured diagnostic, therapeutic, and care pathway for healthcare professionals involved in the management of children with suspected food allergy. To facilitate the early recognition and appropriate management of pediatric food allergies. To reduce diagnostic delays, misdiagnosis, and food allergy-related health risks through standardized clinical care. To promote a rational and evidence-based approach to food allergy management, thereby reducing the socioeconomic burden on affected families and healthcare systems Implementation of a standardized diagnostic, therapeutic, and care pathway (DTCP) can improve the early identification and management of pediatric food allergies, reduce diagnostic delays and inappropriate interventions, and enhance patient safety. A structured multidisciplinary approach also promotes more efficient use of healthcare resources while reducing the socioeconomic impact of food allergy on families and the healthcare system.	2
Dellon et al. 2025 [36]	To provide evidence-based diagnostic and therapeutic recommendations for the management of eosinophilic esophagitis (EoE).	Evidence-based recommendations for the diagnosis, treatment, and follow-up of pediatric patients with eosinophilic esophagitis. Assessment of the effectiveness of proton pump inhibitors (PPIs), swallowed topical corticosteroids, empiric elimination diets, biologic therapy, esophageal dilation, and nutritional interventions in selected pediatric populations. Recommendations for long-term monitoring through clinical, endoscopic, and histopathological assessment to evaluate treatment response and guide maintenance therapy. Proton pump inhibitors (PPIs), swallowed topical corticosteroids, empiric elimination diets, biologic therapy, and esophageal dilation are all recommended treatment options for eosinophilic esophagitis, while nutritional therapy may be appropriate in selected pediatric patients. Regular follow-up with clinical, endoscopic, and histopathological evaluations is recommended to assess treatment response and monitor patients receiving maintenance therapy. Both inflammatory activity and fibrostenotic disease should be systematically assessed during the initial evaluation and throughout long-term follow-up.	3
Amil-Dias et al. 2024 [3]	Consensus statement comprising 28 position statements developed by an ESPGHAN panel of pediatric gastroenterology experts and providing evidence-based recommendations for the management of eosinophilic esophagitis (EoE) in children.	Recommendations on the role of biologic therapies in the management of refractory eosinophilic esophagitis. Recommendations on the use of systemic corticosteroids as initial treatment for esophageal strictures before esophageal dilation. Guidance on the assessment and monitoring of health-related quality of life in pediatric patients with EoE. The consensus recommends considering biologic therapies for selected patients with refractory eosinophilic esophagitis and supports the use of systemic corticosteroids as initial therapy in patients with inflammatory esophageal strictures before esophageal dilation when clinically appropriate. The panel also emphasizes the importance of routinely assessing health-related quality of life as part of the comprehensive management and long-term follow-up of children with EoE.	3
Papadopoulou et al. 2024 [35]	To provide evidence-based recommendations for the management of eosinophilic esophagitis (EoE).	Development of recommendations for the diagnosis, treatment, and follow-up of eosinophilic esophagitis, while identifying areas in which evidence remains limited. Current recommendations for the management of eosinophilic esophagitis are constrained by the limited availability of high-quality evidence in several key areas. The guideline highlights the need for individualized clinical decision-making and emphasizes the importance of further well-designed studies to strengthen the evidence base and optimize the diagnosis and management of EoE.	3
**Urticaria and Angioedema**			
Powell et al. 2015 [47]	To provide a practical, up-to-date guide for the clinical assessment, diagnosis, and management of urticaria and angioedema for non-specialist physicians, particularly primary care and emergency medicine clinicians.	The lifetime prevalence of urticaria is approximately 8.8%. Chronic urticaria develops in 30–45% of individuals with urticaria. The prevalence of chronic urticaria among children in the United Kingdom is approximately 3.4%. Urticaria and angioedema occur together in 40–85% of cases, whereas isolated angioedema accounts for approximately 10% of cases. Approximately 30–50% of patients with chronic urticaria have IgG autoantibodies against IgE or the high-affinity IgE receptor (FcεRI), and about 20% have antithyroid antibodies. Approximately 96% of children with chronic urticaria achieve remission within 7 years. Up to fourfold up-dosing of second-generation H1-antihistamines may be required for symptom control, and omalizumab is effective in approximately 80% of patients with antihistamine-refractory chronic spontaneous urticaria. ACE inhibitor-induced angioedema occurs in up to 0.68% of treated patients. Chronic urticaria significantly impairs quality of life through sleep disturbance, anxiety, depression, and reduced school or work productivity, while psychological stress may exacerbate urticaria and angioedema. Diagnosis is primarily clinical, and laboratory investigations should be guided by the patient’s history rather than performed routinely. Second-generation H1-antihistamines are recommended as first-line therapy (Grade A recommendation), with dose escalation up to fourfold when necessary. Omalizumab is the preferred treatment for antihistamine-refractory chronic spontaneous urticaria, whereas tranexamic acid may be considered in selected patients with angioedema without wheals, although supporting evidence remains limited. Oral corticosteroids should be reserved for severe acute exacerbations and are not recommended for long-term treatment. In children, chronic urticaria is generally milder than in adults and often resolves spontaneously, while inducible forms are relatively more common. Autoimmune mechanisms also occur in pediatric patients but appear less frequently than in adults.	1, 2, 3
Maurer et al. 2022 [5]	To provide updated evidence-based recommendations for the diagnosis and management of hereditary angioedema (HAE), with particular focus on type 1 HAE (C1 inhibitor deficiency) and type 2 HAE (C1 inhibitor dysfunction); update and replace previous WAO/EAACI guidelines by incorporating the latest scientific evidence and international expert consensus; address key clinical questions regarding diagnosis, on-demand treatment, long-term prophylaxis, treatment goals, management of special populations (including children and pregnant women), and disease monitoring; and promote global adoption of standardized diagnostic and therapeutic approaches to improve equity of care and access to treatment.	Development of 28 evidence-based clinical recommendations for the diagnosis and management of hereditary angioedema. Standardization of the international approach to HAE diagnosis, treatment, and follow-up. Development of practical decision-making algorithms, evidence grading, disease-control criteria, and patient-reported outcome measures (PROMs). Improvement of the overall clinical management of patients with HAE through internationally harmonized recommendations.	1, 2, 3
Zuberbier et al. 2022 [4]	To update and revise the international urticaria guidelines previously published in 2018; provide evidence-based and expert-guided recommendations for the definition, classification, diagnosis, and management of acute and chronic urticaria, including spontaneous and inducible subtypes; harmonize urticaria care internationally through collaboration among European and Asian scientific societies, involving 64 delegates from 50 scientific societies across 31 countries; apply a rigorous methodology based on Cochrane Collaboration standards, GRADE Working Group principles, and structured consensus processes; and incorporate clinical activity and quality-of-life assessment tools, such as UAS, AECT, and CU-Q2oL, to support a patient-centered approach.	Development of updated international recommendations, based on evidence and expert consensus, for the definition, classification, diagnosis, and management of urticaria.	1, 2, 3
Farkas et al. 2017 [61]	To address the limitations of previous hereditary angioedema (HAE) guidelines, which primarily focused on adults, by developing pediatric-specific international recommendations for patients with C1 inhibitor deficiency (C1-INH-HAE). The guideline aims to provide evidence- and consensus-based recommendations for early diagnosis, management of acute attacks, short- and long-term prophylaxis, patient and family education, and specialist follow-up, while promoting the international standardization of clinical practice.	Development of international consensus recommendations for the diagnosis and clinical management of children and adolescents (0–18 years) with hereditary angioedema due to C1 inhibitor deficiency (C1-INH-HAE).	5

**Table A5:** Quality appraisal of original studies included into the present analysis according to the Newcastle Ottawa Scale (NOS). For detailed description of included item, please refer to the Wells G et al. [116].

	Year	PICO	D1	D2	D3	D4	D5	D6	D7	D8	D9	Score
**IgE-Mediated and Non-IgE Mediated Food Allergy and Eosinophilic Gastrointestinal Disease**												
Miceli Sopo et al. [26]	2019	1, 2	*	-	*	*	-	-	*	*	*	6
Jacob et al. [30]	2023	1, 2	*	-	*	*	-	-	*	*	*	6
Parker et al. [109]	2024	2	*	*	*	*	*	*	*	*	*	9
Goldberg et al. [110]	2024	2	*	-	*	*	-	-	*	*	*	6
Diaz et al. [32]	2022	2	*	*	*	-	-	*	*	*	*	7
Chauveau et al. [25]	2016	1, 2	*	*	*	*	-	*	*	*	*	8
Zivanovic et al. [31]	2017	2	*	-	*	*	-	*	*	*	*	7
Choi BS et al. [41]	2015	3, 4	*	-	*	*	*	-	*	*	-	6
Votto et al. [100]	2023	3	*	-	*	*	*	-	*	*	-	6
Del Mar Vasquez et al. [39]	2022	4	*	-	*	*	-	-	*	-	*	5
Azzano et al. [38]	2019	4	*	-	*	*	*	-	*	*	*	7
Erwin et al. [40]	2015	4	*	-	*	*	-	-	*	-	*	5
Erwin et al. [111]	2017	4	*	*	*	*	*	-	*	-	*	7
Votto et al. [42]	2022	4	*	-	*	*	-	*	*	-	*	6
**Urticaria and Angioedema**												
Kosmeri et al. [57]	2019	3	-	-	*	-	-	-	*	-	-	2
Yilmaz et al. [53]	2017	2, 3	*	-	*	*	*	-	*	*	*	7
Song et al. [48]	2021	1	-	-	*	*	-	-	*	*	*	5
Kasap et al. [49]	2024	1	*	-	*	-	-	-	*	-	*	4
Galletta et al. [50]	2025	1	*	-	*	-	-	-	*	*	*	5
Barzilai et al. [58]	2023	3	*	-	*	-	-	-	*	-	-	3
Le M. et al. [59]	2022	3	*	-	*	-	-	-	*	-	-	3
Buono et al. [54]	2024	2, 3	*	-	*	*	-	-	*	*	-	5
**Atopic Dermatitis**												
Barbarot et al. [92]	2023	2	*	*	-	-	-	-	*	*	*	5
Flohr et al. [83]	2023	1	*	*	*	*	*	*	*	*	*	9
Boguniewicz et al. [87]	2024	1	*	*	*	*	*	*	*	*	*	9
Paller et al. [88]	2024	1	*	*	*	*	*	*	*	*	*	9
Paller et al. [86]	2025	1	*	*	*	*	*	*	*	*	*	9
Minasyan et al. [89]	2015	2	*	*	*	*	*	-	*	*	*	8
Tran et al. [67]	2018	1	*	*	*	*	*	*	*	*	*	9
Hon et al. [94]	2019	1, 2, 4	*	*	-	-	-	-	-	-	-	2
Cheng et al. [90]	2020	2	*	-	-	*	*	-	*	*	-	5
Salava et al. [75]	2021	1, 4	*	*	*	*	*	*	*	*	-	8
Gazibara et al. [76]	2022	1, 2, 4	*	-	*	*	*	-	*	*	*	7
Andrade et al. [82]	2023	1	*	-	*	*	-	-	*	-	*	5
Liming et al. [85]	2023	1	*	-	*	*	-	-	*	-	*	5
Ren et al. [84]	2025	1	*	-	*	*	-	-	*	-	*	5

**Table A6:** Appraisal of the risk of bias of randomized controlled trials included in the analyses according to the National Toxicology Program (NTP)’s Office of Health Assessment and Translation (OHAT) handbook and respective risk of bias (ROB) tool. Note: D1: possibility of selection bias; D2: exposure assessment; D3: outcome assessment; D4: confounding factors; D5: reporting bias; D6: other bias; : definitively high; : probably high; : probably low; : definitively low.

	D1	D2	D3	D4	D5	Overall
**Urticaria and Angioedema**						
Finlay et al. 2017 [51]						
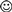
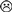
**Atopic Dermatitis**						
Grillo et al. 2006 [80]						
Staab et al. 2006 [79]						
Shaw et al. 2008 [81]						
Pustišek et al. 2016 [74]						

**Table A7:** Qualitative assessment of the studies included in the analysis: systematic reviews with meta-analysis assessed using an adapted version of the AMSTAR 2 tool.

Topic	Study	Documented Protocol (Yes/No)	Adequate Search Strategy (Yes/No)	Selection and Exclusion Strategy (Yes/No)	Risk-of-Bias Assessment (Yes/No)	Risk of Bias Used in the Assessment (Yes/No)	Critical Flaws (Yes/No)	Non-Critical Weaknesses (0–5)	Overall Quality Assessment
IgE-mediated and non-IgE mediated food allergy and eosinophilic gastrointestinal disease	Kelleher et al. 2022 [112]	Yes	Yes	Yes	Yes	Yes	No	0	HIGH
	Riggioni et al. 2024 [33]	Yes	Yes	Yes	Yes	Yes	No	0	HIGH
Urticaria and angioedema	Caffarelli C. et al. 2019 [56]	No	No	No	No	No	Yes	4	VERY LOW
	Di Agosta E. et al. 2021 [52]	No	No	No	No	No	Yes	4	VERY LOW
	Caffarelli C. et al. 2020 [55]	No	No	No	No	No	Yes	4	VERY LOW
Atopic dermatitis	Kim et al. 2016 [66]	Yes	Yes	Yes	Yes	Yes	No	0	HIGH
	Hill et al. 2016 [91]	Yes	Yes	Yes	Yes	Yes	No	0	HIGH
	Li et al. 2020 [77]	Yes	Yes	Yes	Yes	Yes	No	0	HIGH
	Oykhman et al. 2022 [97]	Yes	Yes	Yes	Yes	Yes	No	0	HIGH
	Yepes-Nunez 2023 [96]	Yes	Yes	Yes	Yes	Yes	No	0	HIGH
	Andrade et al. 2024 [78]	Yes	Yes	Yes	Yes	Yes	No	0	HIGH
	Vassilopoulou et al. 2024 [98]	Yes	Yes	Yes	Yes	Yes	No	0	HIGH
	Kawamoto et al. 2025 [72]	Yes	Yes	Yes	Yes	Yes	No	0	HIGH

**Table A8:** Qualitative assessment of the guidelines included in the analysis: clinical practice guidelines were appraised using an adapted version of the AGREE II instrument.

Topic	Guideline	Overall Quality of Guideline Development Methods (1–7)	Overall Quality of Guideline Presentation (1–7)	Completeness of Reporting (1–7)	Overall Quality of Guideline Recommendations (1–7)	Overall Guideline Quality (1–7)	I Would Recommend This Guideline for Clinical Practice (1–7)	I Would Use This Guideline to Inform My Clinical Decision-Making (1–7)
IgE-mediated and non-IgE mediated food allergy and eosinophilic gastrointestinal disease	Halken et al. 2021 [23]	5	5	6	6	6	6	6
	Santos et al. 2023 [2]	5	5	6	6	5	5	5
	Stiefel et al. 2017 [113]	5	5	5	6	6	6	6
	Matthai et al. 2020 [114]	6	6	6	6	6	6	6
	Martelli et al. 2021 [115]	6	6	7	7	6	7	7
	Berni Canani et al. 2022 [29]	6	7	6	6	7	6	7
	Dellon et al. 2025 [36]	7	7	7	7	7	7	7
	Amil-Dias et al. 2024 [3]	7	7	7	7	7	7	7
	Papadopoulou et al. 2024 [35]	7	7	7	7	7	7	7
Urticaria and angioedema	Powell et al. 2015 [47]	7	7	7	7	7	7	7
	Maurer et al. 2022 [5]	7	7	7	7	7	7	7
	Zuberbier et al. 2022 [4]	6	4	4	4	4	4	4
	Farkas et al. 2017 [61]	5	4	4	4	4	4	4
